# Perturbation Centrality and Turbine: A Novel Centrality Measure Obtained Using a Versatile Network Dynamics Tool

**DOI:** 10.1371/journal.pone.0078059

**Published:** 2013-10-21

**Authors:** Kristóf Z. Szalay, Peter Csermely

**Affiliations:** Department of Medical Chemistry, Semmelweis University, Budapest, Hungary; University of Maribor, Slovenia

## Abstract

Analysis of network dynamics became a focal point to understand and predict changes of complex systems. Here we introduce Turbine, a generic framework enabling fast simulation of any algorithmically definable dynamics on very large networks. Using a perturbation transmission model inspired by communicating vessels, we define a novel centrality measure: perturbation centrality. Hubs and inter-modular nodes proved to be highly efficient in perturbation propagation. High perturbation centrality nodes of the Met-tRNA synthetase protein structure network were identified as amino acids involved in intra-protein communication by earlier studies. Changes in perturbation centralities of yeast interactome nodes upon various stresses well recapitulated the functional changes of stressed yeast cells. The novelty and usefulness of perturbation centrality was validated in several other model, biological and social networks. The Turbine software and the perturbation centrality measure may provide a large variety of novel options to assess signaling, drug action, environmental and social interventions.

## Introduction

In the last decade the network approach became a widely used methodology to study complex systems. As an example, protein structure networks, where network nodes represent amino acids, and edges symbolize their physical distance, are increasingly used to describe conformational changes, formation of protein complexes, drug binding and enzyme action [1]–[3]. Recently several programs have been introduced for the construction of protein structure networks from 3D structural data and for their analysis [4], [5]. Protein-protein interaction networks (interactomes) provide a great help to understand the molecular mechanism of cellular functions, the development of diseases and drug design [6]. In protein-protein interaction networks such as BioGRID [7], STRING [8], and DIP [9], nodes are proteins, and edges denote their physical interactions.

Network dynamics is necessary to understand the changes of complex systems, and therefore became a hot topic of network studies [10], [11]. A number of programs have been developed for the calculation of certain aspects of network dynamics, such as network simulation tools based on Boolean dynamics [12]–[16], the random-walk based ITM-Probe [17], the law of mass action-based PerturbationAnalyzer [18], or the biological system modeling tool, BIOCHAM [19]. However, to our knowledge, no stand-alone program exists, which can easily integrate any dynamical models together with any types of starting perturbations, and can also provide the complete time-domain simulation results, not only the summative end-result. Recently the complex network dynamics simulation tool, Conedy was introduced [20]. Conedy is a Python extension enabling researchers already using computational dynamics to add networks to their repertoire. However, a complete toolkit is still missing having built-in algorithms, analysis tools and visualization, to enable life and network scientists to add network dynamics to their studies.

Our in-house developed program, Turbine, is able to accommodate a large variety of network dynamic simulations. Any real-world network can be imported to the program and perturbations can be introduced at any nodes or node-combinations at the beginning, at any time during the simulation, individually, repeatedly, or continuously. This allows the analysis of the effect of different environmental changes on network dynamics. Computational optimizations allow the simulation of large networks (in the range of millions of nodes and edges) on a standard desktop-grade computer. The initial phase of Turbine development was mentioned in an earlier conference lecture summary [21]. Here we introduce the fully developed program (freely downloadable from here: http://turbine.linkgroup.hu), and show that its results on the importance of hubs and inter-modular nodes in the propagation of perturbations reflect well both our intuitive expectations and former knowledge. We defined a new measure of dynamic network centrality, and termed it as perturbation centrality. Perturbation centrality correctly identified substrate binding sites and amino acids participating in allosteric signaling in protein structure network networks. Changes of perturbation centrality were in agreement with the known functional changes of the yeast protein-protein interaction network after stress. The novelty and usefulness of perturbation centrality was validated in several other model, biological and social networks. Turbine is an integrative and versatile tool to simulate network dynamics and to predict the effects of environmental changes, signaling stimuli, drugs or social interventions.

## Results

In the simulations using our network dynamics program called Turbine, we used a model termed “communicating vessels”. The basic idea behind this model was that intensive physical variables (e.g. temperature) tend to perform an equalization-like dynamics behaving like communicating vessels (see the detailed description in **Methods**). The communicating vessels model gives an exponential decay of perturbations (see **Supplementary Results** of **Text S1**), which is in agreement with several earlier findings [22], [23].

### Modules limit perturbation propagation

**Figure 1:**
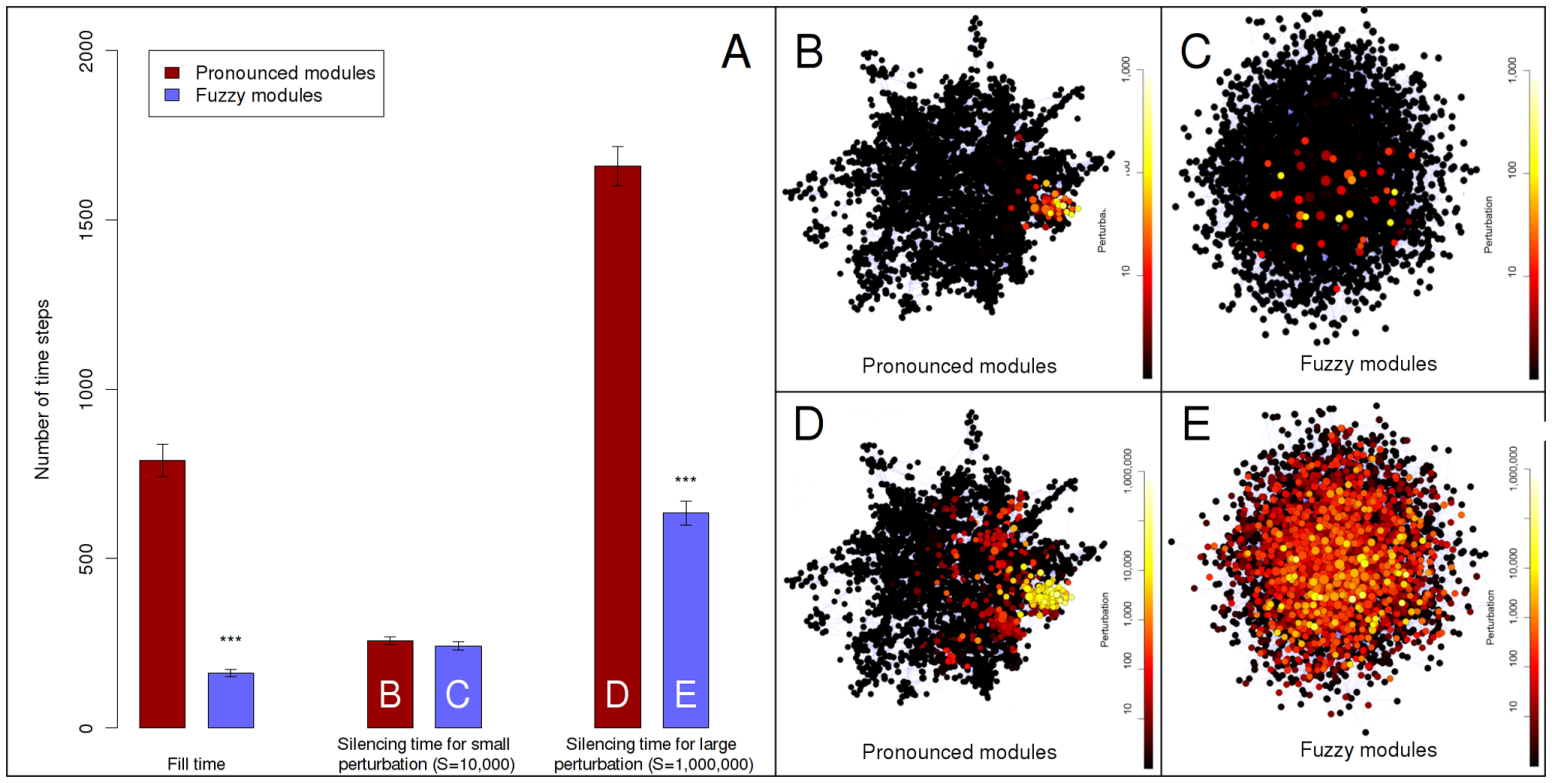
Difference in perturbation propagation between benchmark graphs with pronounced and fuzzy modules. Two times 7 randomly selected scale-free, modular benchmark graphs [28] were generated as described in **Supplementary Methods** and **Table S7** of **Text S1** with ratios of inter-modular edges of 0.05 (∼300 of ∼6,000 edges were inter-modular) and 0.4 (∼2400 of ∼6,000 edges were inter-modular), termed as “pronounced modules” and “fuzzy modules”, respectively. Panel A: average fill times and silencing times, separately for the “fuzzy” and the “pronounced” group of networks. Fill times and silencing times were determined as described in **Methods**. Continuous perturbation intensity for fill time was 10,000 units, while initial perturbation intensities for silencing times were 10,000 or 1,000,000 units at low intensity or at high intensity perturbations, respectively. The three asterisk signs mark statistically significant differences with α = 0.001. Dark red bars and light blue bars represent pronounced modules and fuzzy modules, respectively. Bar letter codes refer to Panels showing snapshots of perturbations with identical conditions. Panels B through E show image snapshots created by the Turbine viewer after 50 time-steps of the simulation, using a heat-based color map. (The order of colors marking the lowest to highest perturbation is: black → red → orange → yellow → white). Perturbation values were scaled logarithmically. Panels B and C show the effect of low intensity starting perturbations (S = 10,000), while Panels D and E show the effect of high intensity starting perturbations (S = 1,000,000). Panels B and D show benchmark graphs with pronounced modules, while Panels C and E show benchmark graphs with fuzzy modules.

The intuitive impression that modules limit the information spread in complex networks was described in multiple papers, and was shown in many simulations [24]–[27]. The equations of the communicating vessels model (where every affected node dissipates an equal amount of energy in every time-step of the simulation) suggest that the more nodes are affected by a given perturbation, the faster the perturbation becomes dissipated. Taken together these two considerations, our expectation was that a network with rather distinct modules (termed as pronounced modules) will propagate and dissipate perturbations slower than a network with tightly interconnected modules (termed as fuzzy modules). To test whether the communicating vessels model of Turbine shows this property, we used the benchmark graph generating tool of Lancichinetti and Fortunato [28] to generate unweighted and undirected scale-free, modular benchmark networks (hereafter called as benchmark graphs) with different ratios of inter-modular edges (**Table S7** of **Text S1**). The benchmark graph with pronounced or fuzzy modules had 5% or 40% of inter-modular edges, respectively. We have used a new measure termed “fill time” for comparison. The fill time of node *i* is the time needed to raise the energy level of 80% of the nodes above 1 unit while continuously adding energy to node *i*. **Figure 1A** shows fill times calculated on 7 random generations of these benchmark graphs using the same perturbations starting from each node in separate simulations. Fill times of all nodes and all 7 benchmark graphs with different random seeds were averaged. As expected, sparse inter-modular edges of the pronounced modules delayed the propagation of perturbation, resulting in a 4.8 times larger fill time as compared to those observed in benchmark graphs with fuzzy modules (**Figure 1A**). **Supplementary Results** in **Text S1** show that fill time is highly correlated with closeness centrality, making fill time useful as a verification of the model rather than a novel centrality measure.

Next we assessed the propagation of single perturbations using the same model, but adding a dissipation term to the communicating vessels dynamics. **Figures 1B through 1E** are illustrations of the propagation of dissipated perturbations using image snapshots of the Turbine viewer program after 50 time-steps of the simulation. The starting module of the benchmark graphs with pronounced modules trapped the initial perturbation, if the size of the perturbation was sufficiently high (1,000,000 units, **Figure 1D**). This ‘module encapsulation’ effect was entirely missing from the benchmark graphs with fuzzy modules (**Figures 1C and 1E**), and was also not observable, when the starting perturbation was low intensity (10,000 units). Thus, roughly the same number of nodes was affected by the perturbation in benchmark graphs having either pronounced (**Figure 1B**) or fuzzy modules (**Figure 1C**) if the initial perturbation was low-intensity (10,000 units). On the contrary, a high-intensity starting perturbation (1,000,000 units) affected much less nodes in benchmark graphs having pronounced modules (**Figure 1D**) as compared to those having fuzzy modules (**Figure 1E**). After applying a high intensity perturbation to benchmark graphs with fuzzy modules almost all nodes became perturbed after the 50 time-steps shown (**Figure 1E**).

The right two sets of bars of **Figure 1A** show the differences in perturbation dissipation in a quantitative manner. Here a measure termed “silencing time” was calculated as the first time step when all nodes had an energy value less than 1. The same perturbation was started from each node of the 7 random representations of benchmark graphs in separate simulations, and their silencing times were averaged for all nodes and for all the 7 graphs. Bars with capital letters refer to the benchmark graphs shown on **Figure 1B through 1E** **.** Benchmark graphs with pronounced modules dissipated low intensity perturbations only slightly slower than benchmark graphs with fuzzy modules (cf. bars “B” and “C” on **Figure 1A**). This is in agreement with the approximately same number of nodes affected after 50 time-steps in the same pair of simulations (**cf.** **Figures 1B and 1C**). On the contrary, high intensity perturbations were dissipated dramatically (2.6 times) slower by benchmark graphs with pronounced modules as opposed to those with fuzzy modules (cf. bars “D” and “E” on **Figure 1A**). These results clearly indicated that pronounced modular structures trap perturbations in agreement with earlier results [27]. The difference between the behavior of low-, and high-intensity perturbations arises from the fact that perturbations are decaying exponentially with the distance from their origin (for a proof see the analysis of **Text S1**). Thus, when the perturbation is of relatively low-intensity (compared to the size of the module and the dissipation rate) it is dissipated without reaching the boundaries of its module of origin. In the case of high-intensity perturbations, a significant amount of energy transverses the boundary of its module of origin, which makes the modular-encapsulation effect observable. Module encapsulation of perturbations was also tested using the random-walk based ITM-Probe method [17], with very similar results (see **Supplementary Results**, **Table S7** of **Text S1**, and **Figures S1 through S3** of **Text S1**).

### Definition of perturbation centrality as the reciprocal of silencing time

Prompted by the utility of silencing time to characterize the propagation and dissipation of perturbations (**Figure 1**), and utilizing our former observation that the reciprocal of fill time was correlated with closeness centrality (**Table S1** of **Text S1**), we defined a centrality-type measure for dissipated perturbations, and termed it as perturbation centrality. Our aim was to conceive a measure that takes into account both local (weighted degree) and more global (modular position) perturbation properties. It was also important that the measure should be easy to understand and calculate, and that its properties should be almost independent of the size of the network. As a result of our initial studies (**Table S2** of **Text S1**) we have found that setting the initial perturbation value to 10*n units (*n* being the number of nodes in the network) achieves all of these goals. Thus, perturbation centrality of node *i* was defined as the reciprocal of silencing time retrieved by using a Dirac delta type starting perturbation of 10*n units, where *n* is the number of nodes in the network, using a dissipation value of 1. Silencing time of node *i* was the first time when all of the nodes had an energy value less than a pre-set minimal threshold after an initial perturbation started from node *i*. We selected this threshold as “1”, the minimum sensible value with the dissipation also being set to 1 (note that the dissipation value is the minimal threshold, since after reaching this value all energy of the network will be dissipated in the next step).

Following the above definition, we have tried to find the location of our newly conceived perturbation centrality measure on the “centrality landscape” by testing its correlation with established centrality measures in selected networks. We have tested perturbation centrality against closeness centrality, which is the average geodesic distance from the given node to all other nodes; betweenness centrality, the number of shortest paths between any two nodes passing through the tested node; community centrality [29], a measure high in the cores of network communities and PageRank, an iterative measure coined by Brin and Page [30], where nodes “vote” on each other *via* their edges in proportion with their degree. We have also tested the correlations between perturbation centrality and degree (as well as weighted degree), since these measures can also be interpreted as local centrality measures. These correlations between perturbation centrality and other centrality-type measures of different real-world networks are shown in **Table 1**. There was a considerable correlation between perturbation and closeness centralities. However, the strength of this correlation was noticeably less than that between the reciprocal of fill time and closeness centrality (average correlations were 67% and 89.5%, respectively, with explained change, R^2^ values of 45% and 80%, respectively). A similarly high correlation was observable between perturbation and community centralities, as well as between perturbation centrality and weighted degree suggesting that nodes in key community locations and/or hubs may be among the best dissipators of perturbations. Correlations between perturbation centrality and either PageRank or betweenness centrality were smaller (but noticeable) than that between perturbation and closeness centralities.

**Table 1:** Correlation between perturbation centrality and other centrality measures.

Networks^a^	Closeness centrality	Betweenness centrality	Community centrality^b^	PageRank^c^	Degree	Weighted degree
Benchmark graphs with pronounced modules	**0.79**	0.31	0.30	*0.08*	*0.26*	*0.26*
Benchmark graphs with fuzzy modules	**0.79**	**0.76**	**0.83**	0.67	**0.83**	**0.83**
Substrate-free Met-tRNA synthetase protein structure network	**0.86**	0.44	0.26	0.16	0.38	0.34
Substrate-bound Met-tRNA synthetase protein structure network	**0.87**	0.44	0.25	0.18	0.41	0.37
Filtered Yeast Interactome	*0.09*	0.33	**0.80**	0.47	0.67	**0.85**
Database of Interacting Proteins yeast interactome (release 2005)	0.62	0.56	**0.84**	**0.73**	0.66	**0.72**
Database of Interacting Proteins yeast interactome (release 2010)	0.67	0.41	0.63	0.47	0.52	0.65
*E. coli* metabolic network	**0.72**	0.31	**0.97**	0.67	0.59	**0.99**
*B. aphidicola* metabolic network	**0.70**	0.40	**0.98**	**0.78**	**0.72**	**0.99**
School-friendship network	0.68	0.43	0.69	0.58	0.68	**0.71**
**Mean and standard error**	**0.67 (0.063)**	**0.44 (0.043)**	**0.65 (0.090)**	**0.48 (0.081)**	**0.57 (0.056)**	**0.69 (0.087)**

Perturbation centrality was compared to other centrality measures calculated as described in **Supplementary Methods** of **Text S1**. Spearman correlations above r = 0.7 are marked with bold letters, correlations below r = 0.3 are marked with italics. Highest correlations were observed between perturbation centrality *versus* closeness centrality, community centrality [29] and weighted degree. This underlines the observations that besides geodesic distance (closeness centrality), modular position and degree also contribute to good perturbation properties. Note that measured correlations between perturbation and closeness centralities are much weaker than the correlations between the reciprocal of fill time and closeness centrality (mean is 0.895 in **Table S1** compared to 0.67 here, p = 0.000487, Wilcoxon rank-sum test; correlations with closeness centrality failed the Shapiro normality test with p = 0.0019)

a Network descriptions are given in **Supplementary Methods** and **Table S7** of **Text S1**.

b Community centrality was calculated using the LinkLand community detection method of the ModuLand family as described by Kovács *et al*. [29].

c PageRank values were calculated using the algorithm of the igraph library [59].

On one hand, data of **Table 1** indicate that perturbation centrality is a more local centrality measure than closeness centrality. On the other hand perturbation centrality is a more global centrality measure than weighted degree or PageRank. Thus perturbation centrality is a novel, mesoscopic-type centrality measure characterizing the information transfer capability of a given node (or edge: see **Supplementary Results** and **Figure S10** of **Text S1**) in a network. A visual representation of the relationship between perturbation centrality and the other centrality measures shows a unique position of perturbation centrality further supporting the novelty of perturbation centrality (**Figure S4** of **Text S1**).

### Hubs and inter-modular nodes have a central role in perturbation dissipation

**Figure 2:**
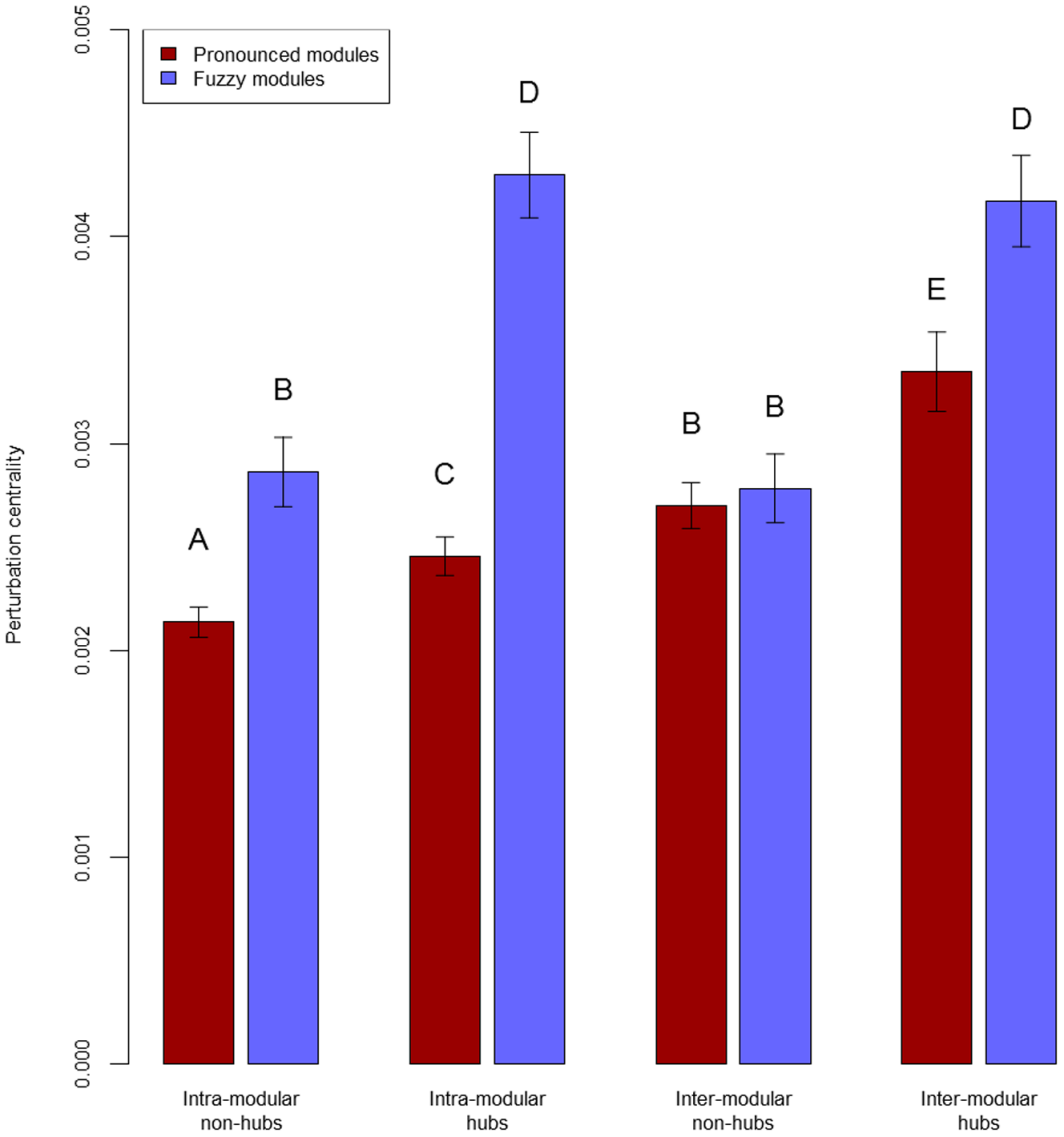
Average perturbation centralities for different node types in benchmark graphs. Scale-free, modular benchmark graphs [28] were created as described in **Supplementary Methods** and **Table S7** of **Text S1**. Average perturbation centralities were calculated as described in **Methods** using a starting perturbation of 40,000 units, since the benchmark networks contained 4,000 nodes. 4 node types were discriminated: intra-modular non-hubs, inter-modular non-hubs, intra-modular hubs and inter-modular-hubs, where hubs were nodes having a degree in the top 10%, and inter-modular nodes were nodes with more than 40% inter-modular edges. Different letters on top of the bars mark significantly different groups with α = 0.01 (Wilcoxon rank-sum test). Dark red bars show results obtained using 7 randomly selected benchmark graphs with the ratio of inter-modular nodes set to 0.05, termed as pronounced modules, while light blue bars display data for 7 randomly selected benchmark graphs (with the same seed nodes as the ones used for pronounced modules) with ratio of inter-modular nodes set to 0.4, termed fuzzy modules.

Next we investigated which types of nodes are best for dissipating perturbations. On one hand, we observed large differences in the average perturbation efficiency of modular networks with differing ratios of inter-modular nodes (**Figure 1**). On the other hand, hubs have been proven to have a high information transmission efficiency [31], [32]. Based on these considerations we defined 4 node categories: 1.) intra-modular non-hubs; 2.) intra-modular hubs; 3.) inter-modular non-hubs and 4.) inter-modular hubs. We defined hubs as nodes with degrees in the top 10%, and inter-modular nodes as nodes with at least 40% inter-modular edges. Intra-modular non-hubs correspond to roles R1 and R2 (ultra-peripheral and peripheral nodes) in the previous representation of Guimerà *et al*. [33]; intra-modular hubs correspond to role R5 (provincial hubs), while inter-modular non-hubs and inter-modular hubs correspond to roles R3/R4 (non-hub connectors and kinless nodes) and R6/R7 (connector and kinless hubs), respectively. **Figure 2** shows that, in agreement with our expectations, inter-modular non-hubs had a larger perturbation centrality than intra-modular non-hubs in benchmark networks with pronounced modules. Importantly, in networks with pronounced modules inter-modular non-hubs had larger perturbation centrality than intra-modular hubs. On the contrary, in benchmark networks with fuzzy modules hubs in any modular position had a larger perturbation centrality than non-hubs. These observations are again in agreement with expectations and earlier findings [27]. The explanation of these findings is that in fuzzy modules the modular structure did not restrict the propagation of perturbations, so it is not surprising that intra-modular hubs dissipated perturbations faster than inter-modular non-hubs.

Importantly, there was a large (87%) difference between the perturbation centrality of intra-modular hubs versus the effect of inter-modular non-hubs in a wide variety of real-world networks (**Supplementary Results** and **Table S8** of **Text S1**), suggesting that from a perturbation perspective real-world networks resemble the benchmark graphs with fuzzy modules more, than the benchmark graphs with pronounced modules. (Note that the same observation was obtained, when we compared the low-intensity and high-intensity silencing times – see **Table S2** of **Text S1**).

### Perturbation centrality uniquely identifies all key regions of Met-tRNA synthase

Prompted by the general applicability of the perturbation centrality measure to characterize real-world networks, next we compared the perturbation centralities with structural and functional properties of nodes in two types of biological networks in detail. The comparison of substrate-free with substrate-bound forms of proteins gives an important system to study the changes in perturbation differences in the respective protein structure networks.

**Figure 3:**
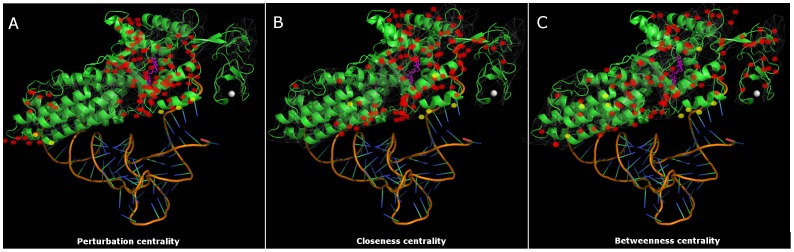
Substrate binding-induced perturbation centrality changes mark important residues of *E. coli* Met-tRNA synthetase. Protein structure networks of the substrate-free and substrate-bound forms of *E. coli* Met-tRNA synthetase protein were generated as described in the **Supplementary Methods** of **Text S1**. Perturbation centralities and the underlying protein structure network of Met-tRNA synthetase were calculated and visualized by the Turbine program as described in **Methods**, and were overlaid on the 3D image of the substrate-bound form of the protein (and its tRNA^Met^ complex) generated with PyMOL [58] using ray-tracing. The bottoms of the images show the structure of tRNA^Met^. The purple molecule in the middle of the protein structure is the substrate Met-AMP marking the active site of the enzyme, the white sphere on the right is the Zn^2+^ ion. Red signs of Panels A, B and C mark amino acids having the highest *increase* of perturbation, closeness and betweenness centralities (top 20%) of the substrate-bound form compared to the substrate-free form, respectively. Yellow signs mark those contact amino acids, which are directly bound to the tRNA^Met^, evidenced by an atomic distance of less than 4.5Å between any atom of the residue and the tRNA^Met^, excluding hydrogens. To avoid overcrowding the image, only those contact amino acids are shown, which have a high increase of their centrality. A separate image showing all tRNA^Met^-binding amino acids is shown in **Figure S9 of Text S1**. Note that red-labeled amino acids having the largest increase of perturbation centrality upon substrate binding (Panel A) are clustered around the active site and around both tRNA-binding sites, thus successfully discriminate all important parts of the protein. Amino acids showing the highest change in closeness centrality (Panel B) are smeared around the active site (which also occurs to be near the geometric center of the protein). Amino acids showing the highest change in betweenness centrality (Panel C) are scattered all around the protein.

**Figure 3** shows residues with top 20% increase of different centralities upon substrate binding of Met-tRNA synthase. Red and yellow symbols of **Figure 3A** represent residues with highly increased *perturbation* centrality. Residues marked with yellow symbols are within a distance of 4.5Å from the tRNA^Met^. Note that perturbation centrality increase highlights the active site and both binding sites of the tRNA. On the contrary, residues with highly increased *closeness* centrality (**Figure 3B**) are smeared around the active site, and residues with the highest increase of *betweenness* centrality (**Figure 3C**) are scattered all around the protein. The fact that perturbation centrality was increased most at the two substrate binding sites of tRNA^Met^ upon binding, indicates that substrate-induced changes in protein structure help a better spread of perturbations caused by substrate binding. This self-amplification may be an important contributor to the propagation of binding-induced conformational changes and allosteric mechanisms.

The tight secondary structures of α-helices had larger perturbation efficiency than the more flexible loops. Importantly, perturbation centrality proved to be better at distinguishing secondary structures, signaling amino acids, as well as amino acids whose importance was proven experimentally than betweenness or closeness centralities (**Figures S6** through **S8** and **Table S9** of **Text S1**).

### Various stress types induce different perturbation dissipating regions of the yeast interactome

As a continuation of the characterization of substrate-induced changes in protein structure networks, we assessed perturbation centralities in a well-characterized change of the interactome. In our earlier studies stress-induced changes in mRNA expression resulted in a marked re-configuration of yeast interactome modules [34], [35]. Here we calculated perturbation centralities for all nodes in the Database of Interacting Proteins yeast interactome (release 2005), using stress-induced mRNA changes [36], [37] to calculate the edge-weights of the stressed yeast interactome as described before [35]. The observation of Mihalik and Csermely [35] that communities of the interactome become more separated under heat shock is expected to induce a lower average perturbation centrality (due to the module encapsulation effect shown before hindering the propagation of perturbations). Indeed, a major change was observed: the average perturbation centrality of the heat-shocked interactome was 6.07*10^−4^, 40.5% lower than the average perturbation centrality of the unstressed interactome (1.02*10^−3^, α = 0.05, p = 2.2*10^−16^, Wilcoxon rank-sum test).

**Figure 4:**
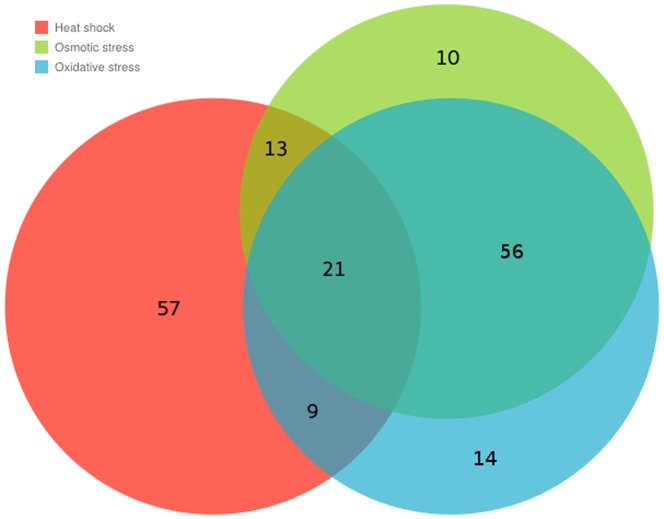
Visualization of the difference among the three top 100 sets of proteins having the highest perturbation centrality in the DIP (2005) yeast interactome. Perturbation centralities were calculated for three stressed variations of the DIP (2005) yeast interactome according to **Methods**. The properties of the network as well as the method of generating its stressed versions are described in the **Supplementary Methods**. The sizes of the different areas of the diagram are roughly proportional to the number of proteins in the respective combination of the three sets. Numbers also show the number of proteins in different sets. This quantitative Venn diagram was generated using the Google Charts API. (https://developers.google.com/chart/image/docs/gallery/venn_charts). The red, green and blue circles show the sets of top 100 proteins having the highest perturbation centrality in the heat-shocked, osmotically- stressed and oxidatively- stressed networks, respectively. This figure illustrates the fact mentioned in the Section “Various stress types induce different perturbation dissipating regions of the yeast interactome” that the most important proteins in heat shock are substantially different from the most important proteins in the other two tested stress types (i.e. in osmotic and oxidative stresses).

We also observed a marked difference of the proteins with highest perturbation centrality in heat-shock compared to the other stress types. Only 42 of the 100 unstressed top perturbation centrality nodes appeared in the top 100 nodes of heat-shocked cells. However, 65 of the unstressed top nodes appeared in the oxidatively-stressed interactome, and 68 in the osmotically-stressed interactome. At the same time, 77 of the top 100 perturbation centrality nodes were the same in the oxidatively- and the osmotically-stressed interactome, while the number of matching nodes was only 30 and 34, when we compared the oxidatively- and the osmotically-stressed interactome against the heat-shocked interactome, respectively. These results are visualized in the Venn-diagram of **Figure 4**. These data prompted us to perform a detailed investigation of the functions of the top perturbation dissipator proteins in unstressed and stressed yeast interactomes.

### Functional assessment of key perturbation dissipator proteins in unstressed and stressed yeast interactomes

For the assessment of the function of proteins having the highest perturbation centrality in the yeast interactome before and after various types of stresses, we used the g:Profiler tool [38], which performs a statistical enrichment analysis to find over-representation of Gene Ontology terms, biological pathways, or regulatory DNA elements in a set of genes or proteins. Only those terms were taken as significant, where the p-value was less than 0.05 after applying Bonferroni correction. Using this method, the top 100 nodes having the largest perturbation centrality in the unstressed interactome had 11 enriched terms, which was extended differently in heat shock and oxidative/osmotic stress (**Table S3** of **Text S1**).

The enrichment analysis on the nodes having the top 100 largest *increase* in perturbation centrality in heat-shock, oxidative and osmotic stress resulted in 28, 22 and 34 enriched terms, respectively. *Carbohydrate metabolism*, *trehalose metabolic process* and *glycogen metabolic process* were upregulated in all types of stresses, which is in agreement of previous findings [39]. Importantly, the term *response to stimulus* appeared in all three types of stresses, and *response to stress* appeared in heat-shock and osmotic stress (**Table S4** of **Text S1**). Assessment of the function of proteins having the largest *decrease* in their perturbation centralities in various stress conditions indicated the down-regulation of *ribosome synthesis* and *protein translation* after stress (**Table S5** **Text S1**), which are also well-known changes in stress [40].

Our results on protein structure and protein-protein interaction networks highlight the usefulness of perturbation centrality to identify functionally important nodes in biological networks, and show that the preferred way of comparing perturbation centralities in two similar networks is to compare the largest *changes* rather than the largest absolute values.

## Discussion

We introduced a new method for the analysis of network dynamics. This new software called “Turbine” (http://turbine.linkgroup.hu) substantially extends the preliminary version of the program published as a conference summary [21]. A dynamic model termed “communicating vessels” was created to assess the propagation of perturbations in networks. To characterize network properties, two new measures were defined. “Fill time” characterizes the propagation-efficiency of un-dissipated perturbations. “Perturbation centrality” of a node is defined as the reciprocal of the time characterizing the dissipation of a perturbation starting from the given node in the network. Both the reciprocal of fill time and perturbation centrality were shown to be centrality-type measures. Fill time correlated well with closeness centrality. On the contrary, perturbation centrality could not be substituted with any standard network centrality measure. Perturbation centrality correctly identified hubs and bridges (inter-modular nodes) as key determinants of the speed of perturbation dissipation. Nodes having a high importance in the information transmission in protein structure networks and in protein-protein interaction networks were also characterized by high perturbation centrality values.

Network dynamics has already been assessed using a number of computational tools. Boolean dynamics [12]–[16] is a binary model, where every node can assume either an active or an inactive state, making Boolean dynamics a generalization of cellular automata on complex networks. Despite its simplicity, Boolean dynamics has been very successful in modeling cellular signaling networks, and identifying underlying causes of pathogenic responses [13], [41]. However, there are also a handful of programs for non-Boolean dynamics. ITM-Probe [17] is based on random-walks; PerturbationAnalyzer [18] is a Cytoscape plug-in using the law of mass action; BIOCHAM [19] and Conedy [20] are more complex network dynamic tools. Turbine combines several advantages of the former options with large versatility, richness of output data, efficient use of memory and fast running time enabling the analysis of large networks (**Table S6** of **Text S1**). Turbine is able to accommodate a large number of other dynamical models than the communicating vessel model used in this paper. Turbine can handle multiple, repeated or continuous perturbations introduced at the beginning of the simulation or at any later time-steps. Moreover, the network structure may also be changed during the simulations.

Despite its apparent simplicity, the communicating vessels model well recapitulated the expected dissipation pattern of hub and inter-modular node perturbations on modular, scale-free benchmark graphs (**Figures 1** and **2**). These results were in agreement with the early assumption of May in 1972 [42] that modular patterns retain the information within a single module and minimize its impact to other modules thus stabilize networks, and they were also in agreement with data published later [24]–[27].

Encouraged by these findings, we defined a novel type of dynamic centrality measure, and termed it as perturbation centrality. Perturbation centrality of node *i* is the reciprocal of the silencing time of node *i* with a starting excitation of 10*n, where *n* is the number of nodes in the network, setting both the dissipation and the silencing threshold to 1. Furthermore, the silencing time of node *i* is the time needed to dissipate the perturbation starting from node *i* at every node below a low residual perturbation threshold. The perturbation centrality measure has a rather straightforward centrality-type meaning. Intuitively thinking, the more central node *i* is, the more nodes are reached by the perturbation started at node *i*. Thus perturbation started from a more central node is dissipated faster – since every node dissipates an equal amount of energy in each step – and so has a smaller silencing time than a perturbation started from a less central node.

Silencing time is not a continuous measure thus the precision of perturbation centrality has a lower bound. However, the parameters of the simulations were set achieving a rather good compromise between calculation speed and the resolution of perturbation centrality. Typical perturbation centrality values ranged from 0.33 (highest) to 0.0001 (lowest) depending on the analyzed network. These values corresponded to silencing times 30 and 10000. Note that the lowest perturbation centrality one can get depends on the number of simulated time-steps, i.e. the lowest perturbation centrality in an experiment with 2000 time steps is 1/2000 = 0.0005. The n*10 starting energy (where *n* is the number of nodes) and the 1 dissipation rate parameters of the perturbation centrality were chosen in order to achieve that nodes in most networks can be characterized by silencing time values between 10 and 10000 steps. This translates to a perturbation centrality value between 0.1 and 0.0001 Thus, these parameters made a good compromise between the total time of simulation and the resolution of the perturbation centrality measure.

All correlations between perturbation centrality and other centrality measures were weaker than the high correlation between the reciprocal of fill time and closeness centrality (cf. **Tables 1** and **Table S1** of **Text S1**; see **Figure S4** of **Text S1** for a graphical representation). Thus perturbation centrality may capture novel dynamics-related features of central nodes (or in a very similar fashion, edges: see **Supplementary Results** and **Figure S10** of **Text S1**). In several case studies on protein structure networks and yeast protein-protein interaction networks we showed that this indeed may be the case. The distribution of perturbation centrality values was different in different networks, which may give an additional layer of network characterization (**Figure S5** of **Text S1**).

Perturbation properties of protein structures revealed by the Turbine model were in agreement with intuitive insights. The tight secondary structures of α-helices had large perturbation efficiency, while the more flexible loops had a lower efficiency of propagating and dissipating perturbations (**Figure S6**). Perturbation centrality discriminated secondary structures slightly better than closeness centrality and much better than betweenness centrality (**Figures S7** and **S8** of **Text S1**). Moreover, perturbation centrality, uniquely of the three tested centralities, highlighted all important segments of Met-tRNA synthase. The substrate binding-induced local increase in perturbation centralities may indicate a self-amplifying cycle, where substrate-induced changes might help a better spread of perturbations caused by substrate binding. Amino acids involved in allosteric communication in Met-tRNA synthetase [43], as well as amino acids with experimentally verified importance in its function [43] had significantly higher than average perturbation centrality in the protein structure network of the enzyme (**Figure S6**; p = 6*10^−8^ and 9.5*10^−8^ for amino acids involved in communication; bound and free conformation, respectively; p = 0.0018 and 0.0022 for amino acids with experimentally verified importance; bound and free conformation, respectively using Wilcoxon rank-sum test, α = 0.0125 adjusted with Bonferroni correction). These findings are in agreement with a number of earlier studies suggesting that perturbation efficiency plays a key role in intra-protein allosteric signaling, as well as showing that both binding sites and inter-domain, bridging amino acids play an especially important role in this process [44]–[47].

Differences between perturbation centralities in the interactomes of unstressed and stressed yeast (**Tables S4 and S5** of **Text S1**) were in agreement with our earlier data on the modular rearrangements of the yeast interactome upon stress [34], [35] and with experimental data showing the down-regulation of yeast ribosome biogenesis and mRNA translation [40], as well as the up-regulation of carbohydrate metabolism [39] after stress.

Considering the above results, the Turbine network dynamics tool and the perturbation centrality measure may have a number of highly interesting future applications. Studies on perturbations of various real world networks were used to assess network robustness [48]. Perturbation analysis was used in the identification of drug target candidates, including multi-target drugs or allo-network drugs [11], [49]–[52]. Sequential perturbations have been suggested as a key modality of anti-cancer therapies [48]. Input signals with different dynamical profiles cause several non-trivial phenomena in signaling networks, such as kinetic insulation [53]. All these possibilities may be assessed by Turbine in the future and can be extended to ecosystems, social networks (infection spread, viral marketing) and engineered networks (power grids, Internet, etc.).

In conclusion, here we introduced Turbine, a new method for the analysis of network dynamics, and used it to study the propagation of perturbations in modular benchmark graphs and several types of real-world networks. We applied a dynamic model based on the concept of communicating vessels, and defined a new measure of dynamic network centrality, called as perturbation centrality. Hubs, inter-modular bridges and signal transducing amino acids were identified as nodes of high perturbation centrality, and were in agreement with a large number of earlier data. Changes of perturbation centrality in stressed yeast interactomes well described known functional changes after stress. The Turbine method and perturbation centrality open a large variety of options for future studies on network robustness, signaling mechanisms, drug design, as well as management of ecosystems, social and engineered networks.

## Methods

### Brief description of the Turbine program

An in-house designed program package, Turbine (http://turbine.linkgroup.hu) was used for the simulation of network perturbations. Turbine is a MATLAB and R-compatible toolkit for the analysis of network dynamics (including perturbations). Turbine contains multiple sub-programs written in C++, and a viewer written in C# to enable visual interpretation of the results. The program is using its own binary data and network format for performance reasons, but converters from multiple file formats, such as the *Pajek* network format or the *MATLAB/Octave* data format are also part of the default toolkit.

Turbine is based on a generalization of the systems theory approach [54] to complex networks. In the program we assign a state variable to all parameters of a network, which are expected to change during the simulation. Every node or edge has a separate value of every defined state variable. The effects of the state variables on each other (the evolution of the system in time) are determined by the particular *network dynamics model* used. In systems theory, this model is a set of ordinary or partial differential equations describing the change of the state variables in time, taking into account the effects of other state variables on the current component. In Turbine, any algorithm can be used as a model, making the user capable of simulating virtually any dynamics in any network. In the model, the user has to define the values of the state variables for the next step based on the values in the current step, which translates to creating a C++ function named PerStep(), which should return the values of the state vector for the (n+1)^th^ time step given all preceding values in the 1^st^ through n^th^ time steps. Of course, a model file may opt not to use all this information, and indeed, the communicating vessels model only uses the state vector of the previous, n^th^ step, as it will be described in the difference form of the model equations of the communicating vessels model described in the following section. Turbine models are stored as extendable and replaceable DLL files. Multiple default models – such as “ripple” for testing wave-like propagation, “gossip” for testing binary probabilistic information spreading, and “XY” modeling the evolution of the Prisoner's Dilemma game in a network – are supplied with the Turbine program package available at our website: http://turbine.linkgroup.hu. Selecting a model for a given network requires background knowledge on the nature of the dynamics of the complex system represented by the network. In the future, we plan to introduce more specialized dynamics such as “integrate-and-fire” for neural networks to make model selection simpler.

For running a simulation, the user has to define the 1.) time of the individual steps (called as step-time); 2.) the total analysis time, which together with the step-time determines the number of performed iterations, and 3.) the starting values of the state variables. It is also possible to introduce perturbations to the system during the simulation. A perturbation can be applied to every state variable, separately for each node or edge, and the value corresponding to the perturbation at the current time step is added to the current value of the node's or edge's instance of the state variable. This way, any combination of node and edge perturbations can be added to the system (e.g. single, multiple, sequential or continuous), at any time, which allows the user to test the response of the network to any environmental effects, such as different drugs or drug-combinations, or any intrinsically generated effects, such as gene expression noise. The detailed description of the Turbine program, its User Guide and freely downloadable versions of Turbine, as well as their source codes are available at our web-site: http://turbine.linkgroup.hu.

### The communicating vessels model

In the simulations of this paper, a model with only one state variable (energy) was used, to assess the dissipation of information (e.g. physical perturbations) in complex networks. The basic idea behind the model was that intensive physical variables (e.g. temperature) tend to perform an equalization-like dynamics behaving like communicating vessels. In the communicating vessels model network nodes represent the vessels and edges represent their connecting pipes. The algorithm of the model is as follows: in each time-step, every node transfers a proportion of its available energy through every available edge, proportional to 1.) the duration of the time-step; 2.) the weight of the edge (corresponding to ‘pipe diameter’); and 3.) the difference of the state variables on the two ends of the edge (corresponding to ‘pipe pressure’). There was a ‘vaporization’ effect, meaning that a constant amount of the available energy of every node could be dissipated in every step, which is an important property of most dynamical systems including molecular networks. Based on the above considerations, the differential form of the equation of the communicating vessel model is the following:
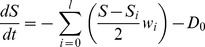
where ***S*** is the value of the state variable (energy) of the starting node of the edge, ***S_i_*** is the state variable (energy) of the node on the other end of the current edge, ***w_i_*** is the weight of the current edge, ***l*** is the number of edges (degree) of the current node, and ***D_0_*** is the dissipation coefficient, which is kept constant for all nodes.

The differential form is only equal to the discrete difference equations which the algorithm uses if the step-time is infinitesimal. However, analyzing differential equations are often much easier, and this form is more familiar for systems theorists. For the sake of completeness, we have also included the difference equation form calculated by the algorithm below:
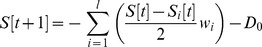



Variable names match the ones of the differential form: ***S*** is the value of the state variable (energy) of the starting node of the edge, ***S_i_*** is the state variable (energy) of the node on the other end of the current edge, ***w_i_*** is the weight of the current edge, ***l*** is the number of edges (degree) of the current node, and ***D_0_*** is the dissipation coefficient, which is kept constant for all nodes. This difference form of the equation shows an important criterion when choosing edge weights: the (absolute value of the) sum of weighted out-degrees should not exceed the reciprocal of the step-time (1/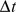) for any node, otherwise more energy will be propagated outwards than the amount contained in the node which – besides violating the conservation of energy – can destabilize the whole simulation. We have created a plugin for Turbine (*normalize mflow*) which can normalize any network to meet this criteria, keeping *relative* edge weights intact. This criterion can be summarized in the following equation:
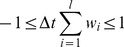



The described equations (and the algorithm) of the communicating vessels model can be naturally extended to directed graphs. This modification can be attained by separating the energy transfer on the inbound and outbound edges, like the following:
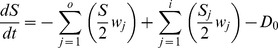
where ***S*** is the value of the state variable (energy) of the current node, ***o*** is out-degree of the node, ***w_j_*** is the weight of the current edge, ***S_j_*** is the state variable (energy) of the current neighbor, ***i*** is the in-degree of the current node, and ***D_0_*** is the (constant) dissipation coefficient. This model also allows the assessment of information propagation, silencing times and perturbation centrality in directed real-world networks such as the Internet, citation networks, or biological signaling networks.

This model provides a good starting point for the simulation of most network dynamics, if more detailed information is not available about the mechanism of the actual dynamic process. The DLL file containing the communicating vessels model is included with all Turbine packages, and is available on our web-site: http://turbine.linkgroup.hu.

### Turbine simulations

Scripts for running all simulations with the exact parameters and networks used are downloadable from our web-site: http://turbine.linkgroup.hu.

### Calculation of fill time, silencing time and perturbation centrality

Two types of tests were conducted on the target networks using the communicating vessels model described above. In the calculation of fill time one node was excited with 10,000 units of energy *in each time step*, and a D_0_ = 0 constant dissipation was set. The speed of the propagation of the perturbation starting at the given node was characterized by the fill time of the network, which was defined as the time during the simulation when more than 80% of the nodes in the network had an energy value larger than 1. The fill time measure was calculated for each node of the network.

In the calculation of silencing time and perturbation centrality one node was excited with a given amount of energy *at the start of the simulation*, which was 10,000, 40,000 or 1,000,000 units as stated in the individual simulations, and a D_0_ = 1 constant dissipation was set. Silencing time was defined as the first time, when all of the nodes had an energy value less than a pre-set threshold, which was 1 in all of our experiments. The speed of the dissipation of the perturbation starting at the given node was characterized by the perturbation centrality, defined as the reciprocal of the silencing time of an n*10 sized Dirac-delta-type perturbation, where *n* is the number of nodes in the network, with the dissipation and the threshold for the silencing time set to 1 (the reasons of this choice can be found in **Table S2** of **Text S1**). All measures were calculated for each node of the network.

Turbine plug-ins to calculate the silencing time, the fill time, a script to calculate the perturbation centrality measure, as well as the User Guide can be downloaded from the web-site: http://turbine.linkgroup.hu.

### Generating protein structure networks from structure information

Protein structure networks from the substrate-free and substrate-bound form of methionyl-tRNA synthetase enzyme (Met tRNA synthase [43]), as well as the substrate-free (1PO5, [55]) and imidazole-bound (1SUO, [56]) form of rabbit cytochrome P450 2B4 were built with the RINerator software [5], using the PDB files received from the authors of [43] in the case of MetRS, and using the published 1PO5 and 1SUO structures from the Protein Data Bank. The absolute value of interaction strengths was used for network building, since the companion program of RINerator called Probe returns negative interaction strengths for repulsive interactions, but our perturbation propagation model is assumed to only depend on the strength of the interaction rather than its repulsive or attractive nature.

### Characterization of proteins important in perturbation propagation in resting and stressed yeast cells

For the functional characterization of proteins having the highest perturbation centralities (or highest changes in perturbation centralities) in resting yeast cells or yeast cells after various types of stresses, term enrichment analysis was performed using the R plugin of the g:Profiler [38] web service. g:Profiler uses terms from Gene Ontology, KEGG, and several pathway databases. Significant enrichment was stated when the p-value of a term was strictly less than 0.05 after applying Bonferroni correction for multiple testing.

### Statistical methods

Statistical analyses including the calculation of means, medians, standard deviations, Welch two-sample t-tests, Wilcoxon rank-sum tests and correlation analyses were done using the R package [57].

## Supporting Information

Text S1 **This supporting information (Text S1) contains 10 Supplementary Figures, 9 Supplementary Tables, Supplementary Results, Supplementary Methods as well as 39 Supplementary References.**
(PDF)
