# Supplementary material for: Perturbation Centrality and Turbine: A Novel Centrality Measure Obtained Using a Versatile Network Dynamics Tool
Source: PLoS One. 2013 Oct 21;8(10):e78059. doi: 10.1371/journal.pone.0078059 (PMC3804472; doi:10.1371/journal.pone.0078059)
Supplement: Text S1 — This supporting information (Text S1) contains 10 Supplementary Figures, 9 Supplementary Tables, Supplementary Results, Supplementary Methods as well as 39 Supplementary References. (PDF) [file pone.0078059.s001.pdf]

Supplementary Information for

Perturbation centrality and Turbine: a novel centrality measure obtained using a versatile network dynamics tool

Kristóf Z. Szalay and Peter Csermely[1]
Department of Medical Chemistry, Semmelweis University, Budapest, Hungary

See the supporting website for further information: http://turbine.linkgroup.hu

Table of contents

Supplementary Figures **3**
Figure S1. Average perturbation centrality and "number of visited nodes" values plotted against the ratio of inter-modular edges. 3
Figure S2. Correlation of perturbation centrality, "number of visited nodes" and node degree. 4
Figure S1. Comparison of perturbation centrality with the "number of visited nodes" measure of ITM-Probe as a function of node degree with different ratios of inter-modular edges of benchmark graphs. 5
Figure S4. A visual representation of the relation among different centrality measures. 6
Figure S5. Distribution of perturbation centralities in 10 benchmark and real-world networks. 7
Figure S6. Average perturbation centralities for different sets of residues in protein structure networks. 9
Figure S7. Average betweenness centralities of different residue groups in Met-tRNA synthetase and cytochrome P450 enzymes. 11
Figure S8. Average closeness centralities of different residue groups in Met-tRNA synthetase and cytochrome P450 enzymes. 13
Figure S9. Amino acids of Met-tRNA synthetase directly bound to $tRNA^{Met}$ 14
Figure S10. Results for edgetic perturbations. 15
Supplementary Tables **16**
Table S1. Correlation between the reciprocal of fill time and closeness centrality 16
Table S2. Correlation of silencing times calculated for three different-sized starting perturbations in 8 real-world and two benchmark networks 17
Table S3. Statistically significantly enriched terms in the top 100 protein set of the DIP yeast interactome (release 2005) [7] containing proteins with *largest absolute* perturbation centrality in differently stressed cases. 18
Table S4. Statistically significantly enriched terms in the top 100 protein set of the DIP yeast interactome (release 2005) [7] containing proteins with *largest increase* of perturbation centrality in differently stressed cases. 22
Table S5. Most significantly enriched terms in the top 100 protein set of the DIP yeast interactome (release 2005) [7] containing proteins with *largest decrease* of perturbation centrality in differently stressed cases 27
Table S6. Run-time and memory usage of the Turbine program during simulation of different synthetic networks 35
Table S7. Details of the used realizations of modular benchmark graphs 36
Table S8: Average perturbation centrality of inter-modular nodes and inter-modular hubs as compared to the average perturbation centrality of intra-modular, non-hub nodes 38

[1]Corresponding author: Csermely, P. (csermely.peter@med.semmelweis-univ.hu)

Table S9. Signaling residues and residues of experimentally verified importance in Met-tRNA synthetase protein structure network 39
Supplementary Results **41**
Connection of the communicating vessels model with perturbation dissipation using shortest paths and having an exponential decay 41
Comparison of the communicating vessels model with the random walk model of ITM-Probe 42
Dissipation-free propagation of perturbation is characterized by fill time, which reciprocally correlates with closeness centrality 43
Comparing the effect of degree and modular position on perturbation centrality in real-world networks 44
Amino acids participating in intra-protein signaling have a high perturbation centrality in the protein structure network 44
Testing the effects of edgetic perturbations 45
Supplementary Methods **46**
Description of the networks used 46
Benchmark graphs 46
Protein structure networks 46
Yeast protein-protein interaction networks 47
Metabolic networks 48
School friendship network 48
Network measures and their calculation 49
Network modularization 49
Determination of the “number of visited nodes” measure of the ITM-Probe method 49
Supplementary References **50**

Supplementary Figures

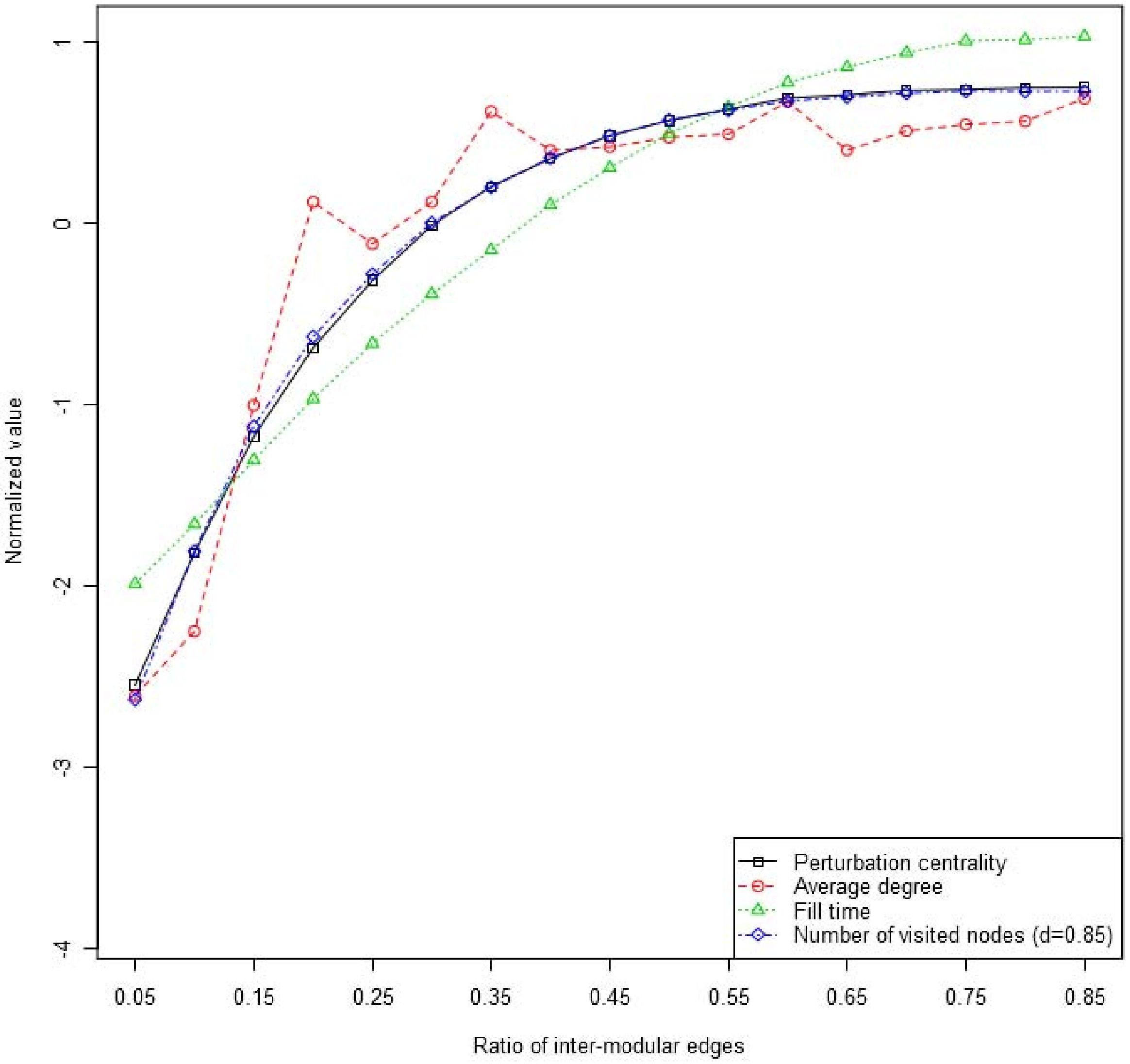

Figure S1. Average perturbation centrality and "number of visited nodes" values plotted against the ratio of inter-modular edges.

Scale-free, modular benchmark graphs [1] were generated as described in Supplementary Methods. Average perturbation centrality (black squares), average degree (red circles), the reciprocal of average fill time (green triangles, added perturbation: 10,000 units per step) and average "number of visited nodes" (blue diamonds) for a damping value of 0.85 were calculated from 3 randomly generated benchmark graphs with ratios of inter-modular edges ranging from 0.05 to 0.85 with steps of 0.05 as described in Methods of the main text for Turbine (with the change that a 95% threshold was used for the fill time – that is, 95% of the network had to have an energy value larger than 1 – since the benchmark networks were much more homogeneous than real-world networks) and in the Supplementary Methods for ITM-Probe [2]. Values were normalized using the scale function of the R package [3]. Note that the Spearman correlation between the various values was more than 0.95, except for the average degree.

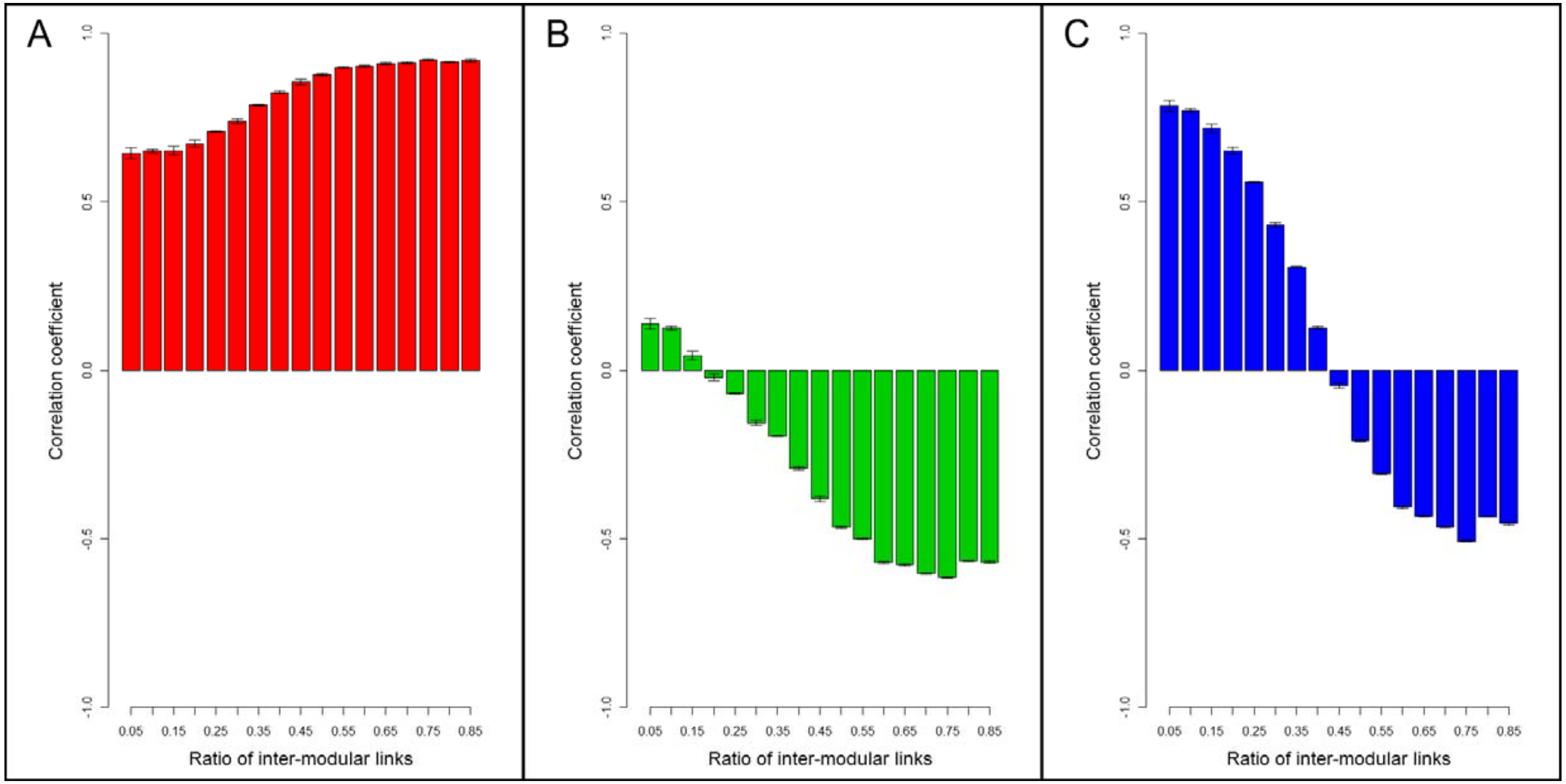

Figure S2. Correlation of perturbation centrality, "number of visited nodes" and node degree.

Scale-free, modular benchmark graphs [1] were generated as described in Supplementary Methods. Perturbation centrality and "number of visited nodes" measures were calculated from 3 sets of randomly generated benchmark graphs with ratios of inter-modular edges ranging from 0.05 to 0.85 with steps of 0.05 as described in Methods of the main text and in Supplementary Methods for ITM-Probe [2], respectively. Spearman correlations were calculated using the R package [3]. Panel A shows correlation of perturbation centrality versus node degree, Panel B shows the correlation of number of visited nodes versus the node degree, and Panel C shows the correlation of perturbation centrality and number of visited nodes. The data in Panel A reinforces the observation in the main text that the degree becomes more important in the determination of the silencing time as the modules become more and more fuzzy and overlapping. Interestingly, results from ITM-Probe behave in an exactly opposite way: as the communities become more overlapping, the number of visited nodes measure quickly becomes negatively correlated with the degree (possibly because random walks can "turn back"). These two effects taken together resulted in a large correlation between the perturbation centrality and number of visited nodes when there were pronounced modules, and a negative correlation when the modules became fuzzier, which is shown by the data in Panel C.

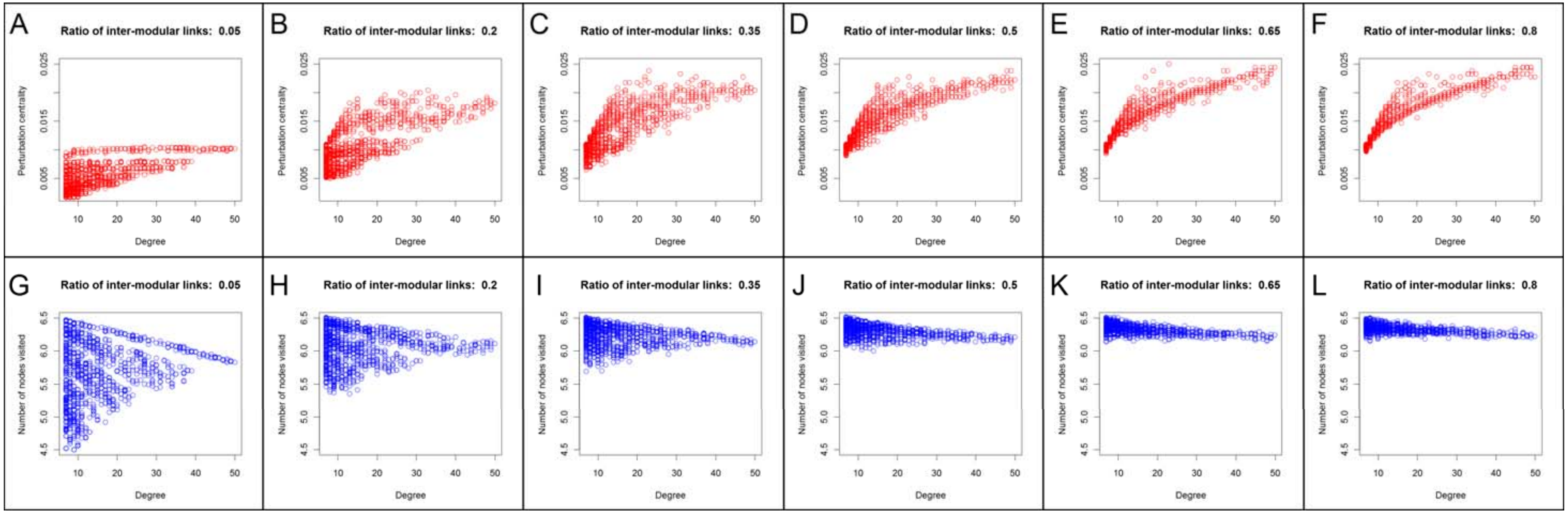

Figure S1. Comparison of perturbation centrality with the "number of visited nodes" measure of ITM-Probe as a function of node degree with different ratios of inter-modular edges of benchmark graphs.

Scale-free benchmark graphs [1] with overlapping modules were generated as described in Supplementary Methods. Perturbation centrality (red, Panels A through F) and "number of visited nodes" measures (blue, Panels G through L) were calculated as described in Methods of the main text and in Supplementary Methods for ITM-Probe [2], respectively. For the generation of the benchmark graphs with ratios of inter-modular edges 0.05, 0,2, 0.35, 0.5, 0.65 and 0.80 appearing on Panels A/G, B/H, C/I, D/J, E/K and F/L, respectively, a random seed of 87 was used. The results suggest that nodes in the networks with pronounced modules give similar results using Turbine and ITM-Probe (observe the same striping pattern showing the better perturbation propagation capability of nodes having inter-modular edges). On the contrary, in networks with fuzzy modules, the result is still correlated with the degree in Turbine, but ITM-Probe results do not seem to depend on the degree. These are the same results that can be obtained from Figure S2; this figure serves as an illustration of the possible underlying pattern behind the change in correlation: the number of nodes visited measure seems to have an upper saturation-like limit in ITM-Probe in networks with largely overlapping modules.

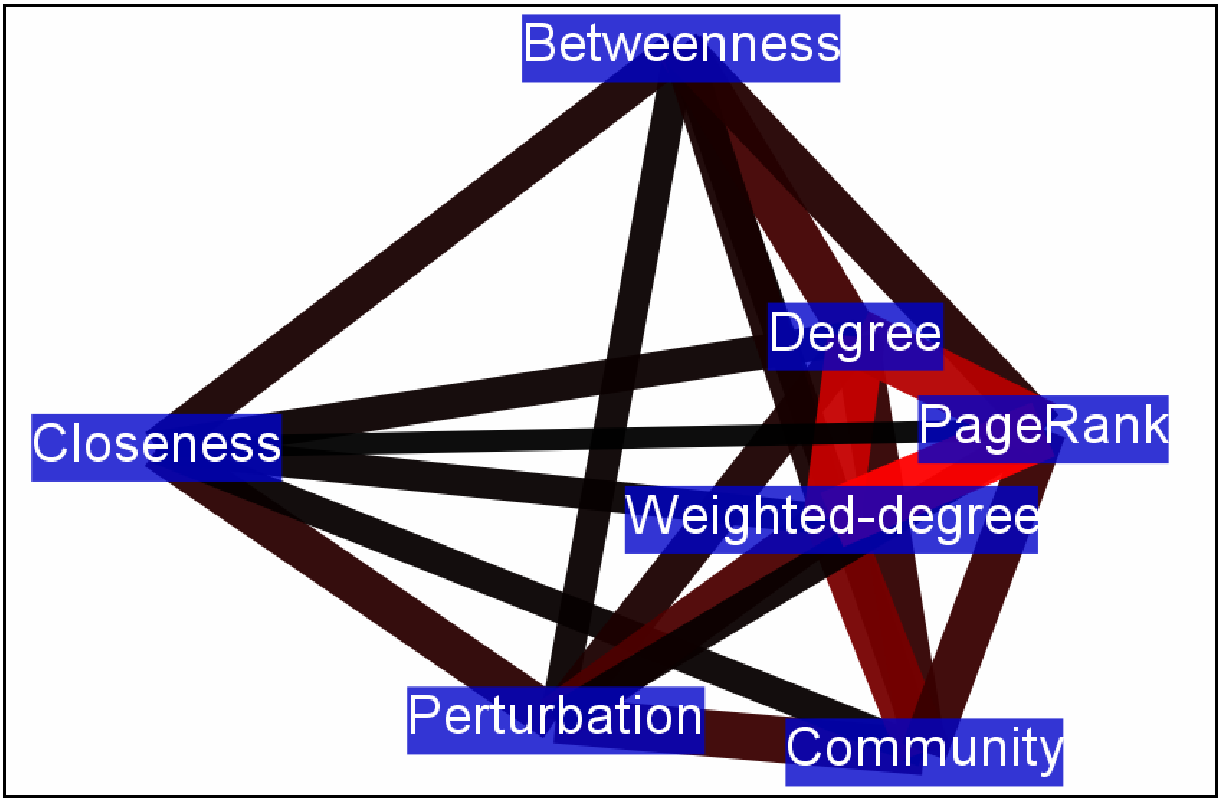

Figure S4. A visual representation of the relation among different centrality measures.

We calculated Spearman correlations between different centrality measures for the 10 benchmark and real-world networks shown in Table 2 of the main text. A 7-node graph was created from this data using the different centrality measures as nodes, and the average correlation between pairs of centralities as edge weights. The graph was thereafter laid out using the ForceAtlas 2 layout algorithm (which uses edge weights) of Gephi [4] with default settings except for the “Edge weight influence” option, which was set to 5.0. The layout generated this way can be a good approximation of the relations between different centralities, since the more correlated centrality measures are connected by more powerful “springs”; thereby their final position is closer to each other. It is visible on the figure that the perturbation centrality measure occupies a new position with largest correlations to closeness centrality, community centrality and weighted degree, just as the mean correlations of Table 2 in the main text show.

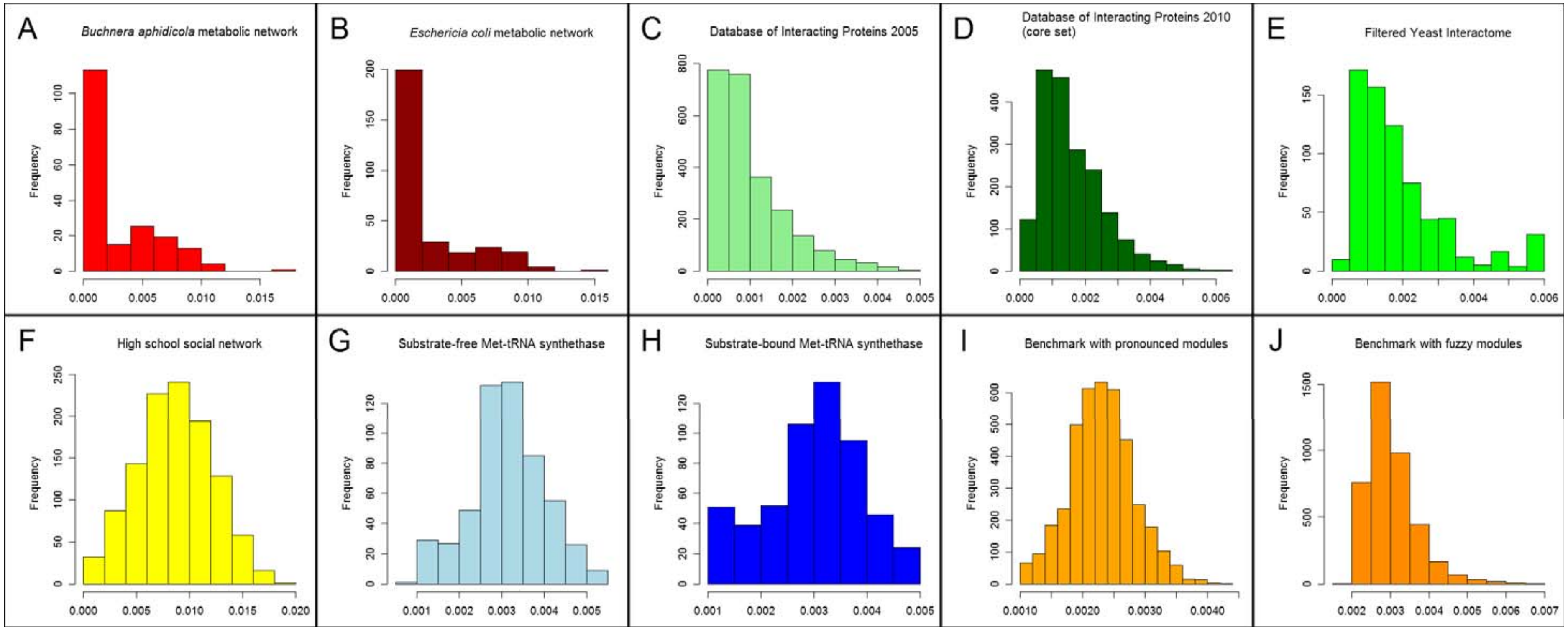

Figure S5. Distribution of perturbation centralities in 10 benchmark and real-world networks.

Histograms were generated using the same data set for 2 benchmark and 8 real-world networks that were used for **Table S1** and **S2**. Detailed descriptions of the networks are available in **Supplementary Methods**. Perturbation centralities were calculated according to **Methods** of the main text. Histograms from the perturbation centralities were generated using the "hist" command of R [3], with default settings. The tested social network (Panel F), the modular benchmark network [1] with pronounced modules (Panel I) and both conformations of Met-tRNA synthetase [5] (Panels G and H) had approximately normal distributions of perturbation centrality values. The Filtered Yeast Interactome [6] (Panel E) and the 2010 release of the Database of Interacting Proteins [7] (Panel D), as well as the modular benchmark network with fuzzy modules (Panel J) seemed to have approximately lognormal distributions for perturbation centrality. The histogram of the 2005 release of the Database of Interacting Proteins looked like a scale-free distribution (Panel C), and finally, the most skewed distributions were the perturbation centralities of the two metabolic networks [8,9], which looked like exponential distributions. However, all distributions failed the Shapiro-Wilk normality test ($p=5*10^{-16}$, $5*10^{-22}$, 10^{-44}, 10^{-31}, $3*10^{-26}$, 0.0003, 0.0094, $2*10^{-7}$, $3*10^{-9}$, $3*10^{-45}$, respectively), so the Wilcoxon rank-sum test had to be used for statistical significance analysis instead of a t-test.

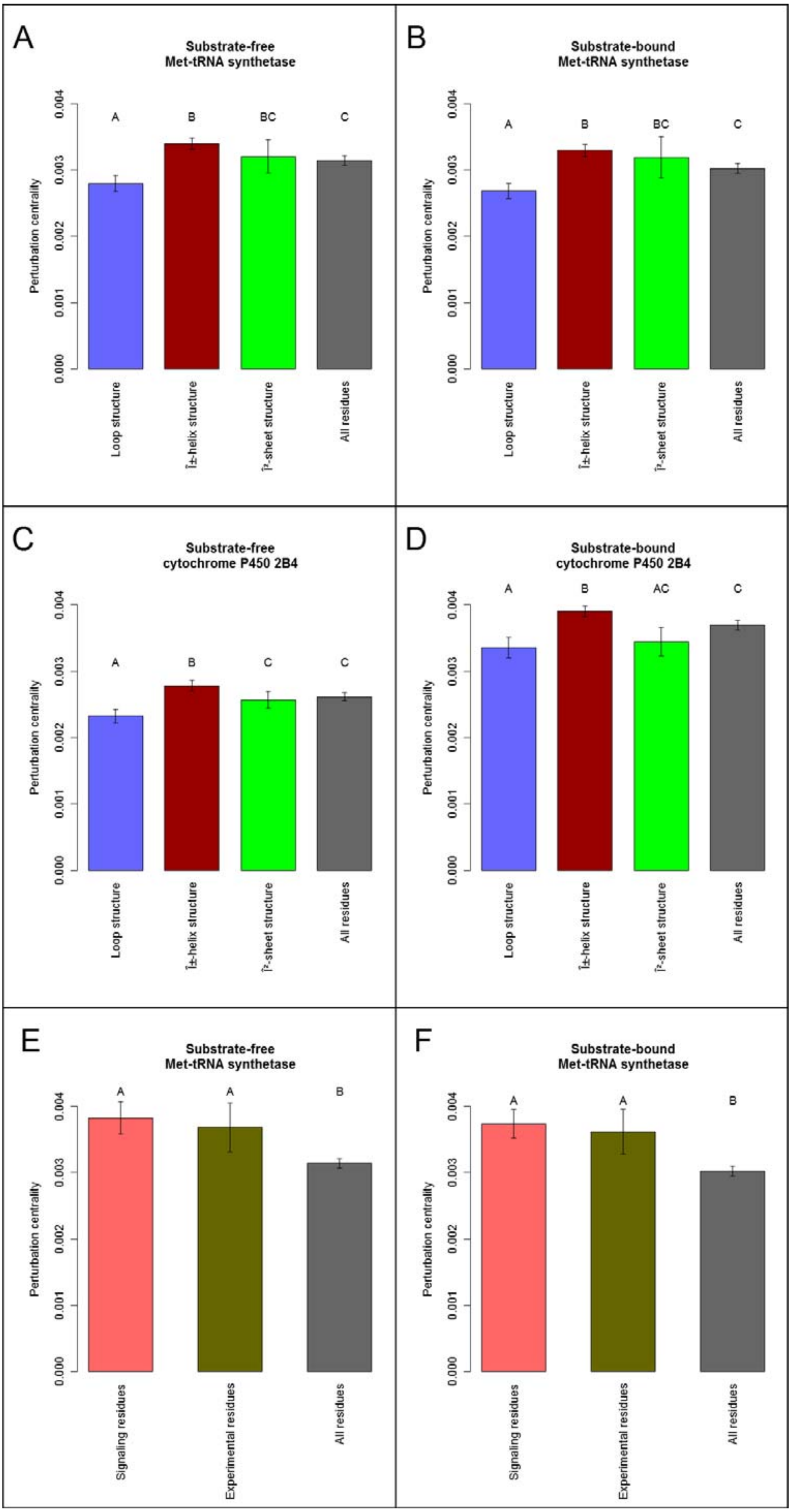

A
Substrate-free
Met-tRNA synthetase
B
Substrate-bound
Met-tRNA synthetase
C
Substrate-free
cytochrome P450 2B4
D
Substrate-bound
cytochrome P450 2B4
E
Substrate-free
Met-tRNA synthetase
F
Substrate-bound
Met-tRNA synthetase
Perturbation centrality
0.000
0.001
0.002
0.003
0.004
Loop structure
Î±-helix structure
Î²-sheet structure
All residues
Signaling residues
Experimental residues
A
B
BC
C
AC

Figure S6. Average perturbation centralities for different sets of residues in protein structure networks.

Protein structure networks of the substrate-free and substrate-bound forms of the *E. coli* Met-tRNA synthetase and rabbit cytochrome P450 2B4 proteins were generated as described in **Methods** and **Supplementary Methods** of **Text S1**. Assignment of secondary structures for different amino acids was done by PyMOL. Error bars show standard error of the mean. Different letters on top of the bars mean significantly different groups (α=0.01, Wilcoxon rank-sum test). Panels A and B show data calculated for the substrate-free and substrate (Met-AMP/$tRNA^{Met}$)-bound form of Met-tRNA synthetase, respectively. Panels C and D show data calculated for the substrate-free and substrate (imidazole)-bound form of cytochrome P450 2B4. In all cases amino acids of loops had significantly ($p=3.2*10^{-6}$, $9.5*10^{-6}$, $2.3*10^{-6}$, 0.0001 for the free and bound conformations of Met-tRNA synthetase and cytochrome P450, respectively; Wilcoxon rank-sum test, α=0.00625 adjusted with Bonferroni correction) lower perturbation centrality than average, while α-helices had significantly higher (p=0.00023, 0.00015, 0.00083 ,0.0014 for the free and bound conformations of Met-tRNA synthetase and cytochrome P450, respectively; Wilcoxon rank-sum test, α=0.00625 adjusted with Bonferroni correction) than average perturbation centrality. Panels E and F show the average perturbation centralities of amino acids belonging to intra-protein communication pathways predicted by Ghosh and Vishveshwara [5] ("Signaling residues"), as well as amino acids with experimentally verified importance [5] ("Experimental residues"). Light blue, dark red and green bars show average perturbation centralities of amino acids in loop, α-helical and β-sheet structures, while gray bars show the global average perturbation centrality calculated for the whole protein. Pink and brown bars of Panels E and F show average perturbation centralities of the Signaling and Experimental residues, respectively. Note that both betweenness and closeness centralities were less successful than perturbation centrality in differentiating between the above amino acid sets (cf. the current Figure with **Figures S7** and **S8** of **Text S1**).

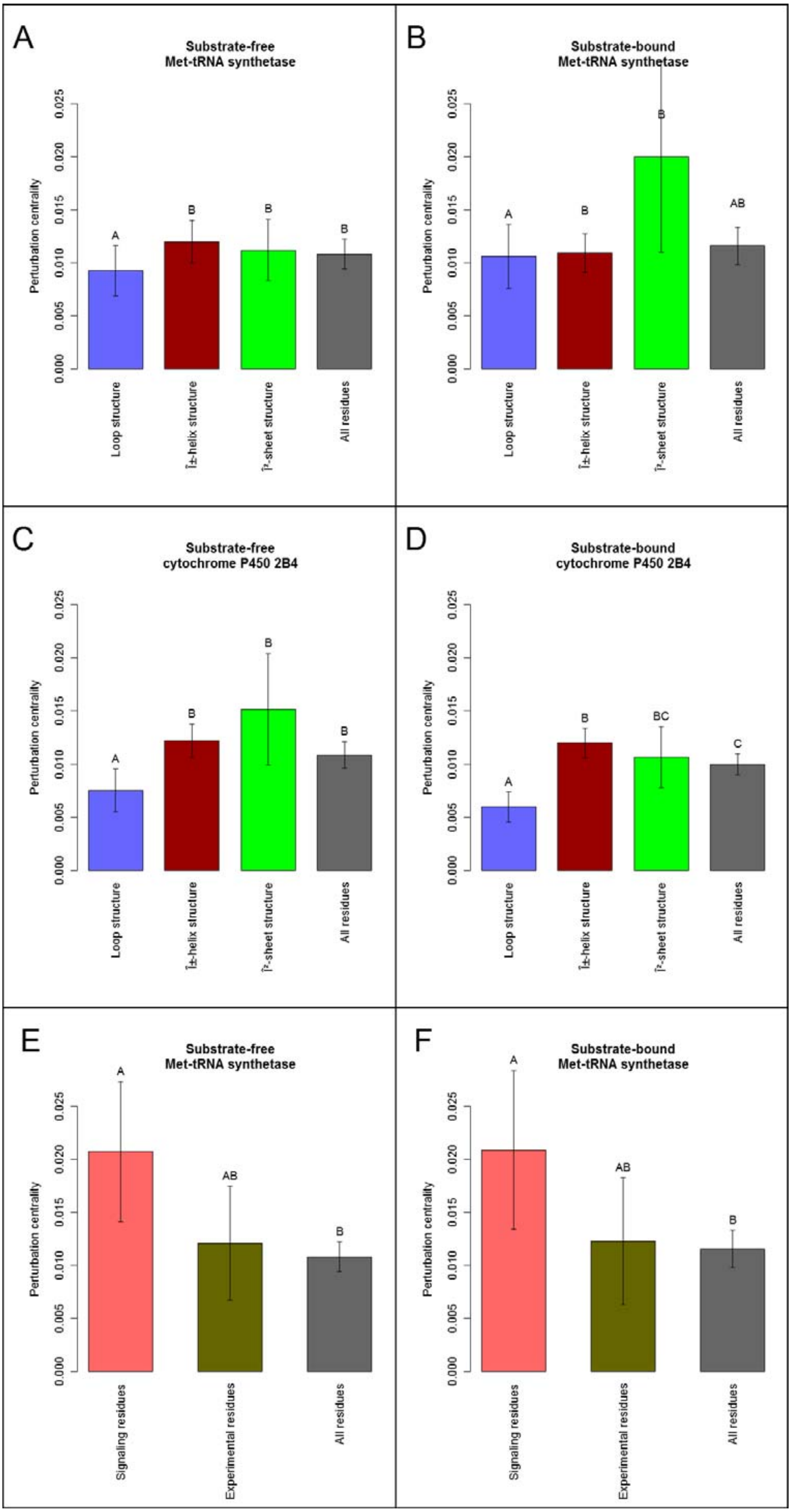
A
Substrate-free
Met-tRNA synthetase
B
Substrate-bound
Met-tRNA synthetase
C
Substrate-free
cytochrome P450 2B4
D
Substrate-bound
cytochrome P450 2B4
E
Substrate-free
Met-tRNA synthetase
F
Substrate-bound
Met-tRNA synthetase
Perturbation centrality
0.000
0.005
0.010
0.015
0.020
0.025
Loop structure
Î±-helix structure
Î²-sheet structure
All residues
Signaling residues
Experimental residues
A
B
AB
BC
C

Figure S7. Average betweenness centralities of different residue groups in Met-tRNA synthetase and cytochrome P450 enzymes.

This figure is a direct parallel to **Figure S6** and **Figure S8**, here using *betweenness* centrality instead of perturbation and closeness centralities, respectively. Protein structure networks of the substrate-free and substrate-bound forms of the *E. coli* Met-tRNA synthetase and rabbit cytochrome P450 2B4 proteins were generated as described in **Methods** of the main text and **Supplementary Methods**. Assignment of secondary structures for different amino acids was performed by PyMOL. Error bars show standard error of the mean. Different letters mean significantly different groups (α=0.01, Wilcoxon rank-sum test). Panels A and B show data calculated for the substrate-free and substrate-bound form of Met-tRNA synthetase, respectively. Panels C and D show data calculated for the substrate-free and imidazole-bound forms of cytochrome P450 2B4. Panels E and F show the average perturbation centralities of predicted communication pathways as described by Ghosh and Vishveshwara [5] ("Signaling residues") and other residues with experimentally verified importance [5] ("Experimental residues"). Light blue, dark red and green bars on Panels A through D show average perturbation centralities for residues in loops, α-helices and β-sheets, while gray bars show the global average perturbation centrality calculated for the whole protein. Pink bars on Panels E and F show average perturbation centralities of the Signaling residues, and brown bars show the means of the Experimental residues of Met-tRNA synthetase. Betweenness centrality returned by far the largest deviations of the three tested centralities (i.e. closeness, betweenness and perturbation centralities). Loops still had significantly (p=0.0016, 0.011, $4.3*10^{-5}$, $6.8*10^{-7}$ for the free and bound conformations of Met-tRNA synthetase and cytochrome P450, respectively; Wilcoxon rank-sum test, α=0.00625 adjusted with Bonferroni correction) lower mean centrality values than the global average in all networks (but the substrate-bound Met-tRNA synthetase). Using betweenness centrality α-helices can no longer be distinguished from the global mean (except for the substrate-bound form of cytochrome P450). Signaling residues could still be distinguished from the global mean (Panels E and F), but the differences in centralities for the Experimental residues were no longer significant.

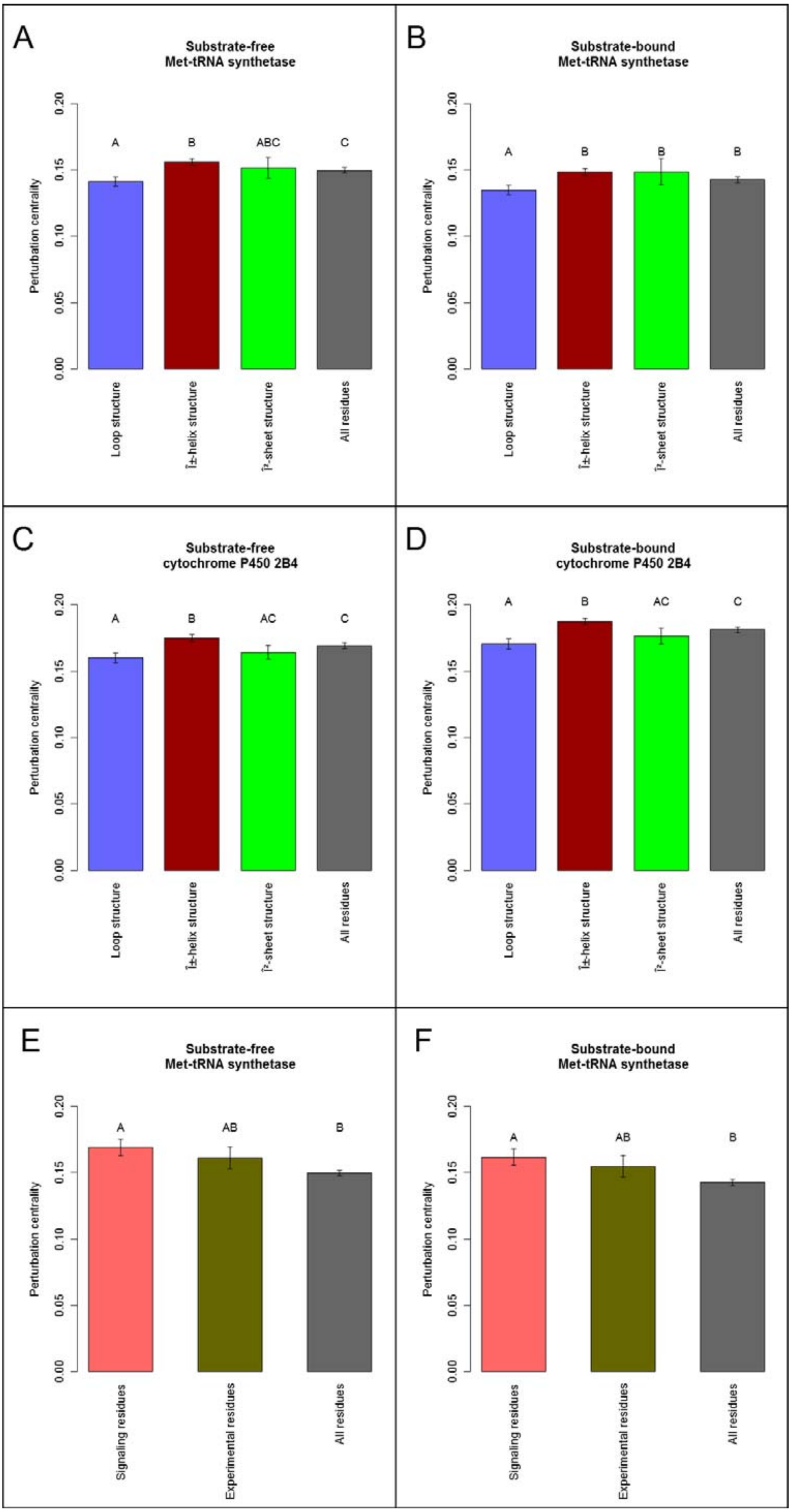
A
Substrate-free
Met-tRNA synthetase
Perturbation centrality
0.00
0.05
0.10
0.15
0.20
A
B
ABC
C
Loop structure
Î±-helix structure
Î²-sheet structure
All residues
B
Substrate-bound
Met-tRNA synthetase
C
Substrate-free
cytochrome P450 2B4
AC
D
Substrate-bound
cytochrome P450 2B4
E
Substrate-free
Met-tRNA synthetase
AB
Signaling residues
Experimental residues
F
Substrate-bound
Met-tRNA synthetase

Figure S8. Average closeness centralities of different residue groups in Met-tRNA synthetase and cytochrome P450 enzymes.

This figure is a direct parallel to **Figure S6** and **Figure S7**, here using *closeness* centrality instead of perturbation or betweenness centralities, respectively. Protein structure networks of the substrate-free and substrate-bound forms of the *E. coli* Met-tRNA synthetase and rabbit cytochrome P450 2B4 proteins were generated as described in **Methods** of the main text and **Supplementary Methods**. Assignment of secondary structures for different amino acids was performed by PyMOL. Error bars show standard error of the mean. Different letters mean significantly different groups ($\alpha=0.01$, Wilcoxon rank-sum test). Panels A and B show data calculated for the substrate-free and substrate-bound form of Met-tRNA synthetase, respectively. Panels C and D show data calculated for the substrate-free and imidazole-bound form of cytochrome P450 2B4. Panels E and F show the average perturbation centralities of predicted communication pathways as described by Ghosh and Vishveshwara [5] ("Signaling residues") and other residues with experimentally verified importance [5] ("Experimental residues"). Light blue, dark red and green bars on Panels A through D show average perturbation centralities for residues in loops, α-helices and β-sheets, while gray bars show the global average perturbation centrality calculated for the whole protein. Pink bars on Panels E and F show average perturbation centralities of the Signaling residues, and brown bars show the means of the Experimental residues of Met-tRNA synthetase. Closeness centrality returned smaller deviations than perturbation centrality. Interestingly, the distinction power of closeness centrality was slightly lower in Met-tRNA synthetase, and exactly the same in cytochrome P450 as the distinction power of perturbation centrality. In particular, the Experimental residues could no longer be distinguished from the global mean (Panels E and F, $p=0.034$, $p=0.04$, respectively; 0.007 and 0.006 with perturbation centrality), α-helices did not have significantly higher mean closeness centrality than the global average in the substrate-bound Met-tRNA synthetase (Panel B, $p=0.014$ vs. 0.00015 with perturbation centrality), and β-sheets were no longer distinguishable from loops in the substrate-free Met-tRNA synthetase (Panel A, $p=0.035$ vs. 0.0034 with perturbation centrality). P-values were calculated using the Wilcoxon rank-sum test, $\alpha=0.0125$ adjusted with Bonferroni correction for a FWER of 0.05.

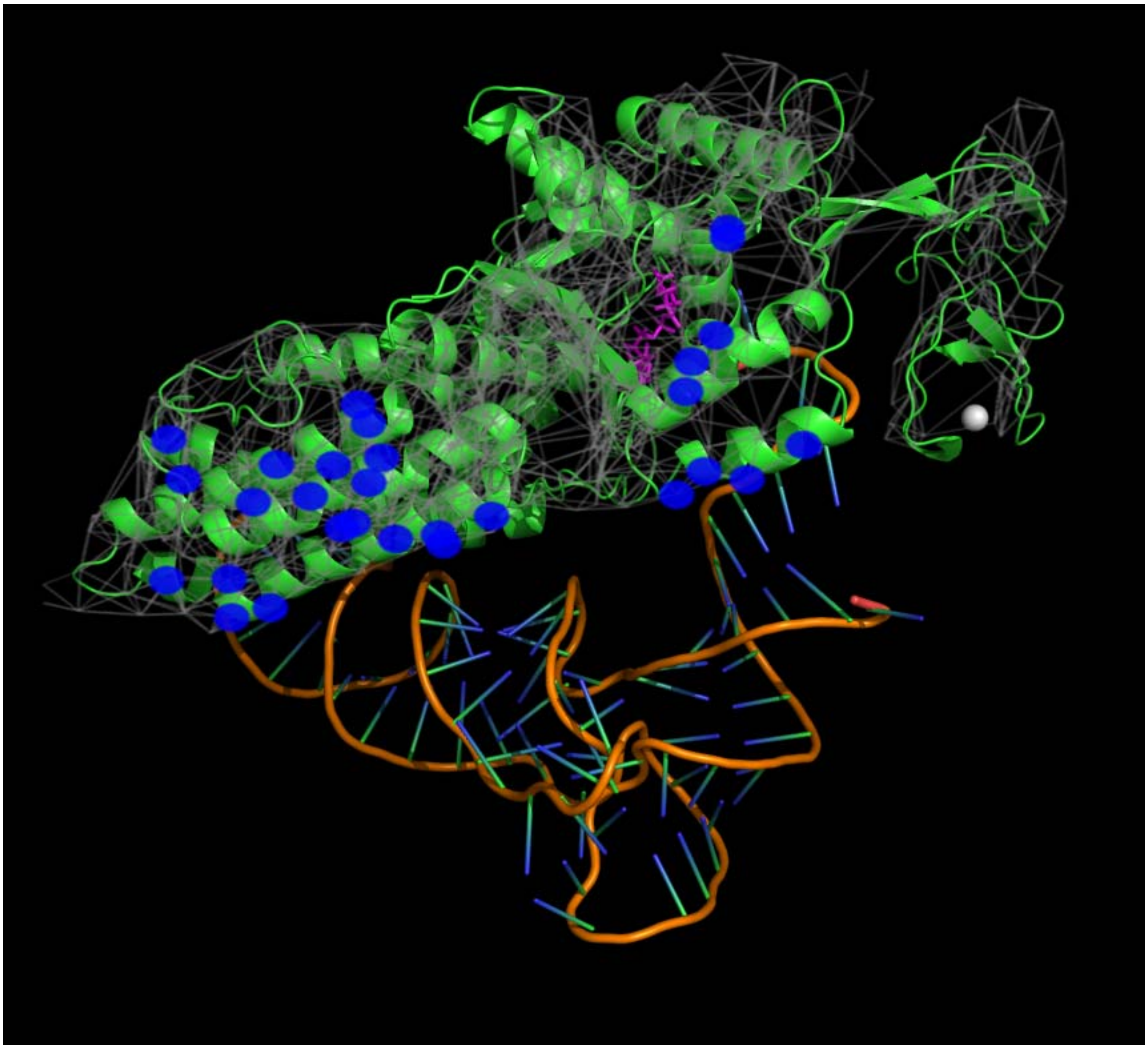

Figure S9. Amino acids of Met-tRNA synthetase directly bound to $tRNA^{Met}$.

The underlying protein structure network of Met-tRNA synthetase was calculated and visualized by the Turbine program as described in **Methods**, and was overlaid on the 3D image of the substrate-bound form of the protein (and its $tRNA^{Met}$ complex) generated with PyMOL [10] using ray-tracing. The bottom of the image shows the structure of $tRNA^{Met}$. The purple molecule in the middle of the protein structure is the substrate Met-AMP marking the active site of the enzyme, the white sphere on the right is the Zn^{2+} ion. Blue circles mark those amino acids, which are directly bound to the $tRNA^{Met}$, evidenced by an atomic distance of less than 4.5Å between any atom of the residue and the $tRNA^{Met}$, excluding hydrogen atoms.

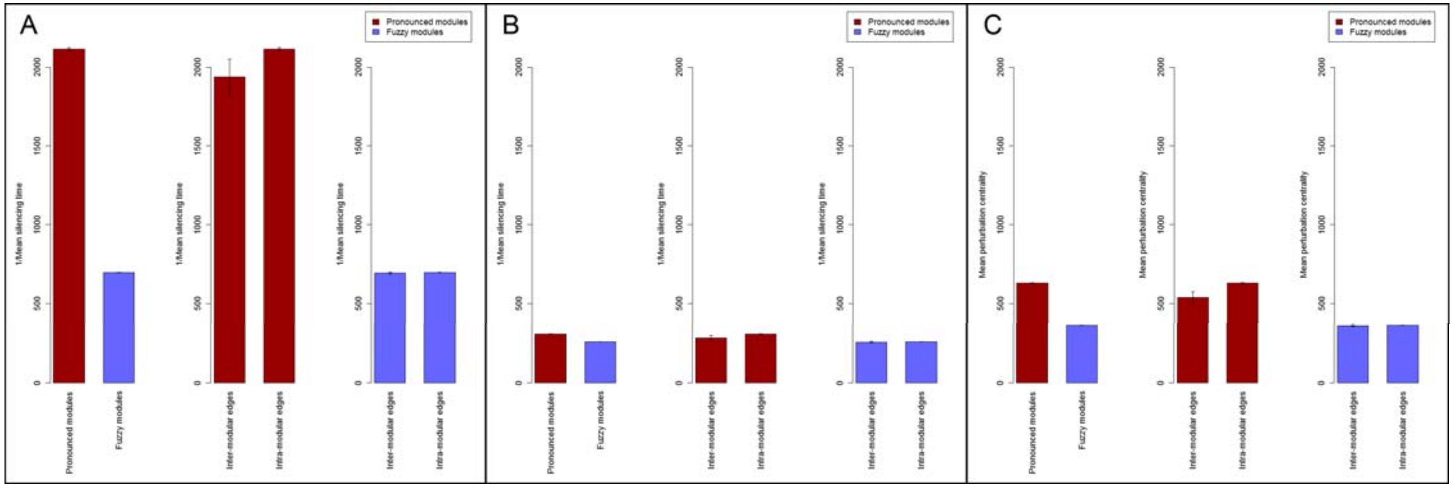

Figure S10. Results for edgetic perturbations.

We also tested the effects of edge-based perturbations as their importance have previously been stated in the literature [11]. Silencing time for an edge was calculated by propagating a given amount of energy (10,000, 40,000 and 1,000,000 units) from both end-nodes of an edge, simultaneously. We calculated silencing times for all edges for two modular benchmark networks [1], one with pronounced modules (only 5% of the links were inter-modular), and one with fuzzy modules (40% inter-modular links), both generated with the random seed 10. Silencing times were measured with a dissipation value of 1, and silencing threshold was also set to 1. Further definition of the silencing time is available in Methods of the main text. A link was termed inter-modular, if it was connecting two different communities. Dark red bars show data obtained on the network with pronounced modules, while light blue bars display data from the network with fuzzy modules. We could observe the very same effects for edge-based perturbations that we have observed for node-based perturbations. Panel A shows the mean perturbation centralities calculated for a large starting perturbation (1,000,000 units on both endpoints of an edge). There was a major ($p=0$, Wilcoxon rank-sum test) difference between the mean silencing times between fuzzy and pronounced modules in this case, and inter-modular edges were significantly ($p=0.0094$, $\alpha=0.025$ with Bonferroni correction, Wilcoxon rank-sum test) better spreaders of perturbation in the network with pronounced modules. The disparity between the edgetic perturbations of networks with fuzzy versus pronounced modules was nearly eliminated, if a starting perturbation of 10,000 units was applied (Panel B). These effects were the same as what we have demonstrated with node-based perturbations on Figures 1 and 2 of the main text. Panel C shows silencing times calculated for 40,000 units of starting energy corresponding to the definition of perturbation centrality measure. (40,000 units of starting energy was used, since the benchmark networks contained 4000 nodes, and perturbation centrality was defined as the reciprocal of the silencing time resulting from applying a perturbation of $10*n$ units of energy, where n is the number of nodes in the network, see **Methods** of the main text.) Data of Panel C verifies that the choice of $n*10$ units as a starting perturbation is a nice compromise between weighing the nodes' mesoscopic position, that is, the modular location (which can be detected using a large starting perturbation) and their local position, that is, the weighted degree of the node and its near neighbors (which can be detected using a small starting perturbation).

Supplementary Tables

Table S1. Correlation between the reciprocal of fill time and closeness centrality

**Networks**[a]	**Correlation**[b]	**Average fill time**[c]
Benchmark graphs with fuzzy modules	0.974	52
Benchmark graphs with pronounced modules	0.930	151
Substrate-free Met-tRNA synthetase protein structure network	0.957	76
Substrate-bound Met-tRNA synthetase protein structure network	0.941	94
Filtered Yeast Interactome	0.856	1344
Database of Interacting Proteins yeast interactome (release 2005)	0.857	215
Database of Interacting Proteins yeast interactome (release 2010)	0.941	168
B. aphidicola metabolic network	0.824	1225
E. coli metabolic network	0.746	1928
School friendship network	0.921	28
**Mean and standard error**	**0.895 (0.023)**	**528 (219.9)**

[a]Network descriptions are given in **Supplementary Methods** of **Text S1**.
[b]Correlation data show the Spearman's rho correlation of the reciprocal of the fill time versus the standard closeness centrality measure. Correlations between the reciprocal of fill time and closeness centrality are much stronger than those between perturbation centrality and closeness centrality (mean is 0.895 compared to 0.67 in **Table 1**, p=0.000487, Wilcoxon rank-sum test (correlations with closeness centrality in **Table 1** failed the Shapiro normality test with p=0.0019).
[c]Fill time was calculated for each node in each network by applying a perturbation of size 10,000 to the selected node in each time step until more than 80% of the network nodes had an energy level larger than 1. Dissipation was set to 0.

Table S2. Correlation of silencing times calculated for three different-sized starting perturbations in 8 real-world and two benchmark networks

**Network**	**Correlation between low- and medium-intensity perturbations**	**Correlation between medium- and high-intensity perturbations**	**Correlation between low- and high-intensity perturbations**
Benchmark graph with pronounced modules	0.41	**0.90**	0.22
Benchmark graph with fuzzy modules	**0.94**	**0.95**	**0.80**
Substrate-free Met-tRNA synthetase protein structure network	**0.84**	0.78	0.52
Substrate-bound Met-tRNA synthetase protein structure network	**0.84**	0.78	0.56
Filtered Yeast Interactome	**0.88**	**0.81**	0.57
Database of Interacting Proteins yeast interactome (release 2005)	**0.94**	**0.93**	**0.80**
Database of Interacting Proteins yeast interactome (release 2010)	**0.85**	**0.89**	0.63
E. coli metabolic network	**0.99**	**0.97**	**0.96**
B. aphidicola metabolic network	**0.99**	**0.96**	**0.95**
School-friendship network	**0.87**	**0.81**	0.66
**Mean and standard error**	**0.853 (0.053)**	**0.877 (0.024)**	**0.668 (0.070)**

Parameters of the different networks are available in the **Methods** section of the main article. Only the two benchmark graphs were model networks, generated using the benchmark graph generator tool of Lancichinetti and Fortunato [1]. The other graphs originated from different real-world scenarios. Low-intensity perturbation corresponds to an n unit large Dirac-delta starting perturbation, medium-intensity perturbation corresponds to $10*n$ units, and high-intensity means that a $100*n$ unit-sized starting perturbation was applied to a single node when calculating its silencing time, n being the number of nodes in the network. Silencing time was calculated for each node in the network as described in the **Methods** section of the main article. The columns show Spearman's correlation between silencing times calculated for nodes in the same network with different-sized starting perturbations. Correlations above 0.8 were marked with bold letters. It can be noticed that there are no abrupt changes in the importance in the perturbation dissipation capability of different nodes as the perturbation grows larger except for the benchmark network with disjunct modules, which means that real-world networks may display behavior closer to the benchmark graph with fuzzy modules. The table also underlines the choice of choosing $n*10$ as the size of the perturbation when calculating perturbation centrality, since it already seems to display module entrapment effects evidenced by the high correlation observed between medium- and high-intensity perturbations in the benchmark network with disjunct modules compared to the substantially lower correlation observed in the same network between low- and medium-intensity perturbations.

Table S3. Statistically significantly enriched terms in the top 100 protein set of the DIP yeast interactome (release 2005) [7] **containing proteins with *largest absolute* perturbation centrality in differently stressed cases.**

**A. Significantly enriched terms in the top 100 set of largest perturbation centrality in the *unstressed* DIP (2005) interactome**		
**P-value**	**Term**	**Proteins**
3.98e-06	non-homologous end-joining	YGL090W, YOR005C, YMR224C, YCR014C, YMR284W
0.000139	cell cycle	YOR026W, YGR188C, YOR353C, YOR005C, YKR031C, YBR057C, YJR140C, YMR224C, YER018C, YEL061C, YLR254C, YHR184W, YIL132C, YOR368W, YLR045C, YLR190W, YPL253C, YER147C, YGL075C, YGL249W, YJL090C, YGL251C, YLR383W, YCR063W, YML049C, YDR253C, YHL024W, YMR198W, YJL006C
0.00189	cellular response to stimulus	YLR240W, YOR353C, YGL090W, YOR005C, YBR077C, YKR031C, YMR224C, YBR128C, YGL155W, YCR027C, YCR014C, YIL132C, YLR007W, YOR368W, YBR020W, YML112W, YNL145W, YHR134W, YOL043C, YER147C, YIL128W, YJL090C, YHR079C, YLR383W, YMR284W, YOR120W, YLR094C, YJL006C, YPL046C, YGL220W, YDR098C, YDL138W
0.00484	M phase	YOR026W, YGR188C, YKR031C, YBR057C, YMR224C, YER018C, YEL061C, YLR254C, YHR184W, YIL132C, YOR368W, YLR045C, YPL253C, YER147C, YGL075C, YGL249W, YGL251C, YHL024W, YMR198W
0.00637	cell cycle phase	YOR026W, YGR188C, YKR031C, YBR057C, YJR140C, YMR224C, YER018C, YEL061C, YLR254C, YHR184W, YIL132C, YOR368W, YLR045C, YPL253C, YER147C, YGL075C, YGL249W, YJL090C, YGL251C, YHL024W, YMR198W
0.00886	response to DNA damage stimulus	YGL090W, YOR005C, YMR224C, YCR014C, YIL132C, YLR007W, YOR368W, YML112W, YHR134W, YOL043C, YER147C, YIL128W, YJL090C, YLR383W, YMR284W, YJL006C, YPL046C
0.0165	cell cycle process	YOR026W, YGR188C, YKR031C, YBR057C, YJR140C, YMR224C, YER018C, YEL061C, YLR254C, YHR184W, YIL132C, YOR368W, YLR045C, YPL253C, YER147C, YGL075C, YGL249W, YJL090C, YGL251C, YLR383W, YCR063W, YHL024W, YMR198W
0.0212	double-strand break repair	YGL090W, YOR005C, YMR224C, YCR014C, YOR368W, YER147C, YJL090C, YLR383W, YMR284W
0.023	DNA metabolic process	YGL090W, YOR005C, YBR057C, YJR140C, YMR224C, YCR014C, YIL132C, YLR007W, YOR368W, YHR134W, YOL043C, YER147C, YGL249W, YIL128W, YJL090C, YGL251C, YLR383W, YMR284W, YLR010C, YDR082W, YHL024W, YPL046C
0.0256	DNA repair	YGL090W, YOR005C, YMR224C, YCR014C, YIL132C, YLR007W, YOR368W, YHR134W, YOL043C, YER147C, YIL128W, YJL090C, YLR383W, YMR284W, YPL046C
0.048	double-strand break repair via non-homologous end joining	YGL090W, YOR005C, YMR224C, YCR014C, YMR284W
**B. Significantly enriched terms in the top 100 set of largest perturbation centrality in the *heat-shocked* DIP (2005) interactome**		
**P-value**	**Term**	**Proteins**
2.6e-05	condensed chromosome	YOR026W, YGR188C, YBR156C, YER018C, YHR014W, YIL072W, YEL061C, YOL034W, YLR007W, YOR368W, YLR045C, YPL194W, YHR079C-A
0.000126	double-strand break repair via nonhomologous end joining	YGL090W, YOR005C, YMR224C, YHR056C, YCR014C, YJL092W, YDR369C
0.000414	double-strand break repair	YGL090W, YOR005C, YMR224C, YHR056C, YCR014C, YDR004W, YJL092W, YOR368W, YHR079C-A, YDR369C, YPR135W
0.000638	non-recombinational repair	YGL090W, YOR005C, YMR224C, YHR056C, YCR014C, YJL092W, YDR369C
0.00304	condensed nuclear chromosome	YOR026W, YGR188C, YER018C, YHR014W, YIL072W, YEL061C, YOR368W, YLR045C, YPL194W, YHR079C-A

0.0063	recombinational repair	YMR224C, YDR004W, YIL132C, YOL034W, YPL194W, YHR079C-A, YDR369C, YPR135W
0.00869	DNA repair	YGL090W, YOR005C, YMR224C, YHR056C, YCR014C, YDR004W, YJL092W, YIL132C, YOL034W, YLR007W, YOR368W, YPL194W, YHR079C-A, YHR134W, YDR369C, YPR135W
0.0112	cell cycle	YOR026W, YGR188C, YOR353C, YOR005C, YKR031C, YJR140C, YMR224C, YKL092C, YPL255W, YER018C, YHR014W, YKR072C, YDR004W, YIL072W, YEL061C, YLR254C, YHR184W, YIL132C, YOL034W, YOR368W, YLR045C, YLR190W, YPL194W, YHR079C-A, YDR369C, YPR135W
0.0127	nuclear part	YDR312W, YOR026W, YGR188C, YJR002W, YNL075W, YGL090W, YOR005C, YNL182C, YMR033W, YJR140C, YNR054C, YMR224C, YHR056C, YKR092C, YDL148C, YMR025W, YPR112C, YBL018C, YIR009W, YER018C, YHR014W, YJL039C, YBL014C, YDR004W, YIL072W, YEL061C, YOR368W, YLR045C, YPL211W, YOR064C, YML112W, YOR191W, YHR004C, YPL194W, YHR079C-A, YHR134W, YDR369C, YPR135W
0.0138	response to DNA damage stimulus	YGL090W, YOR005C, YMR224C, YHR056C, YCR014C, YDR004W, YJL092W, YIL132C, YOL034W, YLR007W, YOR368W, YML112W, YPL194W, YHR079C-A, YHR134W, YDR369C, YPR135W
0.0304	M phase	YOR026W, YGR188C, YKR031C, YMR224C, YER018C, YHR014W, YDR004W, YIL072W, YEL061C, YLR254C, YHR184W, YIL132C, YOR368W, YLR045C, YPL194W, YHR079C-A, YDR369C, YPR135W
0.0322	cellular response to stimulus	YOR353C, YGL090W, YOR005C, YBR077C, YOL067C, YKR031C, YMR224C, YBR128C, YHR056C, YGL155W, YNL242W, YMR025W, YKL092C, YCR027C, YDL166C, YCR014C, YDR004W, YJL092W, YIL132C, YOL034W, YLR007W, YOR368W, YBR020W, YML112W, YNL145W, YPL194W, YHR079C-A, YHR134W, YDR369C, YPR135W
**C. Significantly enriched terms in the top 100 set of largest perturbation centrality in the *oxidatively stressed* DIP (2005) interactome**		
**P-value**	**Term**	**Proteins**
1.7e-05	cell cycle process	YOR026W, YGR188C, YKR031C, YLR254C, YPL253C, YGL075C, YGL251C, YLR383W, YCR063W, YHL024W, YMR198W, YNL152W, YDR439W, YKL049C, YIL132C, YLR045C, YJL090C, YKR010C, YOR368W, YER149C, YER132C, YEL061C, YJR140C, YER018C, YKL092C, YJL013C, YER147C, YGL174W
2.94e-05	cell cycle	YOR026W, YGR188C, YKR031C, YLR254C, YPL253C, YGL075C, YGL251C, YLR383W, YCR063W, YHL024W, YMR198W, YJL006C, YNL152W, YDR439W, YKL049C, YIL132C, YLR045C, YJL090C, YKR010C, YOR368W, YER149C, YER132C, YEL061C, YJR140C, YDR253C, YER018C, YKL092C, YJL013C, YER147C, YGL174W
0.000225	M phase	YOR026W, YGR188C, YKR031C, YLR254C, YPL253C, YGL075C, YGL251C, YHL024W, YMR198W, YNL152W, YDR439W, YKL049C, YIL132C, YLR045C, YKR010C, YOR368W, YER132C, YEL061C, YER018C, YJL013C, YER147C
0.000284	microtubule motor activity	YPL253C, YMR198W, YKL079W, YDR488C, YEL061C
0.000354	cell cycle phase	YOR026W, YGR188C, YKR031C, YLR254C, YPL253C, YGL075C, YGL251C, YHL024W, YMR198W, YNL152W, YDR439W, YKL049C, YIL132C, YLR045C, YJL090C, YKR010C, YOR368W, YER132C, YEL061C, YJR140C, YER018C, YJL013C, YER147C
0.00104	organelle fission	YOR026W, YGR188C, YLR254C, YPL253C, YGL075C, YMR198W, YNL152W, YDR439W, YKL049C, YKR010C, YIL065C, YEL061C, YER018C, YJL013C, YER147C
0.00294	mitosis	YOR026W, YGR188C, YLR254C, YPL253C, YGL075C, YMR198W, YNL152W, YDR439W, YKL049C, YKR010C, YEL061C, YER018C, YJL013C, YER147C
0.00331	nuclear division	YOR026W, YGR188C, YLR254C, YPL253C, YGL075C, YMR198W, YNL152W, YDR439W, YKL049C, YKR010C, YEL061C, YER018C, YJL013C, YER147C
0.00386	chromosome segregation	YGR188C, YPL253C, YLR383W, YMR198W, YDR439W, YKL049C, YIL132C, YKR010C, YEL061C, YER018C, YJL013C, YER147C
0.00531	nucleus	YOR026W, YGR188C, YKR031C, YKR092C, YBL014C, YCR014C, YLR254C, YHR134W, YPL253C, YDR020C, YJR119C, YGL075C, YHR079C, YGL251C, YLR383W, YCR063W, YKR022C, YMR198W, YJL006C, YPL046C, YGR278W, YDR439W, YKL049C, YFL049W, YGR006W, YBL010C, YIL132C, YGL131C, YLR045C, YPR112C, YHR004C, YJL090C, YNR011C, YDR082W, YKR010C, YDL080C, YOR368W, YLR010C, YLR094C, YIL128W, YHL006C, YAL051W, YEL061C, YJR140C, YDR253C, YGL220W, YMR284W, YER018C, YDR098C, YLR007W, YJL013C, YER147C, YML112W, YGL174W, YHR167W, YPR034W

0.00595	mitotic cell cycle	YOR026W, YGR188C, YLR254C, YPL253C, YGL075C, YMR198W, YNL152W, YDR439W, YKL049C, YLR045C, YJL090C, YKR010C, YEL061C, YJR140C, YDR253C, YER018C, YJL013C, YER147C
0.00851	M phase of mitotic cell cycle	YOR026W, YGR188C, YLR254C, YPL253C, YGL075C, YMR198W, YNL152W, YDR439W, YKL049C, YKR010C, YEL061C, YER018C, YJL013C, YER147C
0.0107	condensed chromosome	YOR026W, YGR188C, YLR383W, YDR439W, YKL049C, YLR045C, YOR368W, YEL061C, YER018C, YLR007W
0.0125	microtubule-based process	YGR188C, YLR254C, YPL253C, YGL075C, YMR198W, YKL079W, YDR488C, YLR045C, YEL061C, YER018C
0.0153	cellular response to stimulus	YKR031C, YCR027C, YCR014C, YNL145W, YHR134W, YHR079C, YLR383W, YJL006C, YPL046C, YIL132C, YJL090C, YBR128C, YBR077C, YOR368W, YER149C, YLR094C, YIL128W, YHL006C, YAL051W, YGL220W, YMR284W, YDL138W, YDR098C, YPL002C, YKL092C, YLR007W, YER147C, YML112W, YBR020W, YLR240W
0.0201	motor activity	YPL253C, YMR198W, YKL079W, YDR488C, YEL061C
0.0256	mitotic sister chromatid segregation	YGR188C, YPL253C, YMR198W, YDR439W, YKL049C, YKR010C, YEL061C, YER147C
0.0257	microtubule-based movement	YLR254C, YMR198W, YKL079W, YDR488C, YEL061C
0.0348	chromosome organization	YGR188C, YPL253C, YJR119C, YGL251C, YMR198W, YNL152W, YDR439W, YKL049C, YFL049W, YIL132C, YDR082W, YKR010C, YLR010C, YHL006C, YEL061C, YJR140C, YMR284W, YER147C, YPR034W
0.0413	sister chromatid segregation	YGR188C, YPL253C, YMR198W, YDR439W, YKL049C, YKR010C, YEL061C, YER147C
0.0443	condensed chromosome kinetochore	YOR026W, YGR188C, YDR439W, YKL049C, YLR045C, YEL061C, YER018C
0.0479	chromosomal part	YOR026W, YGR188C, YLR383W, YDR439W, YKL049C, YGL131C, YLR045C, YJL090C, YDR082W, YOR368W, YLR010C, YEL061C, YMR284W, YER018C, YLR007W, YER147C

D. Significantly enriched terms in the top 100 set of largest perturbation centrality in the *osmotically stressed* DIP (2005) interactome

P-value	Term	Proteins
0.000101	cell cycle process	YGL075C, YGL251C, YLR383W, YCR063W, YDR439W, YPL253C, YGR188C, YKL049C, YHL024W, YOR026W, YJL090C, YLR045C, YMR198W, YJR140C, YHR184W, YER132C, YKR031C, YEL061C, YGL174W, YHL023C, YJL013C, YMR224C, YGR262C, YPR135W, YOR368W, YER018C, YER147C
0.000167	cell cycle	YGL075C, YGL251C, YLR383W, YCR063W, YDR439W, YPL253C, YGR188C, YKL049C, YHL024W, YJL006C, YOR026W, YOR005C, YJL090C, YLR045C, YMR198W, YJR140C, YHR184W, YER132C, YKR031C, YEL061C, YGL174W, YHL023C, YJL013C, YMR224C, YGR262C, YPR135W, YOR368W, YER018C, YER147C
0.00027	microtubule motor activity	YDR488C, YPL253C, YKL079W, YMR198W, YEL061C
0.00028	M phase	YGL075C, YGL251C, YDR439W, YPL253C, YGR188C, YKL049C, YHL024W, YOR026W, YLR045C, YMR198W, YHR184W, YER132C, YKR031C, YEL061C, YHL023C, YJL013C, YMR224C, YPR135W, YOR368W, YER018C, YER147C
0.00028	non-homologous end-joining	YCR014C, YOR005C, YGL090W, YMR224C
0.000453	cell cycle phase	YGL075C, YGL251C, YDR439W, YPL253C, YGR188C, YKL049C, YHL024W, YOR026W, YJL090C, YLR045C, YMR198W, YJR140C, YHR184W, YER132C, YKR031C, YEL061C, YHL023C, YJL013C, YMR224C, YPR135W, YOR368W, YER018C, YER147C
0.00389	combined immunodeficiency	YGR188C, YOR005C, YJL013C
0.00769	nuclear part	YGL075C, YCR063W, YGR278W, YDR439W, YKR092C, YLR010C, YGR188C, YDR082W, YKL049C, YKR022C, YGL131C, YMR219W, YJL006C, YOR026W, YFL049W, YOR005C, YGR006W, YJL090C, YBL014C, YLR045C, YJR140C, YPL046C, YNL286W, YLR051C, YEL061C, YGL174W, YML112W, YGL090W, YHL006C, YJL013C, YMR224C, YKR086W, YPR034W, YPR135W, YOR368W, YER018C, YER147C, YOR064C
0.0109	nucleus	YJR119C, YGL075C, YGL251C, YLR383W, YCR063W, YGR278W, YDR439W, YBL010C, YCR014C, YPL253C, YKR092C, YLR010C, YGR188C, YDR082W, YKL049C, YHR079C, YKR022C, YGL131C, YMR219W, YJL006C, YOR026W, YLR007W, YFL049W, YOR005C, YGR006W, YJL090C, YBL014C, YLR045C, YMR198W, YGL220W, YJR140C, YDR098C, YPL046C,

		YNL286W, YLR051C, YKR031C, YLR014C, YEL061C, YGL174W, YIL128W, YML112W, YGL090W, YDR020C, YNR011C, YHL006C, YJL013C, YMR224C, YKR086W, YGR262C, YPR034W, YOL043C, YPR135W, YOR368W, YER018C, YER147C, YOR064C
0.011	condensed chromosome	YLR383W, YDR439W, YGR188C, YKL049C, YOR026W, YLR007W, YLR045C, YEL061C, YOR368W, YER018C
0.0128	upslanted palpebral fissure	YJR119C, YGR188C, YOR005C, YEL061C, YJL013C
0.0133	chromosomal part	YLR383W, YDR439W, YLR010C, YGR188C, YDR082W, YKL049C, YGL131C, YOR026W, YLR007W, YJL090C, YLR045C, YEL061C, YGR262C, YPR135W, YOR368W, YER018C, YER147C
0.019	motor activity	YDR488C, YPL253C, YKL079W, YMR198W, YEL061C
0.0216	double-strand break repair	YLR383W, YCR014C, YOR005C, YJL090C, YGL090W, YMR224C, YPR135W, YOR368W, YER147C
0.0252	chromosome segregation	YLR383W, YDR439W, YPL253C, YGR188C, YKL049C, YMR198W, YEL061C, YJL013C, YPR135W, YER018C, YER147C
0.0253	macromolecular complex	YLR383W, YCR063W, YGR278W, YDR439W, YDR488C, YPL253C, YLR010C, YBR128C, YGR188C, YDR082W, YKL049C, YKR022C, YGL131C, YLR439W, YJL006C, YOR026W, YLR007W, YFL049W, YOR005C, YNL014W, YKL079W, YGR006W, YNR049C, YJL090C, YBL014C, YLR045C, YMR198W, YLR185W, YJR140C, YPL046C, YNL286W, YEL061C, YGL174W, YML112W, YGL090W, YHL023C, YLR240W, YPL002C, YHL006C, YJL013C, YMR224C, YKR086W, YGR262C, YBR077C, YPR034W, YPR135W, YIL068C, YOR368W, YER018C, YER147C, YOR064C
0.0255	mitotic sister chromatid segregation	YDR439W, YPL253C, YGR188C, YKL049C, YMR198W, YEL061C, YPR135W, YER147C
0.0256	DNA metabolic process	YGL251C, YLR383W, YDR439W, YCR014C, YLR010C, YDR082W, YKL049C, YHL024W, YLR007W, YOR005C, YJL090C, YJR140C, YPL046C, YIL128W, YGL090W, YHL006C, YMR224C, YGR262C, YOL043C, YPR135W, YOR368W, YER147C
0.0289	cafe-au-lait spot	YJR119C, YGR188C, YOR005C, YJL013C
0.0322	organelle fission	YGL075C, YDR439W, YPL253C, YGR188C, YKL049C, YOR026W, YMR198W, YIL065C, YEL061C, YJL013C, YPR135W, YER018C, YER147C
0.0369	protein complex	YLR383W, YDR439W, YDR488C, YPL253C, YBR128C, YGR188C, YKL049C, YGL131C, YJL006C, YOR026W, YLR007W, YFL049W, YOR005C, YKL079W, YNR049C, YJL090C, YBL014C, YLR045C, YMR198W, YJR140C, YPL046C, YEL061C, YGL174W, YML112W, YGL090W, YHL023C, YLR240W, YPL002C, YHL006C, YJL013C, YMR224C, YGR262C, YBR077C, YPR034W, YPR135W, YIL068C, YOR368W, YER018C, YER147C, YOR064C
0.0395	response to DNA damage stimulus	YLR383W, YCR014C, YJL006C, YLR007W, YOR005C, YJL090C, YPL046C, YIL128W, YML112W, YGL090W, YHL006C, YMR224C, YOL043C, YPR135W, YOR368W, YER147C
0.0411	sister chromatid segregation	YDR439W, YPL253C, YGR188C, YKL049C, YMR198W, YEL061C, YPR135W, YER147C
0.0433	condensed chromosome kinetochore	YDR439W, YGR188C, YKL049C, YOR026W, YLR045C, YEL061C, YER018C

Perturbation centralities were calculated with the Turbine software as described in **Methods** of the main text. Term enrichment analysis was performed with the R plug-in of g:Profiler [12], which returns both the enriched terms, and the proteins connected with the term. A term was stated as statistically significant, if the resulting p-value was strictly less than 0.05 after applying Bonferroni correction. Results show the high importance of cell cycle maintenance and DNA repair in both stressed and unstressed cases.

Table S4. Statistically significantly enriched terms in the top 100 protein set of the DIP yeast interactome (release 2005) [7] **containing proteins with *largest increase* of perturbation centrality in differently stressed cases.**

A. Significantly enriched terms in the top 100 set of proteins having largest *increase* of perturbation centrality on *heat shock* (compared to the unstressed case)		
**P-value**	**Term**	**Proteins**
5.18e-08	cellular carbohydrate metabolic process	YLR273C, YJL137C, YIL045W, YNL216W, YPR160W, YMR261C, YML100W, YDR074W, YCR005C, YLR258W, YPL201C, YNR001C, YBR149W, YIL053W, YER062C, YJL089W, YMR105C, YPL180W
6.15e-07	response to stimulus	YDL190C, YDL059C, YNL076W, YBR169C, YNL103W, YNL216W, YER103W, YMR261C, YEL060C, YML100W, YDR074W, YFL021W, YDL124W, YBL075C, YNL314W, YIR023W, YIL113W, YLR178C, YPL022W, YLR019W, YOL128C, YGR088W, YPL154C, YFL016C, YPR054W, YIL053W, YDR168W, YDR214W, YER062C, YOR363C, YKL109W, YDR200C, YOR120W, YGL163C, YLR259C, YDL017W, YMR250W, YJL089W, YHR186C, YLL021W, YPL180W, YNR007C, YBR274W, YJR032W
1.25e-06	cellular carbohydrate biosynthetic process	YLR273C, YJL137C, YIL045W, YMR261C, YML100W, YDR074W, YLR258W, YPL201C, YIL053W, YER062C, YMR105C
2.31e-05	carbohydrate biosynthetic process	YLR273C, YJL137C, YIL045W, YMR261C, YML100W, YDR074W, YLR258W, YPL201C, YIL053W, YER062C, YJL089W, YMR105C
0.000188	response to stress	YDL190C, YDL059C, YBR169C, YNL216W, YER103W, YMR261C, YEL060C, YML100W, YDR074W, YDL124W, YBL075C, YPL022W, YLR019W, YOL128C, YGR088W, YPL154C, YFL016C, YIL053W, YDR168W, YDR214W, YER062C, YOR120W, YGL163C, YLR259C, YDL017W, YMR250W, YHR186C, YPL180W, YNR007C, YBR274W, YJR032W
0.000323	organic substance catabolic process	YPL065W, YJL172W, YIR032C, YDL190C, YLR270W, YCL008C, YCL052C, YNL216W, YOR173W, YPR160W, YPR111W, YNL230C, YEL060C, YER143W, YNL314W, YCR005C, YIR023W, YPL022W, YNR001C, YPL154C, YFL016C, YKL148C, YDR214W, YOR363C, YOR120W, YGR058W, YLL041C, YNL311C, YMR250W, YMR105C, YMR287C
0.000354	catalytic activity	YJL137C, YML016C, YJL172W, YIR032C, YMR315W, YDL190C, YLR270W, YCL008C, YAL060W, YBR265W, YOR173W, YOR317W, YER103W, YPR160W, YLR096W, YIL177C, YMR261C, YPR111W, YPL074W, YEL060C, YML100W, YDR074W, YDL124W, YBL075C, YER143W, YCR005C, YLR258W, YMR104C, YIL113W, YER081W, YML065W, YPL022W, YLR019W, YOL128C, YGR088W, YNR001C, YBR149W, YPL154C, YIL108W, YPR054W, YIL053W, YBL045C, YNL092W, YKL148C, YPR191W, YER062C, YOR120W, YGL163C, YLR259C, YDL017W, YLL041C, YMR250W, YMR105C, YKL210W, YNR007C, YMR287C, YBR274W, YJR032W
0.00046	catabolic process	YPL065W, YJL172W, YIR032C, YDL190C, YLR270W, YCL008C, YCL052C, YNL216W, YOR173W, YPR160W, YPR111W, YNL230C, YEL060C, YER143W, YNL314W, YCR005C, YIR023W, YPL022W, YGR088W, YNR001C, YPL154C, YFL016C, YKL148C, YDR214W, YOR363C, YOR120W, YGR058W, YLL041C, YNL311C, YMR250W, YMR105C, YNR007C, YMR287C
0.000616	cellular catabolic process	YPL065W, YJL172W, YIR032C, YDL190C, YLR270W, YCL008C, YCL052C, YNL216W, YOR173W, YPR111W, YNL230C, YEL060C, YER143W, YNL314W, YCR005C, YIR023W, YPL022W, YGR088W, YNR001C, YPL154C, YFL016C, YKL148C, YDR214W, YOR363C, YGR058W, YLL041C, YNL311C, YMR250W, YNR007C, YMR287C
0.00102	energy derivation by oxidation of organic compounds	YLR273C, YJL137C, YIL045W, YPR160W, YCR005C, YLR258W, YNR001C, YBL045C, YKL148C, YPR191W, YKL109W, YLL041C, YMR105C
0.00105	oxidation-reduction process	YLR273C, YJL137C, YMR315W, YIL045W, YAL060W, YBR265W, YPR160W, YDL124W, YCR005C, YLR258W, YER081W, YGR088W, YNR001C, YBR149W, YBL045C, YKL148C, YPR191W, YOR363C, YKL109W, YOR120W, YLL041C, YMR105C
0.00116	carbohydrate metabolic process	YLR273C, YJL137C, YIL045W, YNL216W, YPR160W, YMR261C, YML100W, YDR074W, YCR005C, YLR258W, YPL201C, YNR001C, YBR149W, YIL053W, YER062C, YOR120W, YJL089W, YMR105C, YPL180W
0.00276	glycoside biosynthetic process	YMR261C, YML100W, YDR074W, YMR105C
0.00276	oligosaccharide biosynthetic	YMR261C, YML100W, YDR074W, YMR105C

	process	
0.00276	disaccharide biosynthetic process	YMR261C, YML100W, YDR074W, YMR105C
0.00276	trehalose biosynthetic process	YMR261C, YML100W, YDR074W, YMR105C
0.00547	alditol biosynthetic process	YPL201C, YIL053W, YER062C
0.00547	glycerol biosynthetic process	YPL201C, YIL053W, YER062C
0.00873	generation of precursor metabolites and energy	YLR273C, YJL137C, YIL045W, YNL216W, YPR160W, YCR005C, YLR258W, YNR001C, YBL045C, YKL148C, YPR191W, YKL109W, YLL041C, YMR105C
0.0139	glycogen metabolic process	YLR273C, YJL137C, YIL045W, YPR160W, YLR258W, YMR105C
0.0156	glycogen biosynthetic process	YLR273C, YJL137C, YIL045W, YLR258W, YMR105C
0.0188	glycogen breakdown (glycogenolysis)	YJL137C, YPR160W, YMR105C
0.0191	organic substance metabolic process	YLR273C, YDR034C, YJL137C, YPL065W, YML016C, YJL172W, YIR032C, YMR315W, YDL190C, YLR270W, YDL059C, YNL076W, YCL008C, YIL045W, YBR169C, YAL060W, YNL103W, YBR265W, YCL052C, YNL216W, YOR173W, YOR317W, YER103W, YPR160W, YLR096W, YMR261C, YPR111W, YNL230C, YEL060C, YML100W, YDR074W, YFL021W, YDL124W, YBL075C, YLR453C, YER143W, YNL314W, YCR005C, YLR258W, YIR023W, YMR104C, YIL113W, YER081W, YLR178C, YPL201C, YML065W, YPL022W, YOL128C, YNR001C, YBR149W, YPL154C, YIL108W, YFL016C, YPR054W, YIL053W, YBL045C, YDR168W, YKL148C, YPR191W, YDR214W, YER062C, YOR363C, YKL109W, YOR120W, YGL163C, YLR259C, YGR058W, YDL017W, YLL041C, YNL311C, YMR250W, YJL089W, YMR105C, YKL210W, YPL180W, YNR007C, YMR287C, YDR515W, YBR274W, YJR032W
0.0199	energy reserve metabolic process	YLR273C, YJL137C, YIL045W, YPR160W, YLR258W, YMR105C
0.0217	alpha,alpha-trehalose-phosphate synthase complex (UDP-forming)	YMR261C, YML100W, YDR074W
0.0248	trehalose metabolic process	YMR261C, YML100W, YDR074W, YMR105C
0.0368	fungal-type vacuole lumen	YJL172W, YEL060C, YLR178C, YPL154C
0.0437	single-organism metabolic process	YLR273C, YDR034C, YJL137C, YPL065W, YML016C, YJL172W, YIR032C, YMR315W, YDL190C, YLR270W, YDL059C, YNL076W, YCL008C, YIL045W, YBR169C, YAL060W, YNL103W, YBR265W, YCL052C, YNL216W, YOR173W, YOR317W, YER103W, YPR160W, YLR096W, YMR261C, YPR111W, YNL230C, YEL060C, YML100W, YDR074W, YFL021W, YDL124W, YBL075C, YLR453C, YER143W, YNL314W, YCR005C, YLR258W, YIR023W, YMR104C, YIL113W, YER081W, YLR178C, YPL201C, YML065W, YPL022W, YOL128C, YGR088W, YNR001C, YBR149W, YPL154C, YIL108W, YFL016C, YPR054W, YIL053W, YBL045C, YDR168W, YKL148C, YPR191W, YDR214W, YER062C, YOR363C, YKL109W, YOR120W, YGL163C, YLR259C, YGR058W, YDL017W, YLL041C, YNL311C, YMR250W, YJL089W, YMR105C, YKL210W, YPL180W, YNR007C, YMR287C, YDR515W, YBR274W, YJR032W

B. Significantly enriched terms in the top 100 set of proteins having largest *increase* of perturbation centrality in *oxidative stress* (compared to the unstressed case)

P-value	Term	Proteins
4.24e-07	cellular carbohydrate metabolic process	YLR273C, YJL137C, YIL045W, YML100W, YMR261C, YDR074W, YLR258W, YGR143W, YPR160W, YBR149W, YGR166W, YCR005C, YOR178C, YNR001C, YJL089W, YDR001C, YBL058W
2.2e-05	cellular carbohydrate biosynthetic process	YLR273C, YJL137C, YIL045W, YML100W, YMR261C, YDR074W, YLR258W, YGR143W, YGR166W, YOR178C
8.53e-05	glucan metabolic process	YLR273C, YJL137C, YIL045W, YLR258W, YGR143W, YPR160W, YGR166W, YOR178C, YBL058W
8.53e-05	cellular glucan metabolic process	YLR273C, YJL137C, YIL045W, YLR258W, YGR143W, YPR160W, YGR166W, YOR178C, YBL058W
0.000254	carbohydrate biosynthetic process	YLR273C, YJL137C, YIL045W, YML100W, YMR261C, YDR074W, YLR258W, YGR143W, YGR166W, YOR178C, YJL089W
0.000585	glucan biosynthetic process	YLR273C, YJL137C, YIL045W, YLR258W, YGR143W, YGR166W, YOR178C

0.000596	cellular polysaccharide metabolic process	YLR273C, YJL137C, YIL045W, YLR258W, YGR143W, YPR160W, YGR166W, YOR178C, YBL058W
0.000649	TRAPP complex	YOR115C, YDR246W, YGR166W, YMR218C, YDR407C
0.000928	glycogen metabolic process	YLR273C, YJL137C, YIL045W, YLR258W, YPR160W, YOR178C, YBL058W
0.00102	carbohydrate metabolic process	YLR273C, YJL137C, YIL045W, YML100W, YMR261C, YDR074W, YLR258W, YGR143W, YPR160W, YBR149W, YBR229C, YGR166W, YCR005C, YOR178C, YMR200W, YNR001C, YJL089W, YDR001C, YBL058W
0.00143	energy reserve metabolic process	YLR273C, YJL137C, YIL045W, YLR258W, YPR160W, YOR178C, YBL058W
0.00194	response to stimulus	YLR309C, YJL173C, YNL076W, YER103W, YPR019W, YML032C, YDL124W, YMR250W, YDR453C, YDR179C, YLR178C, YPL026C, YML100W, YMR261C, YDR074W, YBR202W, YPL154C, YHL035C, YDL059C, YOL128C, YBR216C, YFR014C, YOR018W, YMR038C, YOR178C, YDR049W, YMR200W, YMR218C, YJL201W, YKL213C, YJL089W, YDR168W, YDR001C, YDL216C, YDL101C, YBL058W, YDR407C
0.00249	polysaccharide metabolic process	YLR273C, YJL137C, YIL045W, YLR258W, YGR143W, YPR160W, YGR166W, YOR178C, YBL058W
0.00852	cellular polysaccharide biosynthetic process	YLR273C, YJL137C, YIL045W, YLR258W, YGR143W, YGR166W, YOR178C
0.00993	polysaccharide biosynthetic process	YLR273C, YJL137C, YIL045W, YLR258W, YGR143W, YGR166W, YOR178C
0.0142	oxidation-reduction process	YMR315W, YLR273C, YJL137C, YIL045W, YDL124W, YBR265W, YDR453C, YLR258W, YPR160W, YDR231C, YBR149W, YBR213W, YMR038C, YNL202W, YLL051C, YCR005C, YOR178C, YER023W, YNR001C, YBL058W
0.0168	acetyl-CoA + H_2O + oxaloacetate => citrate + CoA	YCR005C, YNR001C
0.0196	glycogen biosynthetic process	YLR273C, YJL137C, YIL045W, YLR258W, YOR178C
0.0196	cis-Golgi network	YOR115C, YDR246W, YGR166W, YMR218C, YDR407C
0.0289	alpha,alpha-trehalose-phosphate synthase complex (UDP-forming)	YML100W, YMR261C, YDR074W
0.0321	trehalose metabolic process	YML100W, YMR261C, YDR074W, YDR001C
0.0477	protein phosphatase type 1 regulator activity	YLR273C, YIL045W, YOR178C, YBL058W
**C. Significantly enriched terms in the top 100 set of proteins having largest *increase* of perturbation centrality in *osmotic stress* (compared to the unstressed case)**		
**P-value**	**Term**	**Proteins**
4.42e-09	cellular carbohydrate biosynthetic process	YLR273C, YJL137C, YIL045W, YPL201C, YIL053W, YER062C, YLR258W, YMR261C, YFR015C, YML100W, YDR074W, YMR105C, YOR178C
2.66e-06	carbohydrate biosynthetic process	YLR273C, YJL137C, YIL045W, YPL201C, YIL053W, YER062C, YLR258W, YMR261C, YFR015C, YML100W, YDR074W, YMR105C, YOR178C
5.85e-06	cellular carbohydrate metabolic process	YLR273C, YJL137C, YIL045W, YPL201C, YIL053W, YER062C, YLR258W, YMR261C, YFR015C, YIR031C, YML100W, YDR074W, YCR005C, YDR001C, YMR105C, YOR178C
1.98e-05	glycogen biosynthetic process	YLR273C, YJL137C, YIL045W, YLR258W, YFR015C, YMR105C, YOR178C
0.000582	glucan biosynthetic process	YLR273C, YJL137C, YIL045W, YLR258W, YFR015C, YMR105C, YOR178C
0.000586	trehalose metabolic process	YMR261C, YML100W, YDR074W, YDR001C, YMR105C
6e-04	protein phosphorylation	YJR059W, YKL116C, YKL048C, YDL025C, YLR096W, YFL033C, YGR052W, YPL203W, YLR210W, YMR104C, YDR460W, YFL029C, YER129W, YDR052C, YDR490C
0.000619	protein serine/threonine kinase activity	YJR059W, YKL116C, YKL048C, YDL025C, YLR096W, YFL033C, YGR052W, YPL203W, YMR104C, YFL029C, YER129W, YDR490C
0.000922	glycogen metabolic process	YLR273C, YJL137C, YIL045W, YLR258W, YFR015C, YMR105C, YOR178C

0.00104	protein kinase activity	YJR059W, YKL116C, YKL048C, YDL025C, YLR096W, YFL033C, YGR052W, YPL203W, YMR104C, YFL029C, YER129W, YDR490C
0.00141	energy reserve metabolic process	YLR273C, YJL137C, YIL045W, YLR258W, YFR015C, YMR105C, YOR178C
0.00306	glycoside biosynthetic process	YMR261C, YML100W, YDR074W, YMR105C
0.00306	oligosaccharide biosynthetic process	YMR261C, YML100W, YDR074W, YMR105C
0.00306	disaccharide biosynthetic process	YMR261C, YML100W, YDR074W, YMR105C
0.00306	trehalose biosynthetic process	YMR261C, YML100W, YDR074W, YMR105C
0.00316	phosphorylation	YJR059W, YKL116C, YKL048C, YKL067W, YDL025C, YLR096W, YHR033W, YFL033C, YGR052W, YPL203W, YLR210W, YMR104C, YDR460W, YFL029C, YER129W, YDR052C, YDR490C, YKL141W
0.00372	response to stress	YDR159W, YIL053W, YER103W, YBR169C, YER062C, YDR501W, YMR261C, YKL048C, YML100W, YDR074W, YDL124W, YKL067W, YCR065W, YOR141C, YDL190C, YJL173C, YDR217C, YJR032W, YFL033C, YDL020C, YDR001C, YDR460W, YDL059C, YGR088W, YBR066C, YDR113C, YGL163C, YLR183C, YOR178C
0.00586	response to stimulus	YDR159W, YIL053W, YER103W, YNL076W, YBR169C, YER062C, YDR379W, YDR501W, YMR261C, YKL116C, YKL048C, YML100W, YDR074W, YDL124W, YKL067W, YCR065W, YAL024C, YOR141C, YDL190C, YJL173C, YDR217C, YJR032W, YFL033C, YHL035C, YDL020C, YPL203W, YDR001C, YDR460W, YDL059C, YOR018W, YGR088W, YBR066C, YDR113C, YGL163C, YLR183C, YOR178C, YDR490C
0.00595	alditol biosynthetic process	YPL201C, YIL053W, YER062C
0.00595	glycerol biosynthetic process	YPL201C, YIL053W, YER062C
0.007	kinase activity	YJR059W, YKL116C, YKL048C, YKL067W, YDL025C, YLR096W, YHR033W, YFL033C, YGR052W, YPL203W, YMR104C, YFL029C, YER129W, YDR490C
0.00716	carbohydrate metabolic process	YLR273C, YJL137C, YIL045W, YPL201C, YIL053W, YER062C, YLR258W, YMR261C, YFR015C, YIR031C, YKL048C, YML100W, YDR074W, YCR005C, YDR001C, YMR105C, YER129W, YOR178C
0.00839	cellular polysaccharide biosynthetic process	YLR273C, YJL137C, YIL045W, YLR258W, YFR015C, YMR105C, YOR178C
0.00958	cell cycle phase	YDR159W, YJR059W, YDR285W, YOR058C, YPL155C, YGR238C, YOR195W, YGL216W, YCR065W, YAL024C, YOR177C, YJL173C, YDR217C, YFL033C, YLR210W, YNL309W, YFL029C, YNL068C, YDR052C, YDR113C, YOR178C
0.00977	polysaccharide biosynthetic process	YLR273C, YJL137C, YIL045W, YLR258W, YFR015C, YMR105C, YOR178C
0.0127	glycogen synthesis	YJL137C, YLR258W, YFR015C, YMR105C
0.0133	glycoside metabolic process	YMR261C, YML100W, YDR074W, YDR001C, YMR105C
0.0156	single-organism carbohydrate metabolic process	YLR273C, YJL137C, YIL045W, YPL201C, YIL053W, YER062C, YLR258W, YMR261C, YFR015C, YKL048C, YML100W, YDR074W, YDR001C, YMR105C, YER129W, YOR178C
0.0173	glucan metabolic process	YLR273C, YJL137C, YIL045W, YLR258W, YFR015C, YMR105C, YOR178C
0.0173	cellular glucan metabolic process	YLR273C, YJL137C, YIL045W, YLR258W, YFR015C, YMR105C, YOR178C
0.0235	alpha,alpha-trehalose-phosphate synthase complex (UDP-forming)	YMR261C, YML100W, YDR074W
0.0252	cell cycle process	YDR159W, YJR059W, YDR285W, YOR058C, YKL048C, YPL155C, YGR238C, YOR195W, YGL216W, YCR065W, YAL024C, YOR177C, YJL173C, YDR217C, YFL033C, YLR210W, YNL309W, YFL029C, YNL068C, YDR052C, YDR113C, YLR457C, YOR178C
0.0323	cell cycle	YDR159W, YJR059W, YDR285W, YOR058C, YKL048C, YPL155C, YGR238C, YOR195W, YGL216W, YCR065W, YAL024C, YOR177C, YJL173C, YDR217C, YFL033C, YLR210W, YDR460W, YNL309W, YFL029C, YNL068C, YDR052C, YDR113C, YNL007C, YLR457C, YOR178C
0.0365	mitotic cell cycle	YDR159W, YJR059W, YOR058C, YPL155C, YGR238C, YOR195W, YGL216W, YCR065W, YAL024C, YDR217C, YLR210W,

		YNL309W, YFL029C, YNL068C, YDR052C, YDR113C, YOR178C

Perturbation centralities were calculated with the Turbine software as described in **Methods** of the main text. Term enrichment analysis was performed with the R plug-in of g:Profiler [12], which returns both the enriched terms, and the proteins connected with the term. A term was stated as statistically significant, if the resulting p-value was strictly less than 0.05 after applying Bonferroni correction. Results clearly show the stress response as displayed by the significant enrichment of the terms “response to stimulus” and “response to stress”. Furthermore, the strong up-regulation of carbohydrate metabolism is also obvious from the data, and it is a well-known stress response [13–15] successfully identified by the perturbation centrality measure.

Table S5. Most significantly enriched terms in the top 100 protein set of the DIP yeast interactome (release 2005) [7] containing proteins with *largest decrease* of perturbation centrality in differently stressed cases.

A. Significantly enriched terms in the top 100 set of proteins having *largest decrease* of perturbation centrality in *heat shock*		
**P-value**	**Term**	**Proteins**
1.81e-59	ribosome biogenesis	YCL054W, YLR129W, YOL010W, YPL093W, YPR137W, YDR324C, YDR449C, YLR276C, YMR239C, YHR088W, YLL008W, YGR159C, YGL171W, YJL033W, YNL124W, YOL077C, YLR186W, YPL217C, YFR001W, YDL166C, YOR310C, YKL099C, YDL031W, YHR072W-A, YKL009W, YOR206W, YHR170W, YGR128C, YHR148W, YBR267W, YHR066W, YJR002W, YDL148C, YHR197W, YKR060W, YPL211W, YKL021C, YNL075W, YJL109C, YEL026W, YGL111W, YBR247C, YGR245C, YDR087C, YGL078C, YLR221C, YHR081W, YMR093W, YJL069C, YOR243C, YIR026C, YGR081C, YPR144C, YNL308C, YER126C, YNL182C, YKL082C, YMR229C, YNR053C, YER006W, YKR081C, YOL142W, YHR052W, YCR072C, YMR128W, YER002W, YPL226W, YNL232W, YNR054C, YLR397C, YDL060W
6.76e-57	nucleolus	YCL054W, YLR129W, YOL010W, YPL093W, YPR137W, YDR324C, YDR449C, YLR276C, YMR239C, YHR088W, YLL008W, YGR159C, YNL248C, YGL171W, YJL033W, YOL077C, YLR186W, YPL217C, YFR001W, YOR310C, YKL099C, YDL031W, YHR072W-A, YKL009W, YOR206W, YGR128C, YHR148W, YHR066W, YJR002W, YDL148C, YOR340C, YHR143W-A, YKR060W, YPL211W, YKL021C, YNL075W, YJL109C, YEL026W, YGL111W, YBR247C, YGR245C, YDR087C, YGL078C, YLR221C, YMR093W, YJL069C, YJL076W, YGR081C, YPR144C, YNL308C, YER126C, YKL082C, YMR229C, YNR053C, YER006W, YKR081C, YOL142W, YHR052W, YCR072C, YMR128W, YER002W, YNL232W, YNR054C, YPL020C, YDL060W, YNL175C
4.68e-56	ribonucleoprotein complex biogenesis	YCL054W, YLR129W, YOL010W, YPL093W, YPR137W, YDR324C, YDR449C, YLR276C, YMR239C, YHR088W, YLL008W, YGR159C, YGL171W, YJL033W, YNL124W, YOL077C, YLR186W, YPL217C, YFR001W, YDL166C, YOR310C, YKL099C, YDL031W, YHR072W-A, YKL009W, YOR206W, YHR170W, YGR128C, YHR148W, YBR267W, YHR066W, YJR002W, YDL148C, YHR197W, YKR060W, YPL211W, YKL021C, YNL075W, YJL109C, YEL026W, YGL111W, YBR247C, YGR245C, YDR087C, YGL078C, YLR221C, YOR276W, YHR081W, YMR093W, YJL069C, YOR243C, YIR026C, YGR081C, YPR144C, YNL308C, YER126C, YNL182C, YKL082C, YMR229C, YNR053C, YER006W, YKR081C, YOL142W, YHR052W, YCR072C, YMR128W, YER002W, YPL226W, YNL232W, YNR054C, YLR397C, YDL060W
7.16e-52	preribosome	YCL054W, YLR129W, YOL010W, YPL093W, YPR137W, YDR324C, YDR449C, YHR088W, YLL008W, YGL171W, YOL077C, YLR186W, YPL217C, YFR001W, YOR310C, YKL099C, YDL031W, YKL009W, YOR206W, YGR128C, YHR148W, YJR002W, YDL148C, YKR060W, YPL211W, YNL075W, YJL109C, YEL026W, YGL111W, YBR247C, YDR087C, YGL078C, YLR221C, YMR093W, YJL069C, YGR081C, YPR144C, YER126C, YMR229C, YNR053C, YER006W, YKR081C, YHR052W, YMR128W, YER002W, YLR397C, YDL060W, YNL175C
1.75e-49	cellular component biogenesis at cellular level	YCL054W, YLR129W, YOL010W, YPL093W, YPR137W, YDR324C, YDR449C, YLR276C, YMR239C, YHR088W, YLL008W, YGR159C, YGL171W, YJL033W, YNL124W, YOL077C, YLR186W, YPL217C, YFR001W, YDL166C, YOR310C, YKL099C, YDL031W, YHR072W-A, YKL009W, YOR206W, YHR170W, YGR128C, YHR148W, YBR267W, YHR066W, YJR002W, YDL148C, YHR197W, YKR060W, YPL211W, YKL021C, YNL075W, YJL109C, YEL026W, YGL111W, YBR247C, YGR245C, YDR087C, YGL078C, YLR221C, YOR276W, YHR081W, YMR093W, YJL069C, YOR243C, YIR026C, YGR081C, YPR144C, YNL308C, YER126C, YNL182C, YKL082C, YMR229C, YNR053C, YER006W, YKR081C, YOL142W, YHR052W, YCR072C, YMR128W, YER002W, YPL226W, YNL232W, YNR054C, YLR397C, YDL060W
3.56e-48	nuclear lumen	YCL054W, YLR129W, YOL010W, YPL093W, YPR137W, YDR324C, YDR449C, YLR276C, YMR239C, YHR088W, YLL008W, YGR159C, YNL248C, YGL171W, YJL033W, YNL124W, YOL077C, YLR186W, YPL217C, YFR001W, YNL151C, YOR310C, YKL099C, YDL031W, YKL144C, YHR072W-A, YKL009W, YOR206W, YHR170W, YGR128C, YHR148W, YHR066W, YPR190C, YJR002W, YDL148C, YHR197W, YOR340C, YHR143W-A, YKR060W, YPL211W, YKL021C, YNL075W, YJL109C, YEL026W, YGL111W, YBR247C, YGR245C, YDR087C, YHR031C, YGL078C, YLR221C, YOR207C, YHR081W, YMR093W, YJL069C, YJL076W, YGR081C, YPR144C, YNL308C, YER126C, YNL182C, YKL082C, YMR229C, YNR053C, YDR045C, YER006W, YKR081C, YOL142W, YHR052W, YCR072C, YMR128W, YER002W, YNL232W, YNR054C, YPL233W, YPL020C, YDL060W, YNL175C

1.91e-47	rRNA processing	YCL054W, YLR129W, YOL010W, YPL093W, YPR137W, YDR324C, YDR449C, YLR276C, YMR239C, YHR088W, YLL008W, YGR159C, YGL171W, YJL033W, YNL124W, YLR186W, YPL217C, YFR001W, YDL166C, YOR310C, YKL099C, YDL031W, YHR072W-A, YKL009W, YGR128C, YHR148W, YJR002W, YDL148C, YHR197W, YKR060W, YPL211W, YKL021C, YNL075W, YJL109C, YEL026W, YGL111W, YBR247C, YDR087C, YGL078C, YHR081W, YMR093W, YJL069C, YOR243C, YGR081C, YPR144C, YNL308C, YER126C, YNL182C, YKL082C, YMR229C, YER006W, YKR081C, YOL142W, YMR128W, YER002W, YNL232W, YNR054C, YDL060W
1.84e-46	rRNA metabolic process	YCL054W, YLR129W, YOL010W, YPL093W, YPR137W, YDR324C, YDR449C, YLR276C, YMR239C, YHR088W, YLL008W, YGR159C, YGL171W, YJL033W, YNL124W, YLR186W, YPL217C, YFR001W, YDL166C, YOR310C, YKL099C, YDL031W, YHR072W-A, YKL009W, YGR128C, YHR148W, YJR002W, YDL148C, YHR197W, YKR060W, YPL211W, YKL021C, YNL075W, YJL109C, YEL026W, YGL111W, YBR247C, YDR087C, YGL078C, YHR081W, YMR093W, YJL069C, YOR243C, YGR081C, YPR144C, YNL308C, YER126C, YNL182C, YKL082C, YMR229C, YER006W, YKR081C, YOL142W, YMR128W, YER002W, YNL232W, YNR054C, YDL060W
2.09e-41	ncRNA processing	YCL054W, YLR129W, YOL010W, YPL093W, YPR137W, YDR324C, YDR449C, YLR276C, YMR239C, YHR088W, YLL008W, YGR159C, YGL171W, YJL033W, YNL124W, YLR186W, YPL217C, YFR001W, YDL166C, YOR310C, YKL099C, YDL031W, YHR072W-A, YKL009W, YGR128C, YHR148W, YJR002W, YDL148C, YHR197W, YKR060W, YPL211W, YKL021C, YNL075W, YJL109C, YEL026W, YGL111W, YBR247C, YDR087C, YGL078C, YNL062C, YHR081W, YMR093W, YJL069C, YOR243C, YGR081C, YPR144C, YNL308C, YER126C, YNL182C, YKL082C, YMR229C, YER006W, YKR081C, YOL142W, YMR128W, YER002W, YNL232W, YNR054C, YDL060W
1.78e-39	ncRNA metabolic process	YCL054W, YLR129W, YOL010W, YPL093W, YPR137W, YDR324C, YDR449C, YLR276C, YMR239C, YHR088W, YLL008W, YGR159C, YGL171W, YJL033W, YNL124W, YLR186W, YPL217C, YFR001W, YDL166C, YOR310C, YKL099C, YDL031W, YHR072W-A, YKL009W, YGR128C, YHR148W, YJR002W, YDL148C, YHR197W, YKR060W, YPL211W, YKL021C, YNL075W, YJL109C, YEL026W, YGL111W, YBR247C, YDR087C, YGL078C, YNL062C, YHR081W, YMR093W, YJL069C, YOR243C, YGR081C, YPR144C, YNL308C, YER126C, YNL182C, YKL082C, YMR229C, YER006W, YKR081C, YOL142W, YMR128W, YER002W, YNL232W, YNR054C, YDR037W, YDL060W
7.58e-39	organelle lumen	YCL054W, YLR129W, YOL010W, YPL093W, YPR137W, YDR324C, YDR449C, YLR276C, YMR239C, YHR088W, YLL008W, YGR159C, YNL248C, YGL171W, YJL033W, YNL124W, YOL077C, YLR186W, YPL217C, YFR001W, YNL151C, YOR310C, YKL099C, YDL031W, YKL144C, YHR072W-A, YKL009W, YOR206W, YHR170W, YGR128C, YHR148W, YHR066W, YPR190C, YJR002W, YDL148C, YHR197W, YOR340C, YHR143W-A, YKR060W, YPL211W, YKL021C, YNL075W, YJL109C, YEL026W, YGL111W, YBR247C, YGR245C, YDR087C, YHR031C, YGL078C, YLR221C, YOR207C, YHR081W, YMR093W, YJL069C, YJL076W, YGR081C, YPR144C, YNL308C, YER126C, YNL182C, YKL082C, YMR229C, YNR053C, YDR045C, YER006W, YKR081C, YOL142W, YHR052W, YCR072C, YMR128W, YER002W, YNL232W, YNR054C, YPL233W, YPL020C, YDL060W, YNL175C
7.58e-39	intracellular organelle lumen	YCL054W, YLR129W, YOL010W, YPL093W, YPR137W, YDR324C, YDR449C, YLR276C, YMR239C, YHR088W, YLL008W, YGR159C, YNL248C, YGL171W, YJL033W, YNL124W, YOL077C, YLR186W, YPL217C, YFR001W, YNL151C, YOR310C, YKL099C, YDL031W, YKL144C, YHR072W-A, YKL009W, YOR206W, YHR170W, YGR128C, YHR148W, YHR066W, YPR190C, YJR002W, YDL148C, YHR197W, YOR340C, YHR143W-A, YKR060W, YPL211W, YKL021C, YNL075W, YJL109C, YEL026W, YGL111W, YBR247C, YGR245C, YDR087C, YHR031C, YGL078C, YLR221C, YOR207C, YHR081W, YMR093W, YJL069C, YJL076W, YGR081C, YPR144C, YNL308C, YER126C, YNL182C, YKL082C, YMR229C, YNR053C, YDR045C, YER006W, YKR081C, YOL142W, YHR052W, YCR072C, YMR128W, YER002W, YNL232W, YNR054C, YPL233W, YPL020C, YDL060W, YNL175C
1.14e-38	membrane-enclosed lumen	YCL054W, YLR129W, YOL010W, YPL093W, YPR137W, YDR324C, YDR449C, YLR276C, YMR239C, YHR088W, YLL008W, YGR159C, YNL248C, YGL171W, YJL033W, YNL124W, YOL077C, YLR186W, YPL217C, YFR001W, YNL151C, YOR310C, YKL099C, YDL031W, YKL144C, YHR072W-A, YKL009W, YOR206W, YHR170W, YGR128C, YHR148W, YHR066W, YPR190C, YJR002W, YDL148C, YHR197W, YOR340C, YHR143W-A, YKR060W, YPL211W, YKL021C, YNL075W, YJL109C, YEL026W, YGL111W, YBR247C, YGR245C, YDR087C, YHR031C, YGL078C, YLR221C, YOR207C, YHR081W, YMR093W, YJL069C, YJL076W, YGR081C, YPR144C, YNL308C, YER126C, YFR011C, YNL182C, YKL082C, YMR229C, YNR053C, YDR045C, YER006W, YKR081C, YOL142W, YHR052W, YCR072C, YMR128W, YER002W, YNL232W, YNR054C, YPL233W, YPL020C, YDL060W, YNL175C
2.48e-34	nuclear part	YCL054W, YLR129W, YOL010W, YPL093W, YPR137W, YDR324C, YDR449C, YLR276C, YMR239C, YHR088W, YLL008W, YGR159C, YNL248C, YGL171W, YJL033W, YNL124W, YOL077C, YLR186W, YPL217C, YFR001W, YNL151C, YOR310C, YKL099C, YDL031W, YKL144C,

		YHR072W-A, YKL009W, YOR206W, YHR170W, YGR128C, YHR148W, YHR066W, YPR190C, YJR002W, YDL148C, YHR197W, YOR340C, YHR143W-A, YKR060W, YPL211W, YKL021C, YNL075W, YJL109C, YEL026W, YGL111W, YBR247C, YGR245C, YDR087C, YHR031C, YGL078C, YLR221C, YOR207C, YHR081W, YMR093W, YJL069C, YJL076W, YGR081C, YPR144C, YNL308C, YER126C, YNL182C, YKL082C, YMR229C, YNR053C, YDR045C, YER006W, YKR081C, YOL142W, YHR052W, YCR072C, YMR128W, YER002W, YNL232W, YNR054C, YPL233W, YPL020C, YDL060W, YNL175C
6.61e-32	RNA processing	YCL054W, YLR129W, YOL010W, YPL093W, YPR137W, YDR324C, YDR449C, YLR276C, YMR239C, YHR088W, YLL008W, YGR159C, YGL171W, YJL033W, YNL124W, YLR186W, YPL217C, YFR001W, YDL166C, YOR310C, YKL099C, YDL031W, YHR072W-A, YKL009W, YGR128C, YHR148W, YJR002W, YDL148C, YHR197W, YKR060W, YPL211W, YKL021C, YNL075W, YJL109C, YEL026W, YGL111W, YBR247C, YDR087C, YGL078C, YNL062C, YHR081W, YMR093W, YJL069C, YOR243C, YGR081C, YPR144C, YNL308C, YER126C, YNL182C, YKL082C, YMR229C, YER006W, YKR081C, YOL142W, YMR128W, YER002W, YNL232W, YNR054C, YDL060W
1.67e-31	cellular component biogenesis	YCL054W, YLR129W, YOL010W, YPL093W, YPR137W, YDR324C, YDR449C, YLR276C, YMR239C, YHR088W, YLL008W, YGR159C, YGL171W, YJL033W, YNL124W, YOL077C, YLR186W, YPL217C, YFR001W, YDL166C, YOR310C, YKL099C, YDL031W, YHR072W-A, YKL009W, YOR206W, YHR170W, YGR128C, YHR148W, YBR267W, YHR066W, YJR002W, YDL148C, YHR197W, YKR060W, YPL211W, YKL021C, YNL075W, YJL109C, YEL026W, YGL111W, YBR247C, YGR245C, YDR087C, YGL078C, YLR221C, YOR276W, YHR081W, YMR093W, YJL069C, YOR243C, YIR026C, YGR081C, YPR144C, YNL308C, YER126C, YNL182C, YKL082C, YMR229C, YNR053C, YER006W, YKR081C, YOL142W, YHR052W, YCR072C, YMR128W, YER002W, YPL226W, YNL232W, YNR054C, YLR397C, YDL060W
1.47e-28	ribosomal large subunit biogenesis	YCL054W, YPL093W, YLR276C, YHR088W, YLL008W, YOL077C, YFR001W, YDL031W, YKL009W, YBR267W, YHR066W, YHR197W, YPL211W, YKL021C, YGL111W, YGR245C, YGL078C, YLR221C, YIR026C, YER126C, YNL182C, YKL082C, YMR229C, YKR081C, YHR052W, YCR072C, YER002W, YLR397C
4.33e-27	maturation of 5.8S rRNA from tricistronic rRNA transcript (SSU-rRNA, 5.8S rRNA, LSU-rRNA)	YCL054W, YLR129W, YOL010W, YDR449C, YHR088W, YGL171W, YLR186W, YFR001W, YOR310C, YKL099C, YDL031W, YJR002W, YDL148C, YKL021C, YJL109C, YBR247C, YGL078C, YHR081W, YJL069C, YPR144C, YNL308C, YER126C, YMR229C, YKR081C, YOL142W, YNL232W, YNR054C
6.41e-27	maturation of 5.8S rRNA	YCL054W, YLR129W, YOL010W, YDR449C, YHR088W, YGL171W, YLR186W, YFR001W, YOR310C, YKL099C, YDL031W, YJR002W, YDL148C, YKL021C, YJL109C, YBR247C, YGL078C, YHR081W, YJL069C, YPR144C, YNL308C, YER126C, YMR229C, YKR081C, YOL142W, YNL232W, YNR054C
2.17e-25	non-membrane-bounded organelle	YCL054W, YLR129W, YOL010W, YPL093W, YPR137W, YDR324C, YDR449C, YLR276C, YMR239C, YFR031C-A, YHR088W, YLL008W, YGR159C, YNL248C, YGL171W, YJL033W, YOL077C, YLR186W, YPL217C, YFR001W, YOR310C, YKL099C, YDL031W, YHR072W-A, YKL009W, YOR206W, YGR128C, YHR148W, YHR066W, YJR002W, YDL148C, YOR340C, YHR143W-A, YKR060W, YPL211W, YKL021C, YNL075W, YJL109C, YEL026W, YGL111W, YBR247C, YGR245C, YDR087C, YHR031C, YGL078C, YLR221C, YMR093W, YJL069C, YJL076W, YGR081C, YPR144C, YNL308C, YER126C, YKL082C, YMR229C, YNR053C, YER006W, YKR081C, YOL142W, YHR052W, YCR072C, YMR128W, YER002W, YNL232W, YNR054C, YPL233W, YPL020C, YDL060W, YNL175C
2.17e-25	intracellular non-membrane-bounded organelle	YCL054W, YLR129W, YOL010W, YPL093W, YPR137W, YDR324C, YDR449C, YLR276C, YMR239C, YFR031C-A, YHR088W, YLL008W, YGR159C, YNL248C, YGL171W, YJL033W, YOL077C, YLR186W, YPL217C, YFR001W, YOR310C, YKL099C, YDL031W, YHR072W-A, YKL009W, YOR206W, YGR128C, YHR148W, YHR066W, YJR002W, YDL148C, YOR340C, YHR143W-A, YKR060W, YPL211W, YKL021C, YNL075W, YJL109C, YEL026W, YGL111W, YBR247C, YGR245C, YDR087C, YHR031C, YGL078C, YLR221C, YMR093W, YJL069C, YJL076W, YGR081C, YPR144C, YNL308C, YER126C, YKL082C, YMR229C, YNR053C, YER006W, YKR081C, YOL142W, YHR052W, YCR072C, YMR128W, YER002W, YNL232W, YNR054C, YPL233W, YPL020C, YDL060W, YNL175C
5.69e-25	ribonucleoprotein complex	YCL054W, YLR129W, YOL010W, YPL093W, YPR137W, YDR324C, YDR449C, YFR031C-A, YHR088W, YLL008W, YGL171W, YNL124W, YOL077C, YLR186W, YPL217C, YFR001W, YOR310C, YKL099C, YDL031W, YHR072W-A, YKL009W, YOR206W, YGR128C, YHR148W, YJR002W, YDL148C, YKR060W, YPL211W, YNL075W, YJL109C, YEL026W, YGL111W, YBR247C, YDR087C, YGL078C, YLR221C,

		YMR093W, YJL069C, YGR081C, YPR144C, YER126C, YMR229C, YCL037C, YNR053C, YER006W, YKR081C, YHR052W, YMR128W, YER002W, YPL226W, YLR397C, YDL060W, YAL036C, YNL175C
2.39e-24	preribosome, large subunit precursor	YCL054W, YPL093W, YHR088W, YLL008W, YOL077C, YFR001W, YDL031W, YKL009W, YOR206W, YPL211W, YGL111W, YDR087C, YGL078C, YLR221C, YER126C, YNR053C, YER006W, YKR081C, YHR052W, YER002W, YLR397C
5.36e-24	nucleus	YCL054W, YLR129W, YOL010W, YPL093W, YPR137W, YDR324C, YDR449C, YLR276C, YMR239C, YHR088W, YLL008W, YGR159C, YNL248C, YGL171W, YJL033W, YNL124W, YOL077C, YLR186W, YPL217C, YFR001W, YDL166C, YNL151C, YOR310C, YKL099C, YDL031W, YKL144C, YDR399W, YHR072W-A, YKL009W, YOR206W, YHR170W, YGR128C, YHR148W, YHR066W, YPR190C, YJR002W, YDL148C, YHR197W, YOR340C, YHR143W-A, YKR060W, YPL211W, YKL021C, YNL075W, YJL109C, YEL026W, YGL111W, YBR247C, YGR245C, YDR087C, YHR031C, YGL078C, YNL062C, YLR221C, YOR207C, YHR081W, YMR093W, YJL069C, YJL076W, YOR243C, YIR026C, YGR081C, YPR144C, YNL308C, YER126C, YNL182C, YKL082C, YMR229C, YNR053C, YDR045C, YMR310C, YER006W, YML021C, YKR081C, YOL142W, YHR052W, YCR072C, YMR128W, YER002W, YPL226W, YNL232W, YNR054C, YPL233W, YPL020C, YPR189W, YDL060W, YNL175C
2.08e-22	ribosomal small subunit biogenesis	YLR129W, YOL010W, YDR324C, YDR449C, YGR159C, YGL171W, YLR186W, YFR001W, YDL166C, YOR310C, YKL099C, YGR128C, YJR002W, YDL148C, YKR060W, YJL109C, YEL026W, YBR247C, YMR093W, YJL069C, YGR081C, YPR144C, YNL308C, YKL082C, YMR229C, YMR128W, YPL226W, YNR054C
3.22e-22	RNA metabolic process	YCL054W, YLR129W, YOL010W, YPL093W, YPR137W, YDR324C, YDR449C, YLR276C, YMR239C, YHR088W, YLL008W, YGR159C, YNL248C, YGL171W, YJL033W, YNL124W, YLR186W, YPL217C, YFR001W, YDL166C, YNL151C, YOR310C, YKL099C, YDL031W, YKL144C, YHR072W-A, YKL009W, YGR128C, YHR148W, YPR190C, YJR002W, YDL148C, YHR197W, YOR340C, YHR143W-A, YKR060W, YPL211W, YKL021C, YNL075W, YJL109C, YEL026W, YGL111W, YBR247C, YDR087C, YGL078C, YNL062C, YOR207C, YHR081W, YMR093W, YJL069C, YJL076W, YOR243C, YGR081C, YPR144C, YNL308C, YER126C, YNL182C, YKL082C, YMR229C, YDR045C, YER006W, YKR081C, YOL142W, YMR128W, YER002W, YNL232W, YNR054C, YDR037W, YPR189W, YDL060W
3.68e-22	90S preribosome	YLR129W, YOL010W, YPR137W, YDR324C, YDR449C, YGL171W, YLR186W, YPL217C, YOR310C, YOR206W, YGR128C, YHR148W, YJR002W, YDL148C, YKR060W, YNL075W, YJL109C, YBR247C, YMR093W, YJL069C, YGR081C, YPR144C, YMR229C, YMR128W, YDL060W
6.62e-21	cleavage involved in rRNA processing	YLR129W, YOL010W, YDR449C, YGL171W, YLR186W, YFR001W, YOR310C, YKL099C, YHR072W-A, YJR002W, YDL148C, YJL109C, YBR247C, YGL078C, YHR081W, YJL069C, YPR144C, YNL308C, YMR229C, YOL142W, YNL232W, YNR054C
9.54e-21	RNA phosphodiester bond hydrolysis	YLR129W, YOL010W, YDR449C, YGL171W, YLR186W, YFR001W, YOR310C, YKL099C, YHR072W-A, YJR002W, YDL148C, YJL109C, YBR247C, YGL078C, YHR081W, YJL069C, YPR144C, YNL308C, YMR229C, YOL142W, YNL232W, YNR054C
4.42e-20	maturation of SSU-rRNA from tricistronic rRNA transcript (SSU-rRNA, 5.8S rRNA, LSU-rRNA)	YLR129W, YOL010W, YDR324C, YDR449C, YGL171W, YLR186W, YFR001W, YDL166C, YOR310C, YKL099C, YGR128C, YJR002W, YDL148C, YJL109C, YEL026W, YBR247C, YMR093W, YJL069C, YGR081C, YPR144C, YNL308C, YMR229C, YMR128W, YNR054C
**B. Significantly enriched terms in the top 100 set of proteins having *largest decrease* of perturbation centrality in *oxidative stress***		
**P-value**	**Term**	**Protein**
6.62e-38	ribosome biogenesis	YMR239C, YKL099C, YPL217C, YDL148C, YGL171W, YDR324C, YNL308C, YOL077C, YLR186W, YDR299W, YKR060W, YPR137W, YGL111W, YPL211W, YHR088W, YGL078C, YMR229C, YKL082C, YDL166C, YGR128C, YPL226W, YLR129W, YOR206W, YPR144C, YLR002C, YBR267W, YFR001W, YHR066W, YHR148W, YNL182C, YLR409C, YLR221C, YJR002W, YMR093W, YLL008W, YOR078W, YNL124W, YNR054C, YHR196W, YLR276C, YFL002C, YER002W, YJL069C, YHR170W, YOR243C, YDR398W, YPR112C, YNL075W, YHR081W, YKL021C, YGL099W, YHR197W, YBR247C, YKR024C, YHR052W, YNL224C
5.98e-37	preribosome	YKL099C, YPL217C, YDL148C, YGL171W, YDR324C, YOL077C, YLR186W, YDR299W, YKR060W, YPR137W, YGL111W, YPL211W, YHR088W, YGL078C, YMR229C, YGR128C, YLR129W, YOR206W, YPR144C, YLR002C, YFR001W, YHR148W, YLR409C, YLR221C, YJR002W, YMR093W, YLL008W, YOR078W, YHR196W, YFL002C, YER002W, YJL069C, YNL175C, YDR398W, YPR112C, YNL075W, YBR247C, YHR052W, YNL224C

1.99e-35	ribonucleoprotein complex biogenesis	YMR239C, YKL099C, YPL217C, YDL148C, YGL171W, YDR324C, YNL308C, YOL077C, YLR186W, YDR299W, YKR060W, YPR137W, YGL111W, YPL211W, YHR088W, YGL078C, YMR229C, YKL082C, YDL166C, YGR128C, YPL226W, YLR129W, YOR206W, YPR144C, YLR002C, YBR267W, YFR001W, YHR066W, YHR148W, YNL182C, YLR409C, YLR221C, YJR002W, YMR093W, YLL008W, YOR078W, YNL124W, YNR054C, YHR196W, YLR276C, YFL002C, YER002W, YJL069C, YHR170W, YOR243C, YDR398W, YPR112C, YNL075W, YIR005W, YHR081W, YKL021C, YGL099W, YHR197W, YBR247C, YKR024C, YHR052W, YNL224C
4.58e-34	nucleolus	YMR239C, YKL099C, YPL217C, YDL148C, YGL171W, YNL248C, YDR324C, YNL308C, YOL077C, YLR186W, YDR299W, YKR060W, YPR137W, YGL111W, YPL211W, YHR088W, YGL078C, YMR229C, YKL082C, YGR128C, YLR129W, YOR206W, YPR144C, YLR002C, YEL055C, YFR001W, YHR066W, YHR148W, YLR409C, YLR221C, YOR340C, YJR002W, YMR093W, YLL008W, YOR078W, YNR054C, YHR196W, YLR276C, YFL002C, YER002W, YJL069C, YNL175C, YDR398W, YPR112C, YNL075W, YKL021C, YBR247C, YKR024C, YNL113W, YHR052W
2.38e-32	rRNA processing	YMR239C, YKL099C, YPL217C, YDL148C, YGL171W, YDR324C, YNL308C, YLR186W, YDR299W, YKR060W, YPR137W, YGL111W, YPL211W, YHR088W, YGL078C, YMR229C, YKL082C, YDL166C, YGR128C, YLR129W, YPR144C, YLR002C, YFR001W, YHR148W, YNL182C, YLR409C, YJR002W, YMR093W, YLL008W, YOR078W, YNL124W, YNR054C, YHR196W, YLR276C, YFL002C, YER002W, YJL069C, YOR243C, YDR398W, YPR112C, YNL075W, YHR081W, YKL021C, YHR197W, YBR247C, YKR024C, YNL224C
1.39e-31	rRNA metabolic process	YMR239C, YKL099C, YPL217C, YDL148C, YGL171W, YDR324C, YNL308C, YLR186W, YDR299W, YKR060W, YPR137W, YGL111W, YPL211W, YHR088W, YGL078C, YMR229C, YKL082C, YDL166C, YGR128C, YLR129W, YPR144C, YLR002C, YFR001W, YHR148W, YNL182C, YLR409C, YJR002W, YMR093W, YLL008W, YOR078W, YNL124W, YNR054C, YHR196W, YLR276C, YFL002C, YER002W, YJL069C, YOR243C, YDR398W, YPR112C, YNL075W, YHR081W, YKL021C, YHR197W, YBR247C, YKR024C, YNL224C
1.82e-30	cellular component biogenesis at cellular level	YMR239C, YKL099C, YPL217C, YDL148C, YGL171W, YDR324C, YNL308C, YOL077C, YLR186W, YDR299W, YKR060W, YPR137W, YGL111W, YPL211W, YHR088W, YGL078C, YMR229C, YKL082C, YDL166C, YGR128C, YPL226W, YLR129W, YOR206W, YPR144C, YLR002C, YBR267W, YFR001W, YHR066W, YHR148W, YNL182C, YLR409C, YLR221C, YJR002W, YMR093W, YLL008W, YOR078W, YNL124W, YNR054C, YHR196W, YLR276C, YFL002C, YER002W, YJL069C, YHR170W, YOR243C, YDR398W, YPR112C, YNL075W, YIR005W, YHR081W, YKL021C, YGL099W, YHR197W, YBR247C, YKR024C, YHR052W, YNL224C
9.66e-30	nuclear lumen	YMR239C, YKL099C, YPL217C, YDL148C, YGL171W, YNL248C, YDR324C, YNL308C, YOL077C, YLR186W, YNL151C, YPR190C, YDR299W, YKR060W, YPR137W, YGL111W, YPL211W, YHR088W, YGL078C, YMR229C, YKL082C, YGR128C, YLR129W, YKL144C, YDR227W, YOR206W, YPR144C, YLR002C, YEL055C, YFR001W, YHR066W, YHR148W, YNL182C, YLR409C, YLR221C, YOR340C, YJR002W, YMR093W, YLL008W, YOR078W, YNL124W, YNR054C, YHR196W, YLR276C, YFL002C, YER002W, YMR176W, YJL069C, YHR170W, YNL175C, YDR398W, YPR112C, YNL075W, YHR081W, YKL021C, YBR289W, YHR197W, YBR247C, YKR024C, YNL113W, YOL012C, YHR052W, YOR207C
1.34e-26	ncRNA processing	YMR239C, YKL099C, YPL217C, YDL148C, YGL171W, YDR324C, YNL308C, YLR186W, YDR299W, YKR060W, YPR137W, YGL111W, YPL211W, YHR088W, YGL078C, YMR229C, YKL082C, YDL166C, YGR128C, YLR129W, YPR144C, YLR002C, YFR001W, YHR148W, YNL182C, YLR409C, YJR002W, YMR093W, YLL008W, YOR078W, YNL124W, YNR054C, YHR196W, YLR276C, YFL002C, YER002W, YJL069C, YOR243C, YDR398W, YPR112C, YNL075W, YHR081W, YKL021C, YHR197W, YBR247C, YKR024C, YNL224C
3.12e-25	ncRNA metabolic process	YMR239C, YKL099C, YPL217C, YDL148C, YGL171W, YDR324C, YNL308C, YLR186W, YDR299W, YKR060W, YPR137W, YGL111W, YPL211W, YHR088W, YOL097C, YGL078C, YMR229C, YKL082C, YDL166C, YGR128C, YLR129W, YPR144C, YLR002C, YFR001W, YHR148W, YNL182C, YLR409C, YJR002W, YMR093W, YLL008W, YOR078W, YNL124W, YNR054C, YHR196W, YLR276C, YFL002C, YER002W, YJL069C, YOR243C, YDR398W, YPR112C, YNL075W, YHR081W, YKL021C, YHR197W, YBR247C, YKR024C, YNL224C
1.01e-23	90S preribosome	YPL217C, YDL148C, YGL171W, YDR324C, YLR186W, YDR299W, YKR060W, YPR137W, YMR229C, YGR128C, YLR129W, YOR206W, YPR144C, YHR148W, YLR409C, YJR002W, YMR093W, YOR078W, YHR196W, YFL002C, YJL069C, YDR398W, YPR112C, YNL075W, YBR247C, YNL224C
1.2e-22	organelle lumen	YMR239C, YKL099C, YPL217C, YDL148C, YGL171W, YNL248C, YDR324C, YNL308C, YOL077C, YLR186W, YNL151C, YPR190C, YDR299W, YKR060W, YPR137W, YGL111W, YPL211W, YHR088W, YGL078C, YMR229C, YKL082C, YGR128C, YLR129W, YKL144C, YDR227W, YOR206W, YPR144C, YLR002C, YEL055C, YFR001W, YHR066W, YHR148W, YNL182C, YLR409C, YLR221C, YOR340C, YJR002W,

		YMR093W, YLL008W, YOR078W, YNL124W, YNR054C, YHR196W, YLR276C, YFL002C, YER002W, YMR176W, YJL069C, YHR170W, YNL175C, YDR398W, YPR112C, YNL075W, YHR081W, YKL021C, YBR289W, YHR197W, YBR247C, YKR024C, YNL113W, YOL012C, YHR052W, YOR207C
1.2e-22	intracellular organelle lumen	YMR239C, YKL099C, YPL217C, YDL148C, YGL171W, YNL248C, YDR324C, YNL308C, YOL077C, YLR186W, YNL151C, YPR190C, YDR299W, YKR060W, YPR137W, YGL111W, YPL211W, YHR088W, YGL078C, YMR229C, YKL082C, YGR128C, YLR129W, YKL144C, YDR227W, YOR206W, YPR144C, YLR002C, YEL055C, YFR001W, YHR066W, YHR148W, YNL182C, YLR409C, YLR221C, YOR340C, YJR002W, YMR093W, YLL008W, YOR078W, YNL124W, YNR054C, YHR196W, YLR276C, YFL002C, YER002W, YMR176W, YJL069C, YHR170W, YNL175C, YDR398W, YPR112C, YNL075W, YHR081W, YKL021C, YBR289W, YHR197W, YBR247C, YKR024C, YNL113W, YOL012C, YHR052W, YOR207C
1.64e-22	membrane-enclosed lumen	YMR239C, YKL099C, YPL217C, YDL148C, YGL171W, YNL248C, YDR324C, YNL308C, YOL077C, YLR186W, YNL151C, YPR190C, YDR299W, YKR060W, YPR137W, YGL111W, YPL211W, YHR088W, YGL078C, YMR229C, YKL082C, YGR128C, YLR129W, YKL144C, YDR227W, YOR206W, YPR144C, YLR002C, YEL055C, YFR001W, YHR066W, YHR148W, YNL182C, YLR409C, YLR221C, YOR340C, YJR002W, YMR093W, YLL008W, YOR078W, YNL124W, YNR054C, YHR196W, YLR276C, YFL002C, YER002W, YMR176W, YJL069C, YHR170W, YNL175C, YDR398W, YPR112C, YNL075W, YFR011C, YHR081W, YKL021C, YBR289W, YHR197W, YBR247C, YKR024C, YNL113W, YOL012C, YHR052W, YOR207C
4.57e-21	nuclear part	YMR239C, YKL099C, YPL217C, YDL148C, YGL171W, YNL248C, YDR324C, YNL308C, YOL077C, YLR186W, YNL151C, YPR190C, YDR299W, YKR060W, YPR137W, YGL111W, YPL211W, YHR088W, YGL078C, YMR229C, YKL082C, YGR128C, YLR129W, YKL144C, YDR227W, YOR206W, YPR144C, YLR002C, YEL055C, YFR001W, YHR066W, YHR148W, YNL182C, YLR409C, YLR221C, YOR340C, YJR002W, YMR093W, YLL008W, YOR078W, YNL124W, YNR054C, YHR196W, YLR276C, YFL002C, YER002W, YMR176W, YJL069C, YHR170W, YNL175C, YDR398W, YPR112C, YDR303C, YNL075W, YIR005W, YHR081W, YKL021C, YBR289W, YHR197W, YBR247C, YKR024C, YNL113W, YOL012C, YHR052W, YOR207C
2.28e-20	RNA processing	YMR239C, YKL099C, YPL217C, YDL148C, YGL171W, YDR324C, YNL308C, YLR186W, YDR299W, YKR060W, YPR137W, YGL111W, YPL211W, YHR088W, YGL078C, YMR229C, YKL082C, YDL166C, YGR128C, YLR129W, YPR144C, YLR002C, YFR001W, YHR148W, YNL182C, YLR409C, YJR002W, YMR093W, YLL008W, YOR078W, YNL124W, YNR054C, YHR196W, YLR276C, YFL002C, YER002W, YJL069C, YOR243C, YDR398W, YPR112C, YNL075W, YIR005W, YHR081W, YKL021C, YHR197W, YBR247C, YKR024C, YNL224C
**C. Significantly enriched terms in the top 100 set of proteins having *largest decrease* of perturbation centrality in *osmotic stress***		
**P-value**	**Term**	**Proteins**
1.29e-40	ribosome biogenesis	YGL111W, YNL308C, YMR239C, YGL078C, YHR066W, YOL077C, YFL002C, YOR206W, YKL172W, YLR276C, YLR002C, YDL166C, YMR229C, YIR026C, YDL148C, YDR299W, YDR324C, YKL009W, YKL099C, YCL054W, YNL182C, YGR103W, YBR267W, YLR221C, YLR129W, YOR243C, YNL124W, YGR128C, YHR170W, YGR081C, YPL226W, YOR294W, YPL211W, YHR052W, YPR102C, YHR088W, YPR137W, YKL021C, YHR197W, YKR081C, YHR072W-A, YFR001W, YDR091C, YHR148W, YNL061W, YDR087C, YGL099W, YLR409C, YPR112C, YNL002C, YOL010W, YNL207W, YPR144C, YNL075W, YPL126W, YLR186W, YNL224C, YGR159C
2.56e-39	ribonucleoprotein complex biogenesis	YGL111W, YNL308C, YMR239C, YGL078C, YHR066W, YOL077C, YFL002C, YOR206W, YKL172W, YLR276C, YLR002C, YDL166C, YMR229C, YIR026C, YDL148C, YDR299W, YDR324C, YKL009W, YKL099C, YCL054W, YNL182C, YGR103W, YBR267W, YLR221C, YIR005W, YLR129W, YOR243C, YNL124W, YGR128C, YHR170W, YGR081C, YPL226W, YOR294W, YPL211W, YHR052W, YPR102C, YHR088W, YPR137W, YKL021C, YHR197W, YKR081C, YHR072W-A, YFR001W, YDR091C, YHR148W, YNL061W, YDR087C, YGL099W, YGR178C, YLR409C, YPR112C, YNL002C, YOL010W, YNL207W, YPR144C, YNL075W, YPL126W, YLR186W, YNL224C, YGR159C
5.54e-37	preribosome	YGL111W, YGL078C, YOL077C, YFL002C, YOR206W, YKL172W, YLR002C, YMR229C, YDL148C, YDR299W, YDR324C, YKL009W, YKL099C, YCL054W, YNL175C, YGR103W, YLR221C, YLR129W, YGR128C, YGR081C, YOR294W, YPL211W, YHR052W, YHR088W, YPR137W, YKR081C, YFR001W, YHR148W, YNL061W, YDR087C, YLR409C, YPR112C, YNL002C, YOL010W, YPR144C, YNL075W, YPL126W, YLR186W, YNL224C
2.1e-35	nucleolus	YGL111W, YNL308C, YMR239C, YGL078C, YNL248C, YHR066W, YOL077C, YFL002C, YOR206W, YKL172W, YEL055C, YLR276C, YLR002C, YMR229C, YDL148C, YDR299W, YDR324C, YKL009W, YKL099C, YCL054W, YNL175C, YGR103W, YLR221C, YOR340C, YLR129W,

		YGR128C, YGR081C, YOR294W, YPL211W, YHR052W, YHR088W, YPR137W, YKL021C, YKR081C, YHR072W-A, YFR001W, YHR143W-A, YHR148W, YNL061W, YDR087C, YPL020C, YLR409C, YPR112C, YNL002C, YOL010W, YPR144C, YNL075W, YPL126W, YLR186W, YGR159C, YNL113W
4.88e-34	cellular component biogenesis at cellular level	YGL111W, YNL308C, YMR239C, YGL078C, YHR066W, YOL077C, YFL002C, YOR206W, YKL172W, YLR276C, YLR002C, YDL166C, YMR229C, YIR026C, YDL148C, YDR299W, YDR324C, YKL009W, YKL099C, YCL054W, YNL182C, YGR103W, YBR267W, YLR221C, YIR005W, YLR129W, YOR243C, YNL124W, YGR128C, YHR170W, YGR081C, YPL226W, YOR294W, YPL211W, YHR052W, YPR102C, YHR088W, YPR137W, YKL021C, YHR197W, YKR081C, YHR072W-A, YFR001W, YDR091C, YHR148W, YNL061W, YDR087C, YGL099W, YGR178C, YLR409C, YPR112C, YNL002C, YOL010W, YNL207W, YPR144C, YNL075W, YPL126W, YLR186W, YNL224C, YGR159C
2.21e-32	rRNA processing	YGL111W, YNL308C, YMR239C, YGL078C, YFL002C, YKL172W, YLR276C, YLR002C, YDL166C, YMR229C, YDL148C, YDR299W, YDR324C, YKL009W, YKL099C, YCL054W, YNL182C, YGR103W, YLR129W, YOR243C, YNL124W, YGR128C, YGR081C, YOR294W, YPL211W, YHR088W, YPR137W, YKL021C, YHR197W, YKR081C, YHR072W-A, YFR001W, YDR091C, YHR148W, YNL061W, YDR087C, YLR409C, YPR112C, YNL002C, YOL010W, YNL207W, YPR144C, YNL075W, YPL126W, YLR186W, YNL224C, YGR159C
1.29e-31	rRNA metabolic process	YGL111W, YNL308C, YMR239C, YGL078C, YFL002C, YKL172W, YLR276C, YLR002C, YDL166C, YMR229C, YDL148C, YDR299W, YDR324C, YKL009W, YKL099C, YCL054W, YNL182C, YGR103W, YLR129W, YOR243C, YNL124W, YGR128C, YGR081C, YOR294W, YPL211W, YHR088W, YPR137W, YKL021C, YHR197W, YKR081C, YHR072W-A, YFR001W, YDR091C, YHR148W, YNL061W, YDR087C, YLR409C, YPR112C, YNL002C, YOL010W, YNL207W, YPR144C, YNL075W, YPL126W, YLR186W, YNL224C, YGR159C
1.67e-27	ncRNA metabolic process	YGL111W, YNL308C, YMR239C, YGL078C, YFL002C, YKL172W, YLR276C, YLR002C, YDL166C, YMR229C, YOL097C, YDL148C, YDR299W, YDR324C, YKL009W, YKL099C, YCL054W, YNL182C, YGR102C, YGR103W, YLR129W, YOR243C, YNL124W, YGR128C, YGR081C, YOR294W, YPL211W, YHR088W, YPR137W, YKL021C, YHR197W, YKR081C, YHR072W-A, YFR001W, YDR091C, YHR148W, YNL061W, YDR087C, YLR409C, YPR112C, YNL002C, YOL010W, YNL207W, YPR144C, YNL075W, YPL126W, YLR186W, YNL224C, YHR019C, YGR159C
1.24e-26	ncRNA processing	YGL111W, YNL308C, YMR239C, YGL078C, YFL002C, YKL172W, YLR276C, YLR002C, YDL166C, YMR229C, YDL148C, YDR299W, YDR324C, YKL009W, YKL099C, YCL054W, YNL182C, YGR103W, YLR129W, YOR243C, YNL124W, YGR128C, YGR081C, YOR294W, YPL211W, YHR088W, YPR137W, YKL021C, YHR197W, YKR081C, YHR072W-A, YFR001W, YDR091C, YHR148W, YNL061W, YDR087C, YLR409C, YPR112C, YNL002C, YOL010W, YNL207W, YPR144C, YNL075W, YPL126W, YLR186W, YNL224C, YGR159C
1.69e-25	nuclear lumen	YGL111W, YNL308C, YMR239C, YGL078C, YNL248C, YHR066W, YOL077C, YFL002C, YOR206W, YKL172W, YEL055C, YLR276C, YLR002C, YMR229C, YDL148C, YDR299W, YDR324C, YKL009W, YKL099C, YCL054W, YNL182C, YNL175C, YGR103W, YLR221C, YOR340C, YLR129W, YNL124W, YKL144C, YGR128C, YHR170W, YGR081C, YOR294W, YPL211W, YHR052W, YHR088W, YPR137W, YKL021C, YHR197W, YKR081C, YHR072W-A, YFR001W, YHR143W-A, YHR148W, YNL061W, YDR087C, YPL020C, YCR092C, YLR409C, YPR112C, YNL002C, YOL010W, YPR144C, YNL075W, YPL126W, YLR186W, YMR167W, YHR031C, YGR159C, YNL113W
2.12e-25	ribosomal large subunit biogenesis	YGL111W, YGL078C, YHR066W, YOL077C, YFL002C, YLR276C, YMR229C, YIR026C, YKL009W, YCL054W, YNL182C, YGR103W, YBR267W, YLR221C, YOR294W, YPL211W, YHR052W, YPR102C, YHR088W, YKL021C, YHR197W, YKR081C, YFR001W, YDR091C, YGL099W, YNL002C
1.41e-22	preribosome, large subunit precursor	YGL111W, YGL078C, YOL077C, YOR206W, YKL172W, YLR002C, YKL009W, YCL054W, YGR103W, YLR221C, YOR294W, YPL211W, YHR052W, YHR088W, YKR081C, YFR001W, YNL061W, YDR087C, YNL002C, YNL224C
2.19e-21	RNA processing	YGL111W, YNL308C, YMR239C, YGL078C, YFL002C, YKL172W, YLR276C, YLR002C, YDL166C, YMR229C, YDL148C, YDR299W, YDR324C, YKL009W, YKL099C, YCL054W, YNL182C, YGR103W, YIR005W, YLR129W, YOR243C, YNL124W, YGR128C, YGR081C, YOR294W, YPL211W, YHR088W, YPR137W, YKL021C, YHR197W, YKR081C, YHR072W-A, YFR001W, YDR091C, YHR148W, YNL061W, YDR087C, YGR178C, YLR409C, YPR112C, YNL002C, YOL010W, YNL207W, YPR144C, YNL075W, YPL126W, YLR186W, YNL224C, YGR159C
3.37e-20	ribonucleoprotein complex	YGL111W, YGL078C, YOL077C, YFL002C, YOR206W, YKL172W, YLR002C, YMR229C, YDL148C, YDR299W, YDR324C, YKL009W, YKL099C, YCL054W, YNL175C, YGR103W, YLR221C, YIR005W, YLR129W, YNL124W, YGR128C, YGR081C, YPL226W, YGR054W, YOR294W, YPL211W, YHR052W, YPR102C, YHR088W, YPR137W, YKR081C, YFR031C-A, YHR072W-A, YFR001W, YDR091C, YAL035W, YHR148W, YNL061W, YDR087C, YGR178C, YLR409C, YPR112C, YNL002C, YOL010W, YPR144C, YNL075W, YPL126W, YLR186W, YNL224C
9.28e-20	cellular component	YGL111W, YNL308C, YMR239C, YGL078C, YHR066W, YOL077C, YFL002C, YOR206W, YKL172W, YLR276C, YLR002C, YDL166C,

	biogenesis	YMR229C, YIR026C, YDL148C, YDR299W, YDR324C, YKL009W, YKL099C, YCL054W, YNL182C, YGR103W, YBR267W, YLR221C, YIR005W, YLR129W, YOR243C, YNL124W, YGR128C, YHR170W, YGR081C, YPL226W, YOR294W, YPL211W, YHR052W, YPR102C, YHR088W, YPR137W, YKL021C, YHR197W, YKR081C, YHR072W-A, YFR001W, YDR091C, YHR148W, YNL061W, YDR087C, YGL099W, YGR178C, YLR409C, YPR112C, YNL002C, YOL010W, YNL207W, YPR144C, YNL075W, YPL126W, YLR186W, YNL224C, YGR159C

Perturbation centralities were calculated with the Turbine software as described in **Methods** of the main text. Term enrichment analysis was performed with the R plug-in of g:Profiler [12], which returns both the enriched terms, and the proteins connected with the term. A term was stated as statistically significant, if the resulting p-value was strictly less than 0.05 after applying Bonferroni correction. The list was cut at $p=10^{-20}$ for brevity of the table. The full list can be obtained by running the „stressed_profile.R" script available at the Turbine web-site http://turbine.linkgroup.hu. The data suggests a very strong down-regulation of ribosome biogenesis and protein translation in all types of stress, which is a well-studied change in stress [16–18] successfully identified by the perturbation centrality measure.

Table S6. Run-time and memory usage of the Turbine program during simulation of different synthetic networks

Network[1]	Number of nodes	Number of links	Run-time[2]	Peak memory usage	Disk space usage	Data-on-disk[3]
BA-100/2	100	200	0.03 s	4,452 kB	796 kB	no
BA-1K/2	1,000	2,000	0.33 s	11,892 kB	7,908 kB	no
BA-10K/2	10,000	20,000	4.16 s	86,008 kB	78,980 kB	no
BA-100K/2	100,000	200,000	50.24 s	828,292 kB	771 MB	no
BA-100K/2	100,000	200,000	51.36 s	45,476 kB	771 MB	yes
BA-1M/2	1,000,000	2,000,000	8 m 32 s	421,640 kB	7713 MB	yes
BA-1M/6	1,000,000	6,000,000	14 m 3 s	921,668 kB	7797 MB	yes

[1]Networks were generated with the "grown" plug-in of the *netgen* tool of the Turbine toolkit. This plug-in creates Barabási–Albert-type scale-free networks given the total requested number of nodes and the degree of newly added nodes.
[2]Run-times are reported as measured by Turbine. This is only the raw simulation time, without accounting for loading and saving the file to/from the hard disk in the cases, where data-on-disk[3] mode is switched off. 1000 time steps were simulated with the communicating vessels model, on a server with a Xeon 3.6 GHz processor, using only one CPU. Column-major matrix ordering was used for better performance. The run-time is linear function of both the number of nodes and the simulation time. Note that the calculation of the perturbation centrality measure for all nodes in a network requires a separate simulation for each node, thus having a quadratic computational complexity with respect to the number of nodes.
[3]Data-on-disk mode is a special feature of Turbine enabling long simulations that would otherwise not fit in the computer memory. In this mode, data files are accessed directly on the disk without consuming any memory, at the price of lower access speed. The performance penalty for using data-on-disk mode is actually surprisingly small according to the data in the Table. Using row-major ordering with data-on-disk mode leads to lower performance, because sequential writes in each time step will become random writes on the disk.

Table S7. Details of the used realizations of modular benchmark graphs

A. Networks used for testing fuzzy versus pronounced modules (all except the ITM-Probe series)					
**Pre-set ratio of inter-modular links**	**Random seed**	**Number of links**	**Number of intra-modular links**	**Number of inter-modular links**	**Overlap[a]**
5%	10	13491	12819	672	No
5%	10	13695	13326	369	Yes
5%	19	13583	12872	711	No
5%	19	13755	13614	141	Yes
5%	20	13938	13237	701	No
5%	20	13583	13392	191	Yes
5%	42	13966	13263	703	No
5%	42	14206	13942	264	Yes
5%	85	13619	12919	700	No
5%	85	13908	13830	78	Yes
5%	87	13772	13086	686	No
5%	87	13884	13595	289	Yes
5%	88	13818	13134	684	No
5%	88	13772	13445	327	Yes
40%	10	13491	8111	5380	No
40%	10	13695	9590	4105	Yes
40%	19	13583	8140	5443	No
40%	19	13755	10444	3311	Yes
40%	20	13938	8361	5577	No
40%	20	13583	10052	3531	Yes
40%	42	13966	8383	5583	No
40%	42	14206	10331	3875	Yes
40%	85	13619	8170	5449	No
40%	85	13908	10746	3162	Yes
40%	87	13772	8269	5503	No
40%	87	13887	9913	3974	Yes
40%	88	13818	8292	5526	No
40%	88	13772	9748	4024	Yes
**B. Networks used in comparing ITM-Probe and Turbine with different fuzziness values**					
**Pre-set ratio of inter-modular links**	**Random seed**	**Number of links**	**Number of intra-modular links**	**Number of inter-modular links**	**Overlap**
5%	59	7717	7319	398	No
5%	87	7824	7429	395	No
5%	88	7788	7395	393	No
10%	59	7692	6916	776	No
10%	87	7837	7054	783	No
10%	88	7820	7035	785	No
15%	59	7755	6581	1174	No
15%	87	7837	6653	1184	No
15%	88	7827	6653	1174	No
20%	59	7780	6213	1567	No
20%	87	7852	6279	1573	No
20%	88	7850	6269	1581	No
25%	59	7795	5844	1951	No
25%	87	7850	5891	1959	No

25%	88	7824	5861	1963	No
30%	59	7776	5438	2338	No
30%	87	7849	5485	2364	No
30%	88	7857	5499	2358	No
35%	59	7803	5059	2744	No
35%	87	7850	5103	2747	No
35%	88	7857	5102	2755	No
40%	59	7792	4670	3122	No
40%	87	7848	4710	3138	No
40%	88	7858	4710	3148	No
45%	59	7805	4292	3513	No
45%	87	7845	4309	3536	No
45%	88	7849	4305	3544	No
50%	59	7794	3885	3909	No
50%	87	7850	3923	3927	No
50%	88	7858	3937	3921	No
55%	59	7805	3509	4296	No
55%	87	7848	3528	4320	No
55%	88	7850	3524	4326	No
60%	59	7805	3124	4681	No
60%	87	7850	3127	4723	No
60%	88	7858	3147	4711	No
65%	59	7797	2716	5081	No
65%	87	7849	2750	5099	No
65%	88	7852	2743	5109	No
70%	59	7803	2340	5463	No
70%	87	7850	2359	5491	No
70%	88	7851	2340	5511	No
75%	59	7801	1939	5862	No
75%	87	7852	1961	5891	No
75%	88	7853	1963	5890	No
80%	59	7803	1556	6247	No
80%	87	7849	1572	6277	No
80%	88	7855	1560	6295	No
85%	59	7805	1166	6639	No
85%	87	7852	1169	6683	No
85%	88	7857	1180	6677	No

The used networks were generated with the benchmark network generator tool of Lancichinetti and Fortunato [1] with the random seed ("seed.dat" file) set to the value in the second column. The exact commands used for generation were the following:

```
benchmark -N 4000 -k 6 -maxk 100 -mu %fuzziness% -t1 3 -t2 0.2 -minc 20 -maxc 100 -on 200 -om 2
benchmark -N 4000 -k 6 -maxk 100 -mu %fuzziness% -t1 3 -t2 0.2 -minc 20 -maxc 100 -on 0 -om 0
```

for networks in part A with and without overlapping communities, respectively. Networks in part B were generated with the command

```
benchmark -N 1000 -k 15 -maxk 50 -mu %fuzziness% -t1 2 -t2 1
```

The variable %fuzziness% was substituted with the corresponding values of the first column in all commands.

[a]"Yes" indicates that the network was generated with overlapping modules, that is, 200 nodes were selected as "overlapping nodes" having two parent communities and receiving intra-modular links from both of them. "No" indicates that no such nodes were present in the network.

Table S8: Average perturbation centrality of inter-modular nodes and inter-modular hubs as compared to the average perturbation centrality of intra-modular, non-hub nodes

**Networks**[a]	**Relative perturbation centrality of inter-modular non-hubs**[b]	**Relative perturbation centrality of intra-modular hubs**[b]
Benchmark graphs with pronounced modules[e]	126%	115%
Benchmark graphs with fuzzy modules	102%	149%
Substrate-bound Met-tRNA synthetase protein structure network	118%	126%
Substrate-free Met-tRNA synthetase protein structure network	113%	125%
Filtered Yeast Interactome	96%	273%
Database of Interacting Proteins yeast interactome (release 2010)	129%	193%
Database of Interacting Proteins yeast interactome (release 2005)	153%	256%
E. coli metabolic network	90%	479%
B. aphidicola metabolic network	112%	281%
School friendship network	107%	149%
**Mean and standard error**	**115% (5.81%)**	**215% (35.6%)**

Perturbation centralities were calculated as described in **Methods** for each node of the network, and their average was taken. Hubs were nodes with a degree in the top 10%. Modularization was performed using the ModuLand Cytoscape plug-in [19]. Inter-modular nodes were defined as nodes having more than 40% inter-modular edges.

[a]Network descriptions are given in **Supplementary Methods** of **Text S1**.

[b]Percentages reported are the percentage of the average perturbation centrality of the node set marked in the description of the column header compared to the average perturbation centrality of intra-modular, non-hub nodes.

Table S9. Signaling residues and residues of experimentally verified importance in Met-tRNA synthetase protein structure network

A. List of individual perturbation properties of residues with experimentally verified importance [5]				
**Residue**	**High importance in the substrate-free conformation**	**High importance in the substrate-bound conformation**	**High increase of importance on binding**	**High decrease of importance on binding**
ASP-456			**Y**	
ASN-452				
ASP-353	**Y**	Y		
ALA-352	**Y**	Y		Y
TYR-357	Y	**Y**		
LEU-355				
TYR-359				
ARG-356	**Y**	**Y**		
ARG-395				
ASN-348				
PHE-350				
HIS-349				
ASP-351	Y	Y		
**TRP-461**	Y		Y	
ASP-449				
TYR-358				Y
THR-360	**Y**	**Y**		
ALA-361	**Y**	**Y**		
LYS-362	Y	**Y**		
SER-354	Y	Y		
**B. List of individual perturbation properties of residues predicted to participate in intra-protein signaling [5]**				
**Residue**	**High importance in the substrate-free conformation**	**High importance in the substrate-bound conformation**	**High increase of importance on binding**	**High decrease of importance on binding**
GLN-538				Y
VAL-543				
PHE-484				
MET-488				
PHE-437	**Y**	**Y**		
ASP-456			**Y**	
ASN-452				
LEU-495	Y	Y		
TRP-432	**Y**	**Y**		
THR-499	Y			Y
HIS-21				
ARG-36	**Y**	**Y**		
ASP-32	**Y**	**Y**		
**LEU-13**	**Y**	**Y**		
PHE-377				**Y**
VAL-381				
PHE-84	**Y**	**Y**		
ASN-391				
LEU-392				

PHE-87	Y	Y		
ILE-89	**Y**	**Y**		
ASP-384	Y	Y		
MET-25	**Y**	Y		
LYS-388		Y		
TYR-357	Y	**Y**		
ILE-385	Y	Y		
HIS-323	Y	Y		
LEU-26		Y	Y	
HIS-28	**Y**	**Y**		
LEU-355				
TYR-531				
VAL-326			Y	
TYR-359				
TRP-346				
LEU-498				Y
MET-333			**Y**	Y
ARG-395				
PHE-350				
TYR-37	**Y**	**Y**		
**TRP-461**	Y		Y	
LYS-492	Y	Y		
LEU-363	**Y**	**Y**		

The list of amino acids of *E. coli* Met-tRNA synthetase predicted to participate in the transmission of conformational changes was taken according to the text and Figure 5 of the paper of Ghosh and Vishveshwara [5]. The list of amino acids with experimentally verified importance was taken from the same article [5] listing earlier findings. Protein structure networks of the substrate-free and substrate-bound forms of *E. coli* Met-tRNA synthetase were constructed as described in **Supplementary Methods**. Perturbation centralities were calculated as described in **Methods** of the main text. Differences of perturbation centralities were obtained by subtracting the *rank* of the perturbation centrality of the substrate-bound conformation from the *rank* of the perturbation centrality of the substrate-free conformation. Residues in the top 20% of largest perturbation centrality in either the substrate-free or the substrate-bound conformation, and residues having an increase or decrease of perturbation importance in the top 20% are shown in the respective column marked by letter "Y". Boldface Y-s signify a top 10% level of importance. Starting and ending residues of the predicted communication pathways, Leu-13 and Trp-461 were also marked with bold letters.

Supplementary Results

Connection of the communicating vessels model with perturbation dissipation using shortest paths and having an exponential decay

In the communicating vessels model the change of the energy of a node is given by the following differential equation:

$$\frac{dS}{dt} = -\sum_{i=0}^{l}\left(\frac{S - S_i}{2} w_i\right) - D_0 \quad (1)$$

where S is the energy of the current node, l is the number of edges of the current node, w_i is the weight of the ith edge, S_i is the current energy of the node on the other end of the ith edge, and D_0 is a parameter defining the amount of energy dissipated by a node in a given time step.

Let us define the case of unobstructed propagation as $S >> S_i$. The consequence of the above definition is that at any given time step, the amount of perturbation transmitted to neighbors is approximately $S\frac{w_i}{2}$. The amount transferred to the nth neighbor is approximately $S\left(\frac{w_i}{2}\right)^n$ using the same logic, which results in an exponential decline with distance. If $w_i \geq 1$, transfer will stop after a second or less in simulation with energy levels evening out to S/2, S/2 for the two affected nodes, which results in the same exponential decline, if the condition $S >> S_i$ holds for the other nodes as well. If there is a connection between nodes at the same distance from the origin, no energy will be transmitted, since $S = S_i$ if the edge weight from the origin is the same for both nodes. Generally, if there are no large differences between edge weights, nodes with the same distance from the origin will tend to have similar values. If there are multiple edges at a given network shell (i.e. distance from the node where perturbation started) to the next one, this only changes the amount of energy propagated by a proportional amount. According to this logic, we can hypothesize that these perturbations tend to travel on shortest paths.

The dissipation of energy in time will be exponential in the unobstructed case. This comes from integrating equation (1) with the condition $S >> S_i$, which yields the result

$$S(t) = \frac{S_0 W_d}{2} e^{-\frac{W_d t}{2}} - \frac{2D_0}{W_d} \qquad (2),$$

which describes an exponential decay, if the weighted out-degree, $W_d = \sum_{i=0}^{l} w_i > 0$.

If the flow of perturbation is totally obstructed (all nodes in the neighborhood have the same energy level) then $S = S_i$, thus $S(t) = S_0 - D_0 t$, which is a simple linear dissipation with time, resulting in no energy transfer with distance. Most real world cases are somewhere between the above two extremes.

Module entrapment occurs when the energy level of a module is too high for the number and weight of inter-modular edges to propagate the perturbation outwards the module, so the energy level inside the module is raised to $S_j = S_i$ for every *i* and *j* node inside the module.

Comparison of the communicating vessels model with the random walk model of ITM-Probe

ITM-Probe is an algorithm modeling information propagation in complex networks using a random walk model [2]. For the comparison of the communicating vessels model used by Turbine with the ITM-Probe method, perturbation centralities obtained on networks with different ratios of inter-modular edges were compared with the "number of visited nodes" measure of the emitting model of the ITM-Probe method as described in the Supplementary Methods section.

The "number of visited nodes" measure used by the emitting model of the ITM-Probe method is theoretically quite similar to the perturbation centrality of the communicating vessels model used by Turbine, since in the ITM-Probe model the random walk has a defined probability (with a default value of 15%) to end at every node visited. This means that the average distance of the last node of the random walk is proportional with the distance from the originating node, so in the end the nodes closer to the origin tend to dissipate more walks in ITM-Probe [2] in the same manner as nodes closer to the origin dissipate more energy in our communicating vessels model.

In the comparison of Turbine with ITM-Probe a total of 51 benchmark graphs [1] were tested, with a ratio of inter-modular edges increasing from 0.05 to 0.85 with steps of 0.05. Three networks were generated with each ratio of inter-modular edges value, with three different random seeds to make the results more robust as described in **Supplementary Methods**. Perturbation centrality and the "number of visited nodes measure" were calculated for each node (1,000 in each network).

In the first simulation perturbation centrality and the "number of visited nodes" measure were averaged for all the 3 randomly generated networks for each inter-modular edge ratio. Spearman correlation of perturbation centrality and the "number of visited nodes" measure was calculated with the R program package [3]. Results are shown in **Figure S1**. Spearman correlation of the average number of visited nodes with the average perturbation centrality calculated for low-intensity perturbations was a perfect 1 meaning that the limitation of perturbation propagation imposed by modular boundaries decreased in exactly the same manner, when assessed by either the Turbine or the ITM-Probe method as the modular structure was coalescing by the gradual increase of the number of inter-modular edges.

Despite the above effect, large differences were observed between the tested measures of ITM-Probe and Turbine. Correlations of degrees, number of visited nodes and perturbation centrality were much lower in different simulations depending on the level of modularity as shown on **Figure S2**. The correlation between perturbation centrality and the "number of

visited nodes" measure (**Figure S2C**) was high when the modularity of the network was noticeable (the ratio of inter-modular edges was smaller than 0.3). However, when the modules started to merge, the correlation diminished, and even turned to negative at the highest ratios of inter-modular edges. An explanation of this effect lies in the fact that in the ITM-Probe method, the degree of a node is inversely correlated with the "number of visited nodes" measures at ratios of inter-modular edges higher than 0.2 (**Figure S2B**). The diminished importance of hubs in information spread is rather counter-intuitive. For low ratios of inter-modular edges, the correlation between the "number of visited nodes" measure and node degree was positive, which shows that high-degree inter-modular nodes are the most important information spreaders in highly modular networks, and in the scale-free benchmark graphs nodes having a higher degree also have a higher chance of gaining inter-modular edges. Correlation of perturbation centrality with node degree varied with a much lower effect size ($\Delta r=0.25$), as shown on **Figure S2A**. The differences observed can be explained by the limiting effect of modular boundaries on perturbation propagation described in the main text.

Figure S3 illustrates the individual perturbation centralities and "number of visited nodes" measures for a single scale-free benchmark graph generated using the random seed value of 87. Perturbation centrality was positively correlated with the node degree in all ratios of inter-modular edges analyzed, but the limiting effect of modular boundaries on the propagation of perturbations was noticeable at low ratios of inter-modular edges. This effect was evident by the segregation of perturbation centrality data to stripes at the ratio of inter-modular edges of 0.05. At this benchmark graph configuration nodes had only one or two inter-modular edges, if any. The top segregated layer of perturbation centrality data corresponds to nodes having two inter-modular edges, while the middle layer of perturbation centrality values corresponds to nodes having one inter-modular edge. The highest "number of visited nodes" values did not change with growing ratios of inter-modular edges (cf. Panels G through L of **Figure S3**), only the lowest "number of visited nodes" values – and thus the average – got higher as ratio of inter-modular nodes increased, resulting in a saturation-like effect for the "number of visited nodes" measure possibly explaining the inverse correlation with degree in networks with highly overlapping modules.

Dissipation-free propagation of perturbation is characterized by fill time, which reciprocally correlates with closeness centrality

Fill time was defined as a measure assessing the propagation of a continuous perturbation in the communicating vessels model without dissipation as stated in the main text. The fill time of node *i* is the time needed to raise the energy level of 80% of the nodes above 1 unit (0.01% of initial perturbation) in a simulation, where a perturbation of 10,000 units was added to node *i* in each time step, and was propagated without dissipation. Fill time was calculated for all nodes in several benchmark graphs and in real-world networks. **Table S1** shows that the reciprocal of the fill time of node *i* strongly correlated with the closeness centrality of the same node ($\bar{r} = 0.895; 95\%CI = 0.843 - 0.946; p = 2.1 \cdot 10^{-11}$, one-sample t-test, Shapiro–Wilk normality test successful with p=0.178). We note that the two metabolic networks, where the correlation with the fill time was the lowest, correspond to a different class of networks according to Guimerà *et al.* [20] Since the closeness centrality of node *i* is defined as the mean geodesic distance (mean shortest path) between node *i* and all other nodes, the high correlation between the reciprocal of fill time and closeness centrality meant that the shortest paths determined most of the dissipation-free propagation of perturbations. This agrees with the expectations [21], and validates the use of the communication vessels model, which has this property as shown in the **Supplementary Results** of **Text S1**.

Comparing the effect of degree and modular position on perturbation centrality in real-world networks

In the experiment comparing the silencing times of the Lancichinetti [1] benchmark networks, the ratio of perturbation dissipation efficiencies of two node categories out of the 4, namely: inter-modular non-hubs and intra-modular hubs, had interesting changes with the modular structure. As we noted earlier, modules of real world networks seem to be more overlapping than the pronounced modules of our benchmark graphs. Starting from these notions we compared the mean perturbation centrality of intra-modular hubs against inter-modular non-hubs as the percentage of the mean perturbation centrality of intra-modular non-hubs in multiple real-world networks (**Table S8**). Hubs were nodes with the top 10% degree, and inter-modular non-hubs were those with at least 40% inter-modular edges – just as in the previous calculations. Inter-modular non-hubs had only a 15% larger perturbation centrality, while hubs had a 115% larger perturbation centrality than intra-modular non-hubs. The large (87%) difference between the effect of hubs versus the effect of inter-modular non-hubs suggest that from a perturbation perspective real-world networks resemble the benchmark graphs with fuzzy modules more, than the benchmark graphs with pronounced modules. (Note that the same observation was obtained when we compared the low-intensity and high-intensity silencing times – see **Table S2.**)

Amino acids participating in intra-protein signaling have a high perturbation centrality in the protein structure network

We have assessed the perturbation centrality of amino acids forming α-helices, β-sheets and loops in two pairs of protein structure networks corresponding to the substrate-free and substrate-bound conformations of *E. coli* Met-tRNA synthetase and rabbit cytochrome P450 2B4, respectively. The Wilcoxon rank-sum test (α=0.00625 adjusted with Bonferroni correction) indicated significantly (p=0.00023, 0.00015, 0.00083, 0.0014 for the free and bound conformations of Met-tRNA synthetase and cytochrome P450, respectively) larger perturbation centralities of α-helices compared to the global mean, while the same test indicated significantly (p=$3.2*10^{-6}$, $9.5*10^{-6}$, $2.3*10^{-6}$, 0.0001 for the free and bound conformations of Met-tRNA synthetase and cytochrome P450, respectively) smaller perturbation centralities for loops compared to the global mean. (**Figure S6A through S6D**; α=0.001). The average perturbation centrality for β-sheets showed a larger variation. The same data calculated for betweenness and closeness centralities is shown on **Figures S7** and **S8** of **Text S1**. Betweenness centrality had a much larger deviation than perturbation centrality, and both closeness and betweenness centralities could differentiate less between amino acids in different secondary structures than perturbation centrality.

We continued the analysis of protein structure networks by assessing the perturbation centralities of *E. coli* Met-tRNA synthetase amino acids participating in intra-protein signaling. We selected Met-tRNA synthetase, since an earlier molecular dynamics study [5] identified key amino acids involved in the transmission of conformational changes upon substrate binding (termed as “Signaling residues” on **Figures S6E** and **S6F**). We have also checked a set of residues, whose importance have already been experimentally established [5] termed as “Experimental residues” on **Figures S6E** and **S6F**). **Figures S6E** and **S6F** show that the perturbation centrality was significantly (Wilcoxon rank-sum test, α=0.0125 adjusted with Bonferroni correction for a FWER of 0.05) higher for both the Signaling (p=$1.7*10^{-6}$, $1.1*10^{-6}$ for the free and Met-tRNA-bound conformations, respectively) and Experimental

residues (p=0.0072, 0.061 for the free and Met-tRNA-bound conformations, respectively) than average. Data in **Figures S7** and **S8** of **Text S1** show that both closeness and betweenness centralities could differentiate Signaling residues, but neither closeness nor betweenness centralities could differentiate the Experimental residues from the average centrality of the whole protein. 67 or 60% of Signaling or Experimental residues, respectively, were in the top 20% of amino acids having the largest perturbation centrality and/or the largest change of perturbation centrality upon substrate binding (**Table S9** of **Text S1**).

Testing the effects of edgetic perturbations

The definition of perturbation centrality described in the main text: "the reciprocal of silencing time retrieved by using a Dirac delta type starting perturbation of 10*n units, where n is the number of nodes in the network, using a dissipation value of 1 can be extended to describe the perturbation centrality of an edge, where the perturbation centrality of the edge connecting nodes i and j is the reciprocal of the silencing time obtained when the same perturbation was started from nodes i and j at the same time. Testing these edgetic perturbations on benchmark networks revealed that edgetic perturbations show the same basic properties as (single) node-based perturbations (cf. **Figure 1** and **Figure S10** of **Text S1**).

Supplementary Methods

Description of the networks used

Benchmark graphs

Scale-free, modular benchmark graphs were generated using the unweighted, undirected benchmark graph generating tool of Lancichinetti and Fortunato [1]. Double edges were removed from the generated networks. Random seed values of 59, 87 and 88 were used to generate three sets of networks. When 7 sets of networks were generated, the additional seeds 19, 20, 42 and 85 were used. These benchmark graphs had 4,000 nodes and 13,785 ± 421 edges. The ratio of inter-modular edges was set to 0.05 in the case of the networks termed as "networks with pronounced modules", and 0.4 for the networks termed as "networks with fuzzy modules". Networks with no overlapping nodes were used in all cases except for the testing of inter-modular nodes (**Figure 2** of the main text), where 200 overlapping nodes were generated, each belonging to two separate modules. A total of 28 networks of this type were created. For the ITM-Probe simulations in this Supplementary Information the number of nodes was 1,000 and the number of edges was 7,825 ± 133 with the ratio of inter-modular edges varying between 0.05 and 0.85 in steps of 0.05 for a total of 51 networks. None of the networks generated for the ITM-Probe comparison contained overlapping nodes. Detailed information about the generated benchmark graphs, as well as the exact commands used for generation is available in **Table S7**. Benchmark graph data can be downloaded from our web-site: http://turbine.linkgroup.hu.

Protein structure networks

The protein structure networks of *Escherichia coli* Met-tRNA synthetase were generated from the starting and equilibrated state of the molecular simulation of the *E. coli* Met-tRNA synthetase/tRNA^{Met}/Met-AMP complex corresponding to the substrate-free and substrate-bound forms of the enzyme, respectively. The structure for the substrate-free form of *E. coli* Met-tRNA synthetase is available (pdb ID: 1QQT) [22]. However, the substrate-bound conformation containing tRNA^{Met} and Met-ATP was not experimentally solved yet, but was created using molecular simulation software using the known substrate-bound structure of *Aquifex aeolicus* Met-tRNA synthase as a template, as described and kindly shared by Ghosh and Vishveshwara [5]. The protein structure network was obtained from the PDB data with the help of the RINerator software [23], which uses the Probe program [24] for calculating interaction strengths. Probe returns negative values for repulsive interactions; we have taken the absolute value of the interaction strength for all interactions, since both repulsive and attractive interactions transmit perturbations. In contrast with the previous article of Szalay-Bekő et al [19], where multiple edges adversely affect calculations, in Turbine, multiple edges are neutral, so the full network was used with every edge and self-loop intact instead of averaging the strengths of multiple links into a single one. The rationale behind this is to retain as much information as possible from the original file. The protein structure network had 547 nodes, since the first 3 N-terminal amino acids were not participating in the network and all ligands and cofactors, including the tRNA were removed. The final weighted, undirected protein structure networks of the substrate-free and substrate-bound enzyme contained 6,901 and 6,744 edges, respectively.

Protein structure networks of the substrate-free and substrate-bound forms of rabbit cytochrome P450 2B4 protein were also created with the RINerator software [23] by using the 1PO5 and 1SUO pdb files of the Protein Data Bank, and taking the absolute value of the resulting interaction strengths. The surrounding water molecules, the ligands and the cofactors were removed from the networks, resulting in 465 nodes with 6,278 edges in the substrate-free (open) conformation and 465 nodes with 6,409 edges in the substrate-bound (closed) conformation, both undirected, weighted networks. Protein structure network data can be downloaded from our web-site: http://turbine.linkgroup.hu.

Yeast protein-protein interaction networks

The Filtered Yeast Interactome (FYI) is a high-fidelity yeast protein-protein interaction dataset containing data consistently obtained using several different methods [6]. The downloadable data set in the Supplement of the article contained 1,302 proteins (nodes) having 2,312 interactions (edges). The giant component of this network had 695 nodes and 1,614 edges.

The "Database of Interacting Proteins yeast interactome (release 2005)" network is the giant component of the unweighted and undirected yeast protein-protein interaction network assembled by Ekman et al. [25] using the 2005 March compilation of the Database of Interacting Proteins [26] consisting of 2,444 nodes and 6,271 edges covering approximately half the proteins of yeast genome. Besides the rather high confidence of its data, we choose this network, because it was used in the identification of party and date hubs, an interesting dynamic feature of protein-protein interaction networks [25] and its properties were assessed in our earlier publications [19].

The "Database of Interacting Proteins yeast interactome (release 2010)" network was created from the 2010 October compilation of the Database of Interacting Proteins [26]. Only high-fidelity interactions marked as "core" were included in the network, yielding a giant component of 1,884 nodes and 4,234 edges.

Interaction weights of yeast proteins were obtained from the yeast whole-genome mRNA expression dataset of Holstege et al. [27] containing data of 6,180 yeast genes (5461 with expression levels, and one gene with two different expression levels). Missing data (719 nodes total, less than 12%) were substituted as the ln-transformed average expression level of all other proteins, 0.9205, taking into account that the distribution of expression data is approximately lognormal [28]. For calculating the changes of expression levels in different types of stress, the datasets of Gasch *et al.* [29] were used, which describe the relative changes of expression levels based on a set of microarray data. Thus, the stressed expression levels were calculated by multiplying the Holstege baseline with the Gasch relative changes according to the previous article [30]. The particular datasets used for the different stresses were the following: "Heat Shock 15 minutes hs-1" (25°C to 37°C heat shock for 15 minutes) for the heat-shocked network; "constant 0.32 mM H_2O_2 (30 min) redo" (0.32 mM hydrogen-peroxide treatment for 30 minutes) for the oxidative stress, and "1 M sorbitol - 15 min" (hyper-osmotic shock using 1 M sorbitol for 15 minutes) for the osmotic stress. Detailed experimental data is available in the article describing the dataset [29]. Edge-weights of non-stressed and stressed protein-protein interaction networks were generated from the expression data by multiplying the abundances of the two connected proteins as described earlier [30], since larger concentrations of involved proteins make their interaction more likely. Thus all final interactomes were weighted and undirected.

The use of even larger interactomes such as BioGRID or STRING were also considered, but was dropped due to computational constraints, since there were 6 magnitudes of difference between weighted out-degrees. Adding the fact that the weight distribution was approximately lognormal (so most of the nodes had low weighted outdegrees), and the required constraint that the maximum weighted outdegree should be no more than 1 ($-1 \leq \Delta t \sum_{i=0}^{l} w_i \leq 1$) meant that unrealistically high analysis times would have been required to attenuate the perturbations, since enforcing an upper limit for the maximum weighted outdegree results in median weighted outdegrees becoming extremely low, which in turn makes propagation speed disproportionately slow. Protein-protein interaction network data can be downloaded from our web-site: http://turbine.linkgroup.hu.

Metabolic networks

Metabolic networks were created by Balázs Szappanos (Biological Research Centre, Hungarian Academy of Sciences, Szeged, Hungary), and were the same as used in the papers of Mihalik and Csermely [30] and Szalay-Bekő *et al.* [19]. *Escherichia coli* metabolic network contained 249 metabolites (nodes) and their 730 transformations (edges), while the *Buchnera aphidicola* metabolic network contained 190 nodes and 563 edges. These networks were constructed based on the primary data of Feist *et al.* [9] and Thomas *et al.* [8], respectively. Frequent cofactors were deleted from the networks, except of those metabolic reactions, where cofactors were considered as main components. For the better comparison of networks, metabolic reactions were taken irreversible, and flux balance analyses (FBA) were performed resulting in weighted networks. All flux quantities were minimized, whereas reactions non-affecting the biomass production were considered having zero flux. Weights were generated as the mean of the appropriate flux quantities in absolute value, except the case when one of the fluxes was zero that automatically resulted in a zero weight [30]. For *E. coli*, data from rich medium was used to make the metabolic network more similar to the network of *B. aphidicola*. Final networks were thus weighted and undirected. Metabolic network data can be downloaded from our web-site: http://turbine.linkgroup.hu.

School friendship network

The social network was community-44 from the Add Health survey[2] as described by James Moody [31] and Mark Newman [32]. This network has an approximately equal number of black and white students and 4 well-developed, rather dense communities. The network contains 1,147 students with 6,189 directed edges between them. In our current study directed parallel edges were merged into a single undirected edge with a weight equal to the sum of the original weights, and only the giant component of the network was used. This process resulted in a weighted undirected network consisting of 1,127 nodes and 5,096 edges with weights between 1 and 12. Network data can be downloaded from our web-site: http://turbine.linkgroup.hu.

[2]This research uses data from Add Health, a program project designed by J. Richard Udry, Peter S. Bearman, and Kathleen Mullan Harris, and funded by a grant P01-HD31921 from the National Institute of Child Health and Human Development, with cooperative funding from 17 other agencies. Special acknowledgment is due Ronald R. Rindfuss and Barbara Entwisle for assistance in the original design. Persons interested in obtaining data files from Add Health should contact Add Health, Carolina Population Center, 123 W. Franklin Street, Chapel Hill, NC 27516-2524 (addhealth@unc.edu).

Network measures and their calculation

The degree of a node is defined as the number of edges of the node. More precisely for the perturbation simulations we have to consider the number of outgoing edges (i.e. the out-degree). However, all networks we used were undirected, so the degree of a node was equivalent to its out-degree in all cases. Weighted degree was calculated by summarizing the edge weights of all edges for a given node. Degree and weighted degree were calculated using the built-in algorithm of Turbine.

PageRank is a random-walk based measure [33], where the outbound edges of a node increase the centrality of those nodes for which the given edge is an inbound edge. The exact value of increase is proportional to the current PageRank value of the edge's outbound node. Multiple iterations of this method yielded a converging result [33]. PageRank values were calculated using the algorithm of the igraph [34] package.

Closeness centrality [35] is defined as the mean geodesic distance (mean shortest path) between a given node and all other nodes reachable from it. Betweenness centrality [36] gives the (relative) number of shortest paths between every two nodes in the network, which include the examined node. Closeness centrality and betweenness centrality were calculated using the Pajek [37] package.

Community centrality is a measure coined for the ModuLand overlapping community structure determination program [19], which measures the centrality of a node separately for different communities. Community centrality was calculated by the ModuLand Cytoscape plug-in [19].

Network modularization

Community structures of networks were determined by the ModuLand Cytoscape plug-in [19] with basic settings. A threshold of 0.9 was used for merging highly correlated modules in all networks. For the school friendship network, modules of the second hierarchical level were used (where meta-nodes of the level represent modules of the original network and meta-edges of the level represent the overlap of the modules of the original network as described in [19]) to obtain the 4 densely connected communities of the network [32,38].

Determination of the "number of visited nodes" measure of the ITM-Probe method

To compare the "number of visited nodes" measure of the ITM-Probe method [39] with the perturbation centrality obtained by using the Turbine program, the recently released standalone version of the ITM-Probe method [39] was used, since calculating the ITM-Probe results for all nodes on a network would take a tremendous amount of time using the web-based interface. A plug-in was written for Turbine that converts any complex network in Turbine format (needed for the fast-calculation of the Turbine program) to the format required by ITM-Probe (JSON). This plug-in can be downloaded from the Turbine web-site at http://turbine.linkgroup.hu. The ITM-Probe method was used with its emitting model with a damping factor of 0.85. The main script file of the ITM-Probe method was executed separately for every single node in a certain network, and all output was concatenated into a single text file. The "number of visited nodes" measures were extracted from the resulting ITM Probe text file with an awk script.

Supplementary References

1. Lancichinetti A, Fortunato S (2009) Benchmarks for testing community detection algorithms on directed and weighted graphs with overlapping communities. Phys Rev E 80: 016118.

2. Stojmirovi A, Yu Y-K, Stojmirović A (2009) ITM Probe: analyzing information flow in protein networks. Bioinformatics 25: 2447–2449.

3. R Core Team (2012) R: A language and environment for statistical computing. Available: www.r-project.org.

4. Bastian M, Heymann S, Jacomy M (2009) Gephi: An open source software for exploring and manipulating networks. International AAAI Conference on Weblogs and Social Media.

5. Ghosh A, Vishveshwara S (2007) A study of communication pathways in methionyl-tRNA synthetase by molecular dynamics simulations and structure network analysis. Proc Natl Acad Sci U S A 104: 15711–15716.

6. Han J-DJ, Bertin N, Hao T, Goldberg DS, Berriz GF, et al. (2004) Evidence for dynamically organized modularity in the yeast protein-protein interaction network. Nature 430: 88–93.

7. Xenarios I (2002) DIP, the Database of Interacting Proteins: a research tool for studying cellular networks of protein interactions. Nucleic Acids Res 30: 303–305.

8. Thomas GH, Zucker J, Macdonald SJ, Sorokin A, Goryanin I, et al. (2009) A fragile metabolic network adapted for cooperation in the symbiotic bacterium Buchnera aphidicola. BMC Syst Biol 3: 24.

9. Feist AM, Henry CS, Reed JL, Krummenacker M, Joyce AR, et al. (2007) A genome-scale metabolic reconstruction for Escherichia coli K-12 MG1655 that accounts for 1260 ORFs and thermodynamic information. Mol Syst Biol 3: 121.

10. Schrödinger LLC (2010) The PyMOL molecular graphics system. Available: www.pymol.org.

11. Zhong Q, Simonis N, Li Q-R, Charloteaux B, Heuze F, et al. (2009) Edgetic perturbation models of human inherited disorders. Mol Syst Biol 5: 321.

12. Reimand J, Arak T, Vilo J (2011) g:Profiler -- a web server for functional interpretation of gene lists (2011 update). Nucleic Acids Res 39: W307–W315.

13. Parrou J, Teste M, François J (1997) Effects of various types of stress on the metabolism of reserve carbohydrates in *Saccharomyces cerevisiae*: genetic evidence for a stress-induced recycling of glycogen and trehalose. Microbiology 143: 1891–1900.

14. François J, Parrou J (2001) Reserve carbohydrates metabolism in the yeast *Saccharomyces cerevisiae*. FEMS Microbiol Rev 25: 125–145.

15. Gasch A (2003) The environmental stress response: a common yeast response to diverse environmental stresses. In: Hohmann S, Mager WH, editors. Yeast Stress Responses. Berlin: Springer. pp. 11–57.

16. Lindquist S (1981) Regulation of protein synthesis during heat shock. Nature 293: 311–314.

17. Mayer C, Grummt I (2005) Cellular stress and nucleolar function. Cell Cycle 4: 1036–1038.

18. Rudra D, Warner JR (2004) What better measure than ribosome synthesis? Genes Dev 18: 2431–2436.

19. Szalay-Beko M, Palotai R, Szappanos B, Kovacs IA, Papp B, et al. (2012) ModuLand plug-in for Cytoscape: determination of hierarchical layers of overlapping network modules and community centrality. Bioinformatics 28: 2202–2204.

20. Guimerà R, Sales-Pardo M, Amaral LAN (2007) Classes of complex networks defined by role-to-role connectivity profiles. Nat Phys 3: 63–69.

21. Maslov S, Ispolatov I (2007) Propagation of large concentration changes in reversible protein-binding networks. Proc Natl Acad Sci U S A 104: 13655–13660.

22. Mechulam Y, Schmitt E, Maveyraud L, Zelwer C, Nureki O, et al. (1999) Crystal structure of *Escherichia coli* methionyl-tRNA synthetase highlights species-specific features. J Mol Biol 294: 1287–1297.

23. Doncheva NT, Klein K, Domingues FS, Albrecht M (2011) Analyzing and visualizing residue networks of protein structures. Trends Biochem Sci 36: 179–182.

24. Word JM, Lovell SC, LaBean TH, Taylor HC, Zalis ME, et al. (1999) Visualizing and quantifying molecular goodness-of-fit: small-probe contact dots with explicit hydrogen atoms. J Mol Biol 285: 1711–1733.

25. Ekman D, Light S, Björklund AK, Elofsson A (2006) What properties characterize the hub proteins of the protein-protein interaction network of *Saccharomyces cerevisiae*? Genome Biol 7: R45.

26. Xenarios I, Rice DW, Salwinski L, Baron MK, Marcotte EM, et al. (2000) DIP: the database of interacting proteins. Nucleic Acids Res 28: 289–291.

27. Holstege FC, Jennings EG, Wyrick JJ, Lee TI, Hengartner CJ, et al. (1998) Dissecting the regulatory circuitry of a eukaryotic genome. Cell 95: 717–728.

28. Irizarry RA, Hobbs B, Collin F, Beazer-Barclay YD, Antonellis KJ, et al. (2003) Exploration, normalization, and summaries of high density oligonucleotide array probe level data. Biostatistics 4: 249–264.

29. Gasch AP, Spellman PT, Kao CM, Carmel-Harel O, Eisen MB, et al. (2000) Genomic expression programs in the response of yeast cells to environmental changes. Mol Biol Cell 11: 4241–4257.

30. Mihalik Á, Csermely P (2011) Heat shock partially dissociates the overlapping modules of the yeast protein-protein interaction network: a systems level model of adaptation. PLoS Comput Biol 7: e1002187.

31. Moody J (2001) Race, school integration, and friendship segregation in America. Am J Sociol 107: 679–716.

32. Newman MEJ (2003) The structure and function of complex networks. SIAM Review 45: 167–256.

33. Page L, Brin S, Motwani R, Winograd T (1998) The PageRank citation ranking: Bringing order to the web. Stanford Digital Library Technologies Project: 1–17.

34. Csardi G, Nepusz T (2006) The igraph software package for complex network research. InterJournal Complex Systems: 1695.

35. Sabidussi G (1966) The centrality index of a graph. Psychometrika 31: 581–603.

36. Newman M (2010) Networks: An Introduction. New York: Oxford University Press.

37. Bagatelj V, Mrvar A (2002) Graph drawing. In: Mutzel P, Jünger M, Leipert S, editors. Pajek – Analysis and Visualization of Large Networks. Berlin: Springer. pp. 77–103.

38. Kovács IA, Palotai R, Szalay MS, Csermely P (2010) Community landscapes: an integrative approach to determine overlapping network module hierarchy, identify key nodes and predict network dynamics. PLoS ONE 5: e12528.

39. Stojmirović A, Bliskovsky A, Yu Y-K (2012) CytoITMprobe: a network information flow plugin for Cytoscape. BMC Res Notes 5: 237.
